# A human Tau expressing zebrafish model of progressive supranuclear palsy identifies Brd4 as a regulator of microglial synaptic elimination

**DOI:** 10.1038/s41467-024-52173-0

**Published:** 2024-09-18

**Authors:** Qing Bai, Enhua Shao, Denglei Ma, Binxuan Jiao, Seth D. Scheetz, Karen A. Hartnett-Scott, Vladimir A. Ilin, Elias Aizenman, Julia Kofler, Edward A. Burton

**Affiliations:** 1 Department of Neurology, University of Pittsburgh, Pittsburgh, PA 15213 USA; 2 Pittsburgh Institute for Neurodegenerative Diseases, University of Pittsburgh, Pittsburgh, PA 15213 USA; 3 Tsinghua University School of Medicine, Beijing, China; 4 Department of Neurobiology, University of Pittsburgh, Pittsburgh, PA 15213 USA; 5 Department of Pathology, University of Pittsburgh, Pittsburgh, PA 15213 USA; 6 Alzheimer’s Disease Research Center, University of Pittsburgh, Pittsburgh, PA 15213 USA; 7 Geriatrics Research, Education and Clinical Center, Pittsburgh VA Healthcare System, Pittsburgh, PA 15240 USA

**Keywords:** Neurodegeneration, Movement disorders

## Abstract

Progressive supranuclear palsy (PSP) is an incurable neurodegenerative disease characterized by 4-repeat (0N/4R)-Tau protein accumulation in CNS neurons. We generated transgenic zebrafish expressing human 0N/4R-Tau to investigate PSP pathophysiology. Tau zebrafish replicated multiple features of PSP, including: decreased survival; hypokinesia; impaired optokinetic responses; neurodegeneration; neuroinflammation; synapse loss; and Tau hyperphosphorylation, misfolding, mislocalization, insolubility, truncation, and oligomerization. Using automated assays, we screened 147 small molecules for activity in rescuing neurological deficits in Tau zebrafish. (+)JQ1, a bromodomain inhibitor, improved hypokinesia, survival, microgliosis, and brain synapse elimination. A heterozygous *brd4*^+/−^ mutant reducing expression of the bromodomain protein Brd4 similarly rescued these phenotypes. Microglial phagocytosis of synaptic material was decreased by (+)JQ1 in both Tau zebrafish and rat primary cortical cultures. Microglia in human PSP brains expressed Brd4. Our findings implicate Brd4 as a regulator of microglial synaptic elimination in tauopathy and provide an unbiased approach for identifying mechanisms and therapeutic targets in PSP.

## Abstract (web summary)

Progressive supranuclear palsy (PSP) is an incurable neurodegenerative disease characterised by accumulation of 4R-Tau protein in the brain. Transgenic zebrafish overexpressing human 4R-Tau showed rapid PSP-like phenotypes, allowing an unbiased small molecule screen coupled with reverse genetics to identify Brd4-dependent microglial synapse removal as a mechanism mediating neurological phenotypes.

## Introduction

Progressive supranuclear palsy (PSP) is a sporadic neurodegenerative disease characterized by falls, hypokinesia, dysphagia, oculomotor deficits and dementia^1,2^. Median onset is age 67^3^, median survival is 8 years^4^, and there are no effective treatments. Pathologically, neuronal loss in brainstem and basal ganglia nuclei is accompanied by microglial activation and formation of neuronal intracellular inclusions composed of insoluble, hyperphosphorylated aggregates of the microtubule-associated protein Tau^5^. Accumulation of Tau protein in PSP predominantly involves isoforms containing 4 microtubule-binding domain repeats and lacking N-terminal insertions (0N/4R-Tau)^6,7^ (this contrasts with Alzheimer’s disease and chronic traumatic encephalopathy, in which multiple 3R- and 4R-Tau isoforms are deposited). Genetic evidence supporting a causative role for 4R-Tau in PSP includes: clinical-pathologic PSP phenocopies caused by missense and splice site mutations of the MAPT gene^8^ encoding Tau; a locus inversion allele encompassing the MAPT gene that expresses less 4R-Tau^9^ and is protective against PSP^10^; rare instances of MAPT gene duplications in PSP^11^; and a strong association between PSP risk and non-coding SNPs near to MAPT^12,13^. However, to date, interventions targeting Tau directly have not prevented PSP progression in clinical trials^14–16^. The reasons for this are uncertain; one possibility is that disease progression in trial participants with clinically established PSP is driven by engagement of additional mechanisms conceptually downstream of 4R-Tau accumulation. Understanding how 4R-Tau disrupts CNS function to cause neurological deficits is therefore an important objective with translational potential.

Unbiased discovery-driven approaches such as small molecule screens may help elucidate the molecular pathophysiology of PSP, particularly as the consequences of 4R-Tau accumulation in the nervous system are incompletely understood. In addition, many first-in-class drugs were discovered by phenotypic screening^17^, an important consideration given the lack of therapeutic options for PSP patients. An unbiased small molecule screen would require a high-throughput model that replicates the complexities of PSP pathophysiology – which occurs in end-differentiated neurons, involves cell-autonomous and non-autonomous mechanisms, and expresses clinical endpoints by disrupting the functions of neural circuits. These considerations suggest that a screen should be completed in vivo to capture relevant pathogenic events. Zebrafish (*Danio rerio*) show substantial genetic and neurological homology to humans and, uniquely amongst vertebrates, offer opportunities for chemical library screening in vivo^18,19^. Notably, screening in a zebrafish PSP model might employ automated assays of disease-relevant neurological phenotypes as readouts, in order to identify small molecule modifiers without bias concerning mechanism of action. However, although there has been significant interest in the development of transgenic zebrafish expressing human Tau for this purpose^20^, most of the published lines express mutated forms of human Tau that are not present in PSP^21,22^ and none of the current models has been documented to show PSP-like phenotypes with automated assay outputs suitable for unbiased screening^21–25^. Consequently, a phenotype-driven small molecule screen using a zebrafish tauopathy model has not yet been reported.

Here, we generate a transgenic zebrafish model that over-expresses human 0N/4R-Tau, and replicates many clinical, biochemical and pathological features of PSP. Importantly, several of these phenotypes are sufficiently rapid and robust to use as screening endpoints. We interrogate a library of 147 small molecule inhibitors of epigenetic readers, writers and erasers against neurological phenotypes in Tau zebrafish and find that (+)JQ1 (a bromodomain inhibitor) rescues motor function, improves survival, and prevents microgliosis, synapse loss, and microglial phagocytosis of synaptic material. We use CRISPR genome editing to generate a null allele in the gene encoding the bromodomain protein Brd4 and find that the resulting *brd4*^+/−^ mutant also rescues motor function, microgliosis and synapse loss in Tau zebrafish. We further show that Brd4 is expressed in microglia in the human CNS. Our work identifies Brd4 as a regulator of microglial synapse elimination in tauopathy, and more broadly provides tools and methodology to investigate PSP pathophysiology and therapeutics through an unbiased discovery-driven approach.

## Results

### Generation of transgenic zebrafish expressing human 0N/4R-Tau conditionally in neurons

**Fig. 1:**
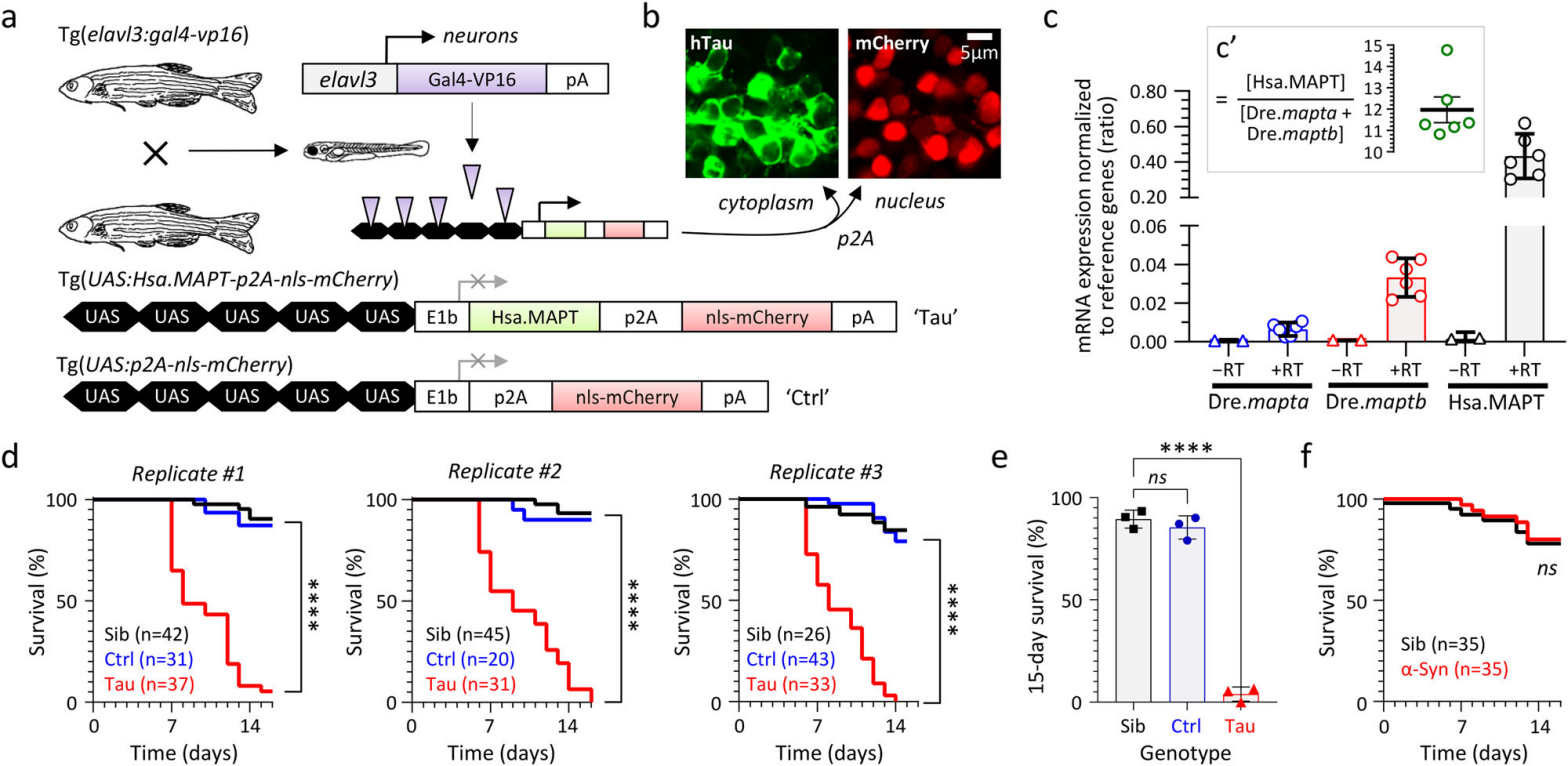
Reduced lifespan of transgenic zebrafish expressing human 4R/0N-Tau conditionally in neurons. **a** Expression of human 0N/4R-Tau and an mCherry reporter conditionally in neurons of transgenic Tau zebrafish using Gal4/UAS genetics. The control UAS construct, which lacks the Hsa.MAPT transgene, is shown below for comparison. **b** Cytoplasmic expression of human 4R-Tau and nuclear expression of mCherry (to allow rapid non-invasive genotyping) as separate proteins from the same bicistronic hsa.MAPT-p2a-nls-mCherry mRNA. **c** Hsa.MAPT, Dre.*mapta* and Dre.*maptb* mRNA expression in Tau zebrafish, normalized to the reference genes Dre.*bact1* and Dre.*gapdh* at 5dpf by real-time quantitative RT-PCR Each data point shows the mean of three technical replicates for a single biological replicate. Bars show mean ± SE of the six biological replicates. RNA samples that were not reverse transcribed (−RT) are shown as controls. The inset panel c’ shows the ratio of transgene Tau to the sum of the two endogenous paralogues in each biological replicate, bars show mean ±95% CI. See Supplementary Figs. S2–S5 for details. **d** Survival curves for Tau zebrafish in comparison with non-expressing siblings (Sib), and Ctrl zebrafish expressing nls-mCherry only. Three replicate cohorts are shown, n for each experimental group is indicated in the graph legends. ****p < 10^−15^ Tau *vs*. Ctrl, or Tau *vs*. Non-Tg, Mantel–Cox test (Supplementary Table 4). **e** 15-day survival in the three biological replicate cohorts shown in panel **d**. Bars show mean ± SE, points show % survival for each replicate. ****p = 8.7 × 10^−7^, 1-way ANOVA with Dunnett’s multiple comparisons test. **f** Survival curves for zebrafish expressing human α-Synuclein (Syn; *n* = 35) from a similar UAS construct and under the same Gal4 driver as Tau zebrafish, in comparison with non-expressing siblings (*n* = 35). *ns*, not significant Syn *vs*. Sib, Mantel–Cox test (Supplementary Table 5). Source data are provided as a Source Data file.

To develop a zebrafish PSP model optimized for small molecule screening applications, we employed Gal4-UAS genetics (Fig. 1a, Supplementary Fig. S1). The UAS responder cassette encodes wildtype (WT) human 0N/4R-Tau (which accumulates in PSP), together with nls-mCherry to allow rapid identification of transgenic zebrafish by fluorescence microscopy in downstream applications. The p2A peptide^26^ ensures that human 0N/4R-Tau and nls-mCherry are expressed as separate proteins (directed to different subcellular compartments; Fig. 1b) from the same mRNA transcript, thereby avoiding the potential for a fluorescent fusion protein to alter the pathophysiological properties of Tau. By generating Tg(*UAS:Hsa.MAPT-p2A-nls-mCherry*) lines in the absence of Gal4-induced expression, we isolated stable heritable transgene alleles that can be propagated readily. We then selected lines that showed robust transactivation when crossed with a pan-neuronal Gal4 driver^27^ to yield Tg(*elavl3:Gal4-VP16*); Tg(*UAS:Hsa.MAPT-p2A-nls-mCherry*) ‘Tau’ zebrafish. This selection strategy resulted in lines that express human MAPT mRNA at approximately 12-fold abundance in whole zebrafish lysate compared with the endogenous zebrafish Tau paralogues^28^ *mapta* and *maptb* (Fig. 1c; Supplementary Figs. S2–S5; Supplementary Tables 1 and 2). This may be an over-estimate at a cellular level, since the duplicated zebrafish genes show spatially restricted CNS expression during development^28^, in contrast to the strong pan-neuronal *elavl3* driver^27^. Tg(*elavl3:Gal4-VP16*); Tg(*UAS:p2A-nls-mCherry*) ‘Ctrl’ zebrafish were generated to provide controls with the same transgenes as Tau zebrafish but lacking human 0N/4R-Tau (Fig. 1a; Supplementary Table 3).

### Impaired survival, neurodegeneration, and neuroinflammation in Tau zebrafish

**Fig. 2:**
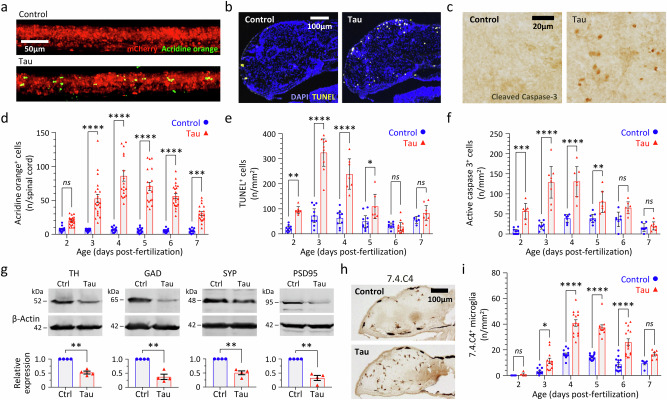
Neurodegeneration, synapse loss, and microgliosis in transgenic zebrafish expressing human 4R/0N-Tau. **a**–**f** Three complementary methods demonstrating cell death in the CNS of Tau and Ctrl zebrafish from 2 to 7 days post-fertilization (dpf): **a**, **d** acridine orange labeling and intravital imaging of spinal cord (*n* = 12–22 zebrafish/group); **b**, **e** TUNEL labeling of brain sections (*n* = 6–10 zebrafish/group); **c**, **f** cleaved caspase 3 labeling of brain sections (*n* = 7–10 zebrafish/group). Example images from each method are shown in (**a**–**c**), and quantification shown in **d**–**f**. Data points show cell counts from individual zebrafish (Ctrl, blue circles; Tau, red triangles). In **e**, **f**, each point is the mean of 10–12 sections for each individual zebrafish analyzed as shown in Supplementary Fig. S8. Bars show group mean ± SE. *p < 0.05, **p < 0.01, ***p < 0.001, ***p < 0.0001 Ctrl *vs*. Tau, 2-way ANOVA (genotype, age) with Šidák multiple comparisons test. **g** Western blots of lysates from pooled Tau or Ctrl larvae at 5dpf probed with antibodies to tyrosine hydroxylase (TH; dopaminergic neurons), glutamic acid decarboxylase (GAD; GABAergic neurons), synaptophysin (SYP; presynaptic terminals) or post-synaptic density protein 95 kDa (PSD95, post-synaptic terminals) and β-actin (loading control). Example blots are shown above, quantification of expression of each marker in Tau zebrafish (red triangles) relative to Ctrl (blue circles) is shown in four biological replicates below. Bars show mean ± SE, **p < 0.01 one sample 2-tailed *t*-test comparing Tau to normalized Ctrl value of 1. **h**, **i** Microgliosis in Tau zebrafish. Brain sections were labeled with antibody 7.4.C4 (a microglial marker; example images in **h**). Brain microglia were counted in serial sections from each zebrafish at time points 2–7 dpf as shown in Supplementary Fig. S8. Data points show mean for 10–12 sections from each individual Tau (red triangles, *n* = 4–15 zebrafish/group) and Ctrl (blue circles, *n* = 4–16 zebrafish/group) zebrafish, bars show group mean ± SE, *p < 0.05, ***p < 0.0001 Ctrl *vs*. Tau, 2-way ANOVA (genotype, age) with Šidák multiple comparisons test. Source data are provided as a Source Data file. Exact *p* values are shown in Supplementary Tables 6–10.

The lifespan of Tau zebrafish (median survival 8–9 days in replicate cohorts) was severely attenuated in comparison with Ctrl zebrafish or non-expressing siblings (both control lines survive >2 years; Fig. 1d, e; Supplementary Table 4). Additional control zebrafish co-expressing human α-Synuclein and mCherry^29^, or mCherry and GFP, did not show impaired survival, excluding a non-specific artifact of heterologous protein over-expression as the cause of the shortened lifespan in Tau zebrafish (Fig. 1f, Supplementary Fig. S6; Supplementary Table 5). Three complementary assays – failure of acridine orange exclusion in vivo (Fig. 2a, d, Supplementary Table 6), TUNEL labeling of apoptotic cells in histological sections (Fig. 2b, e, Supplementary Table 7), and detection of Capsase-3 cleavage in histological sections (Fig. 2c, f, Supplementary Table 8) and by western blot (Supplementary Fig. S7) – demonstrated a robust increase in neuronal death in Tau zebrafish compared with Ctrl between 2 and 7 days post-fertilization (dpf; see also Supplementary Fig. S8). Maximum cell death was detected in Tau zebrafish in all three assays at 3–4 dpf, with up to 10-fold excess apoptotic cells compared with Ctrl. By 5 dpf, decreases in the expression of dopaminergic (Tyrosine Hydroxylase; TH) and GABAergic (Glutamic Acid Decarboxylase; GAD) neuronal markers, and in both pre- (Synaptophysin; SYP) and post- (Post-Synaptic Density 95 kDa; PSD95) synaptic markers, were apparent in Tau zebrafish compared with Ctrl by western blot (Fig. 2g, Supplementary Table 9). Immunohistochemistry also showed a > 2-fold increase in the density of brain microglia in Tau zebrafish compared with Ctrl between 3 and 6 dpf (Fig. 2h, i, Supplementary Table 10).

Together, these data show that conditional expression of human 0N/4R-Tau in transgenic zebrafish causes impaired survival, neurodegeneration, loss of neurochemical and synaptic markers, and neuroinflammation, which are all key features of PSP.

### Human Tau loses solubility and becomes hyperphosphorylated, mislocalized, misfolded, truncated, and oligomerized in transgenic zebrafish

**Fig. 3:**
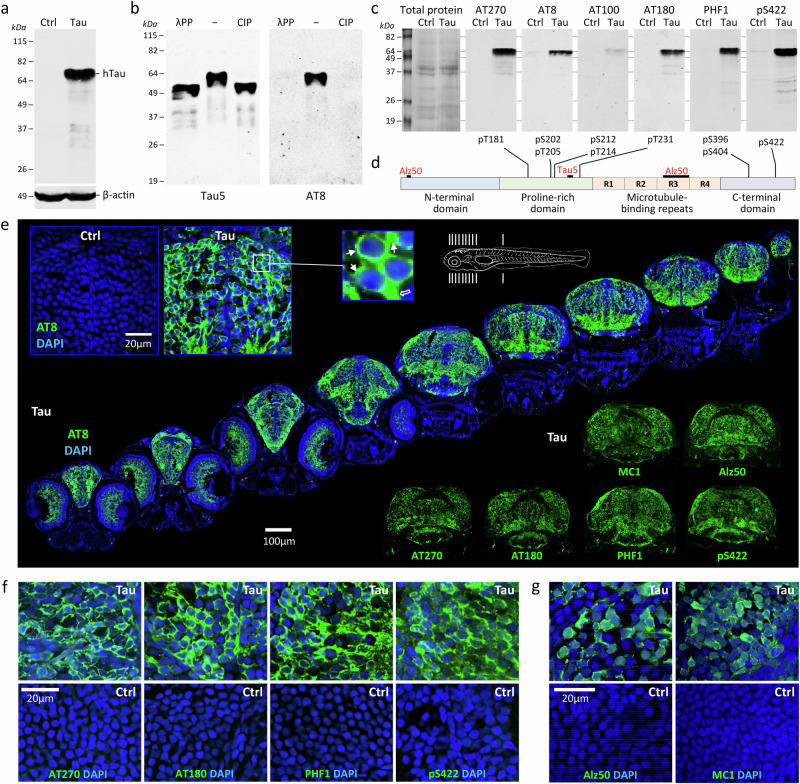
Human 4R/0N-Tau hyperphosphorylation, mislocalization and misfolding throughout the CNS of transgenic Tau zebrafish. **a**, **b** Western blots of zebrafish head region lysates at 3dpf. **a** Samples from Ctrl and Tau zebrafish, blot probed with antibodies to total human Tau and β-Actin. **b** Lysates from Tau zebrafish were pretreated with calf intestinal alkaline phosphatase (CIP), Lambda protein phosphatase (λPP), or no enzyme (−). The blot was probed with an antibody to total human Tau (left) and an antibody to human phosphorylated [pS202, pT205]-Tau (AT8; right). **c** Replicate western blots of lysates from Ctrl and Tau zebrafish were probed with a panel of antibodies specific to different phosphorylated human Tau epitopes. The total protein loading control is shown for the first blot (left; others identical). **d** Major domains of human 4R/0N-Tau are shown to illustrate the locations of epitopes detected by the antibodies used in this study. **e** Serial axial sections (planes indicated in schematic at top) through a Tau zebrafish at 5dpf labeled for human phosphorylated Tau (AT8; green) and a nuclear counter label (DAPI; blue). The inset panels top left show similar sections from Ctrl and Tau zebrafish at higher magnification; the expanded panel illustrates mislocalization of human phospho-Tau to the cell bodies of neurons (small arrows) in addition to physiological localization in axons (large outline arrow). The inset panels below right show sections labeled with antibodies to other human Tau phosphoepitopes and misfolding epitopes as indicated. **f**, **g** Confocal micrographs showing sections from Tau (upper row) and Ctrl (lower row) zebrafish brain at 5dpf, labeled with antibodies recognizing (**f**) phosphorylated human Tau (AT270, AT180, PHF1 and pS422; green) or (**g**) misfolded human Tau (Alz50 and MC1; green), and a nuclear counter label (DAPI; blue). Source data are provided as a Source Data file.

Human 0N/4R-Tau in lysates from Tau zebrafish at 3 dpf migrated on western blot at 64 kDa (Fig. 3a), near the reported electrophoretic mobility of hyperphosphorylated 0N/4R-Tau in human PSP brain^6,7^. Dephosphorylation of Tau zebrafish lysate decreased the apparent molecular mass of 0N/4R-Tau to 52 kDa and eliminated immunoreactivity to a human phospho-Tau-specific antibody, suggesting that human Tau becomes hyperphosphorylated in the zebrafish CNS (Fig. 3b). Consistent with this interpretation, a 64 kDa band in Tau (but not Ctrl) zebrafish lysate was immunoreactive to six different antibodies that detect phospho-Tau epitopes distributed across the proline-rich and C-terminal domains of the human protein (Fig. 3c, d)^30^. Hyperphosphorylated human 4R/0N-Tau was detected in the cell bodies, proximal processes, and axons of neurons throughout the CNS of Tau zebrafish (Fig. 3e, f). Widespread mislocalization of phosphorylated Tau was seen in the perinuclear cytoplasm (Fig. 3e inset panel, and f); this is thought to be an early feature of tauopathy and is a common finding in Tau over-expression models that may reflect disengagement of phosphorylated Tau from microtubules. Many neurons in Tau zebrafish were also labeled by antibodies detecting epitopes specific to misfolded Tau (Alz50, MC1; Fig. 3e, g) in human tauopathy^31^. No evidence was found of human Tau spreading to cells that did not express the transgene (Supplementary Fig. S9), although the lack of available zebrafish Tau antibodies prevented evaluation of whether human Tau seeds can template zebrafish Tau misfolding or aggregation.

**Fig. 4:**
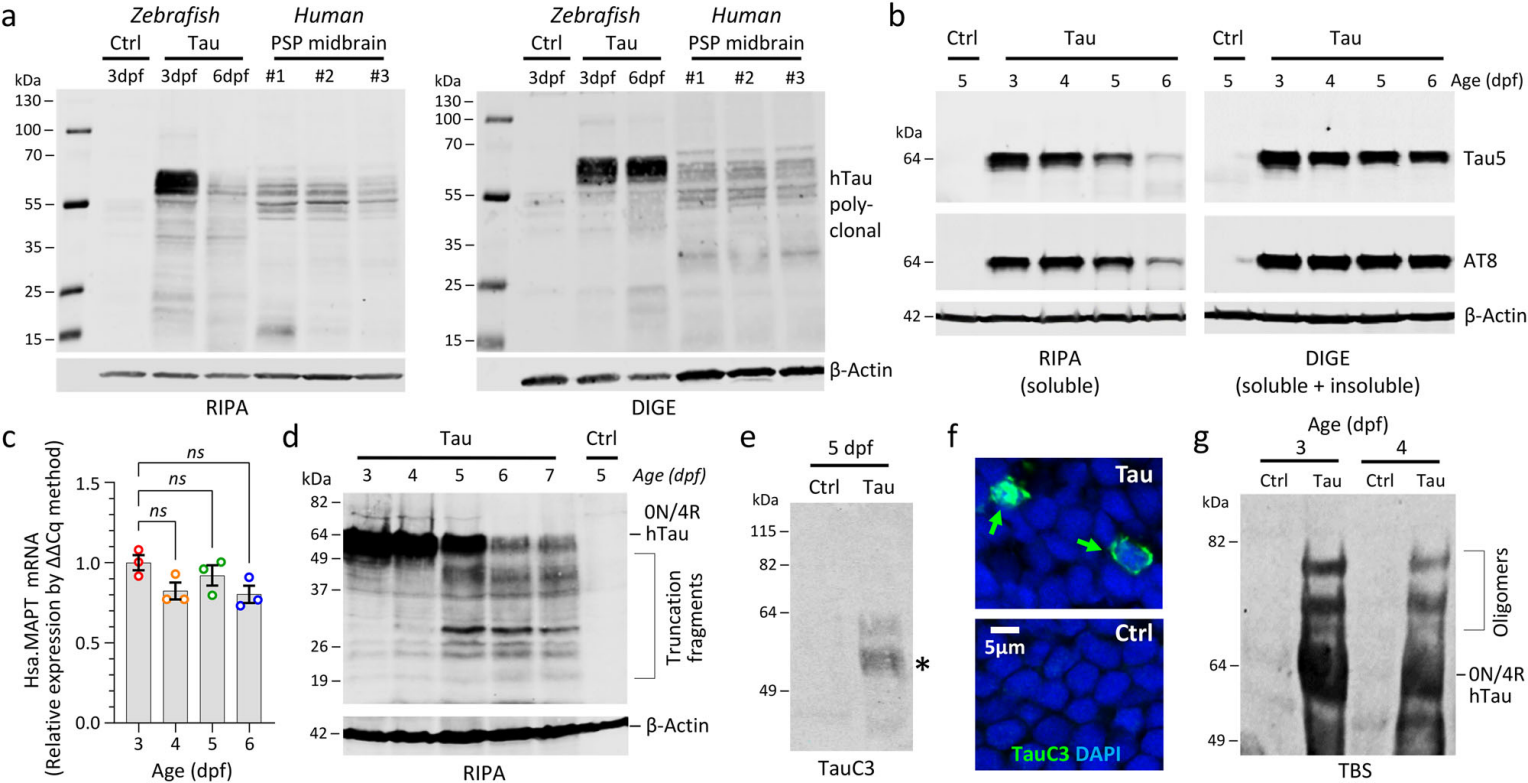
Loss of solubility, truncation, and oligomerization of Human 4R/0N-Tau in transgenic Tau zebrafish. Proteins were extracted from Ctrl or Tau zebrafish pooled head regions, using RIPA (SDS/Triton), DIGE (7 M urea) or TBS (tris-buffered saline) for analysis by western blot. **a** Comparison of Ctrl and Tau zebrafish with postmortem midbrain samples from 3 different PSP patients. Blots probed with antibodies to total human Tau (above) and β-Actin (below). **b** Analysis of Tau zebrafish with increasing age. Blots were probed with antibodies to total human Tau (above), phosphorylated human Tau (AT8; middle) and β-Actin (below). **c** Real-time quantitative RT-PCR quantification of Hsa.MAPT mRNA in Tau zebrafish at 3–6 dpf. Each data point shows a biological replicate (calculated as mean of three technical replicates). Bars show group mean ± SE; ns not significant, 1-way ANOVA with Dunnett’s multiple comparisons test. **d** Western blot similar to **b** but run to resolve lower molecular weight proteins and probed with an antibody to human pS422-Tau. **e** Western blot of Tau and Ctrl zebrafish samples at 5dpf probed with antibody TauC3 that specifically recognizes human Tau truncated by Caspase-3 at residue D421. *Denotes bands with sizes suggestive of 0N/4R-Tau cleavage in Tau zebrafish. **f** Confocal micrographs showing sections from Tau and Ctrl brain labeled with the same TauC3 antibody used in **e** recognizing human Tau truncated at D421 (TauC3; green) and a nuclear counter label (DAPI; blue). Scattered labeled neurons in Tau zebrafish are indicated by green arrows. **g** Western blot of Ctrl and Tau zebrafish samples extracted by mechanical dissociation in tris-buffered saline without reducing agents or heating and probed with an antibody to total human Tau. The expected electrophoretic mobilities of monomeric human 0N/4R-Tau and additional high molecular weight forms corresponding to oligomers are indicated. Source data are provided as a Source Data file.

Western blot analysis to compare Tau zebrafish with human PSP midbrain lysates, extracted with either RIPA (1% Triton, 0.1% SDS) or DIGE (7 M urea), showed co-migration of multiple Tau-immunoreactive bands, further demonstrating similarity between the model and PSP (Fig. 4a). Between 3 and 6 dpf, total and phosphorylated full-length human Tau in RIPA-extracted lysates declined steadily; however, no decrease was noted in DIGE-extracted lysates, suggesting progressive loss of 0N/4R-Tau solubility in the zebrafish brain (Fig. 4a, b). These findings were confirmed by serial RIPA-DIGE extraction (Supplementary Fig. S10) and were not attributable to declining transgene expression, since Hsa.MAPT mRNA levels remained stable over the same time points (Fig. 4c, Supplementary Fig. S11). Truncated fragments of phosphorylated human Tau were apparent with increasing age in the RIPA-soluble fraction from Tau zebrafish by western blot (Fig. 4d). Analysis using an antibody (TauC3)^32^ that specifically recognizes human Tau truncated by Caspase-3 at residue D421 showed faint bands in Tau zebrafish samples, with sizes suggestive of 0N/4R-Tau cleavage at D421 (possibly with other N-terminal cleavage events; Fig. 4e). Scattered neurons in Tau zebrafish brains also showed perinuclear cytoplasmic immunoreactivity for this epitope (Fig. 4f). Non-denaturing/non-reducing gel electrophoresis demonstrated the presence of higher molecular weight species, corresponding to soluble oligomers of truncated Tau (Fig. 4g).

Together the data in Figs. 3 and 4 show that human 0N/4R-Tau in the zebrafish brain loses solubility and becomes hyperphosphorylated, mislocalized, misfolded, truncated, and oligomerized, replicating many features of human tauopathies including PSP. However, we did not find evidence of large argyrophilic amyloid aggregates resembling neurofibrillary tangles over the short lifespan of Tau zebrafish. This is similar to other rapidly progressive tauopathy models^33^, consistent with prominent pre-tangle pathology found in PSP^5^, and possibly related to the relative infrequency of cells immunoreactive for caspase-cleaved Tau (Fig. 4f), which may be a precursor of larger aggregates^34^.

### PSP-relevant neurological phenotypes in Tau zebrafish

**Fig. 5:**
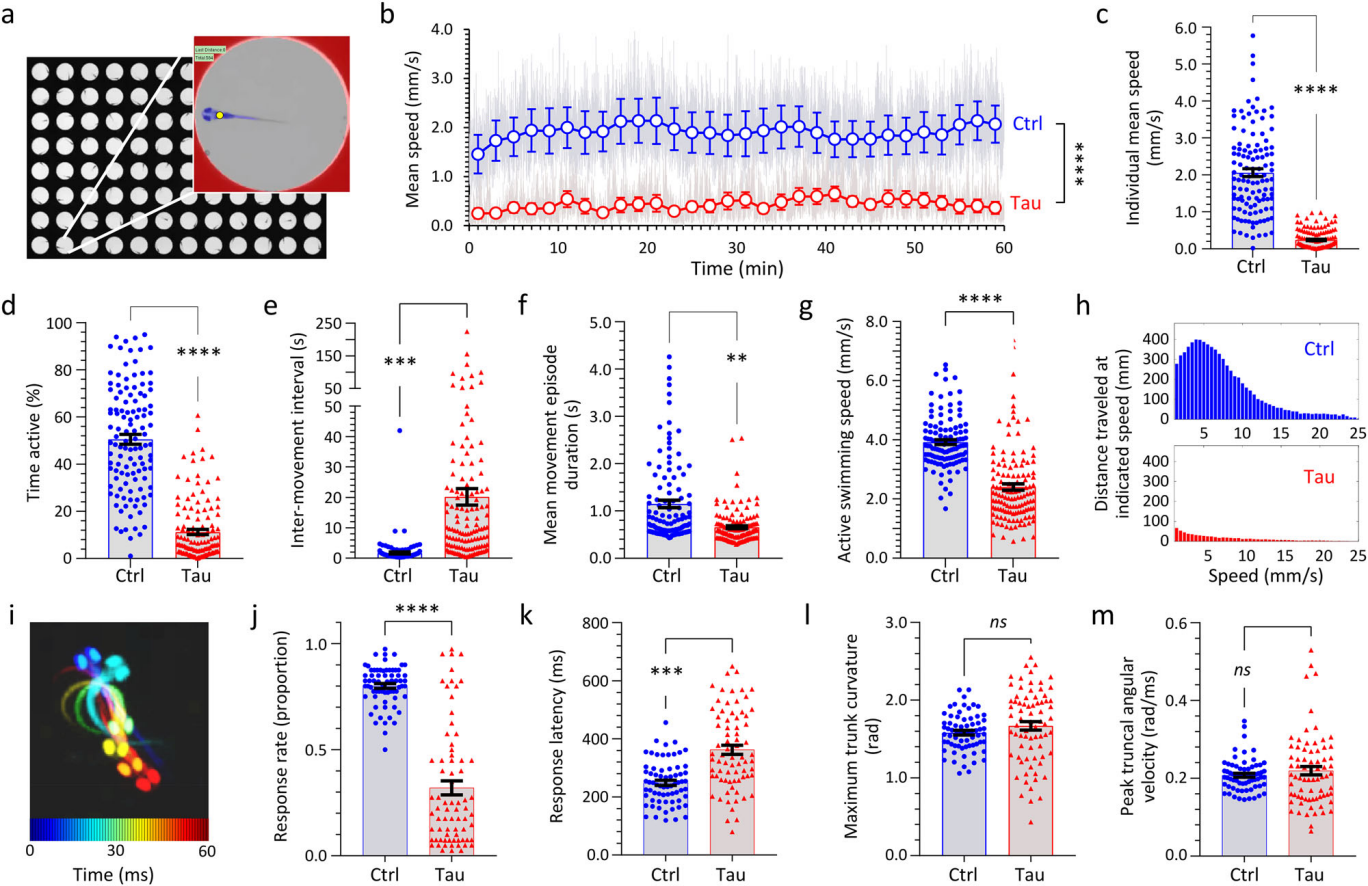
Hypokinetic motor phenotype in transgenic zebrafish expressing human 4R/0N-Tau. **a**–**h** Zebrafish motor function was evaluated at 5 dpf using automated tracking in 96-well plates^35,36^ under constant ambient illumination at 28 °C. **a** Plate image; inset shows features detected by the algorithm (well boundary, red; zebrafish, blue; zebrafish centroid, yellow). **b** Ctrl and Tau zebrafish (*n* = 48 each group) were compared in the same 96-well plate. Zebrafish centroid displacement at each video frame transition is scaled for each group to show mean speed (light gray traces). Colored markers (Ctrl, blue; Tau, red) show group mean ± SE within each 2-min time bin. ****p < 0.0001, Ctrl vs. Tau, 2-way repeated measures ANOVA with Šidák multiple comparisons test (Supplementary Table 11). **c**–**h** Quantification of motor function in Ctrl and Tau zebrafish (3 combined biological replicates): **c** mean speed; **d** % time active; **e** inter-movement interval; **f** movement episode duration; **g** active swimming speed. Data points show individual zebrafish (Ctrl, *n* = 117; Tau, *n* = 130), bars show mean ± SE. ****p < 10^−15^, ***p = 3.1 × 10^−10^, **p = 5.3 × 10^−8^, Ctrl vs. Tau, 2-tailed unpaired *t*-test with Welch’s correction. **h** Histogram showing total distance traveled at different instantaneous speeds over the course of the assay. **i**–**m** Zebrafish responses to abrupt ambient light-dark transitions were evaluated at 5dpf at 1000 frames/s using a segmentation/kinematics application^38^. **i** ‘O’-bend illustrated by superimposing pseudocolored zebrafish silhouettes every 10 ms during the response. **j** Response rate (proportion of stimuli followed by motion within 1 s); **k** response latency (mean interval between stimulus and response onset); **l** maximum trunk curvature during ‘O’-bend response; and **m** peak truncal angular velocity (maximum rate of change of trunk curvature). Data points in **i**–**m** show mean responses of individual zebrafish (Ctrl, *n* = 68; Tau, *n* = 74), bars show group mean ± SE. ****p < 10^−15^, ***p = 7.3 × 10^−9^, *ns* not significant, Ctrl vs. Tau, 2-tailed unpaired *t*-test with Welch’s correction. Source data are provided as a Source Data file.

Automated machine vision measurement of zebrafish motor activity in 96-well plates at 5 dpf (Fig. 5a)^35–37^ showed that Tau zebrafish were profoundly hypokinetic in comparison with Ctrl zebrafish during spontaneous movement in bright ambient illumination over prolonged recording periods (Fig. 5b; Supplementary Table 11). At 5dpf, zebrafish movements are discontinuous and stochastic. Mean scalar speed (centroid displacement/time) is an integrative measurement of zebrafish motility, reflecting both the speed and duration of individual movements and the frequency with which they are executed. A robust decrease in mean speed in Tau zebrafish compared with Ctrl zebrafish was observed in multiple replicate assays (Fig. 5c; Supplementary Fig. S12; Supplementary Table 12) and was attributable to: (i) a large increase in the time interval between movements, combined with a small decrease in the duration of movement events, resulting in a decreased proportion of the assay during which Tau zebrafish were motile (Fig. 5d–f); and (ii) a modest decrease in the speed of individual movements made by Tau zebrafish (Fig. 5g, h). This hypokinetic phenotype was not seen in multiple other controls, including non-expressing siblings, zebrafish expressing GFP only, zebrafish co-expressing mCherry and GFP, or zebrafish co-expressing human α-Synuclein and mCherry, excluding variations in genetic background or a non-specific artifact of heterologous protein over-expression as causes of these motor abnormalities (Supplementary Figs. S13, S14). The kinematics of ‘O-bend’ turning movements evoked by abrupt ambient light-dark transition were next analyzed at high temporal and spatial resolution (Fig. 5i)^38^. The response rate to illumination transitions was significantly lower in Tau zebrafish than controls, and the latency to response was modestly prolonged (Fig. 5j, k). However, when a response occurred, both maximum trunk curvature and peak truncal angular velocity did not differ between Tau and Ctrl (Fig. 5l, m). Together, these data suggest that Tau zebrafish show severe hypokinesia that is not attributable to neuromuscular paralysis but is caused by a reduced frequency of movement initiation events, with possible parallels to the akinetic motor disorder seen in PSP patients.

**Fig. 6:**
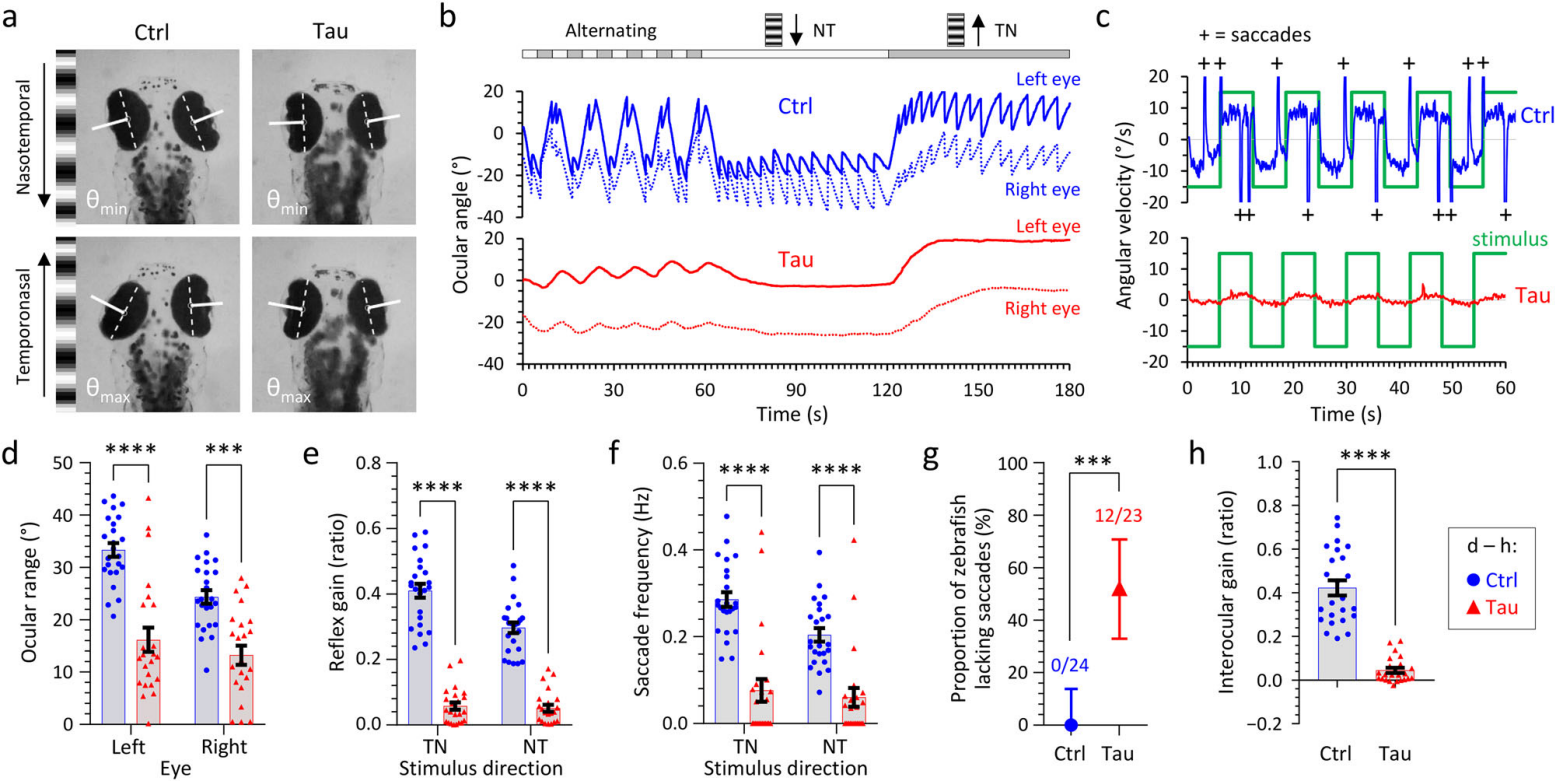
Optokinetic reflex abnormalities in transgenic zebrafish expressing human 4R/0N-Tau. Optokinetic reflexes (OKR) were elicited at 5dpf and detected, measured, and analyzed using an automated tracking system^40^. **a** Single video frames showing the extremes of ocular position after presenting a nasotemporal (above) or temporonasal (below) stimulus to the left eye of Ctrl (left) or Tau (right) zebrafish. The ocular axes detected by the algorithm are shown in white. The difference between maximum and minimum angle is quantified as ocular range in panel **d**. **b** Example nystagmograms showing ocular angle (0 denotes long axis of eye parallel to y-axis of image) for the left (stimulated; solid line) and right (contralateral; broken line) eyes from Ctrl (blue; above) and Tau (red; below) zebrafish in response to successive 1-min periods of alternating (10 s each direction), nasotemporal, and temporonasal stimuli. **c** Comparison of the stimulus angular velocity (green) and the left eye angular velocity for Ctrl (blue; above) and Tau (red; below) zebrafish. High-velocity saccadic positional resetting movements (velocity exceeds axis limits) are indicated by ‘+’ in the Ctrl trace. The relationship between the stimulus and slow phase tracking movements is quantified as reflex gain in panel **e**. The frequency of saccadic movements is quantified in panel **f**. **d**–**h** Quantification of OKR responses in Ctrl and Tau zebrafish. In each graph **d**–**f**, **h**, data points show individual zebrafish (Ctrl, *n* = 24; Tau, *n* = 23), bars show mean ± SE. **d** Ocular range (****p = 8.2 × 10^−10^, ***p = 3.3 × 10^−5^); **e** reflex gain (****p < 10^−15^); **f** saccade frequency (****p = 2.3 × 10^−10^, ***p = 5.3 × 10^−6^); (h) interocular gain (****p = 3.6 × 10^−11^); Ctrl *vs*. Tau, (**d**–**f**) 2-way ANOVA with Šidák multiple comparisons test, **h** 2-tailed unpaired *t*-test with Welch’s correction. **g** Proportion of zebrafish lacking high-velocity saccadic movements; bars show proportion ±95% CI, ***p = 0.000026, 2-sided Fisher’s exact test. Source data are provided as a Source Data file.

In view of the prominent oculomotor disorder that characterizes PSP clinically, we next evaluated the eye movements of Tau zebrafish by analyzing optokinetic reflexes (OKR; Fig. 6)^39,40^. Projection of an animated grating pattern onto a screen filling the zebrafish visual field provokes characteristic cycles of slow ocular movements in the stimulus direction (that serve to stabilize the moving image in the retina) and rapid positional resetting movements (saccades) in the opposite direction (Fig. 6a–c; Supplementary Fig. S15; Supplementary Movie 1). Compared with Ctrl zebrafish, Tau zebrafish showed a decreased range of ocular movement, reflex gain, and saccade frequency, in addition to disrupted coordination between movements of the two eyes (Fig. 6b–h; Supplementary Fig. S15). Complete absence of saccades during OKR was observed in >50% Tau zebrafish (Fig. 6g), resembling a characteristic clinical abnormality found in PSP patients (although vertical eye movement abnormalities, not tested here, are more prominent in PSP)^41^. Together, these data show that Tau zebrafish develop robust OKR deficits reminiscent of those found in PSP.

### Phenotype-based small molecule screen in vivo

**Fig. 7:**
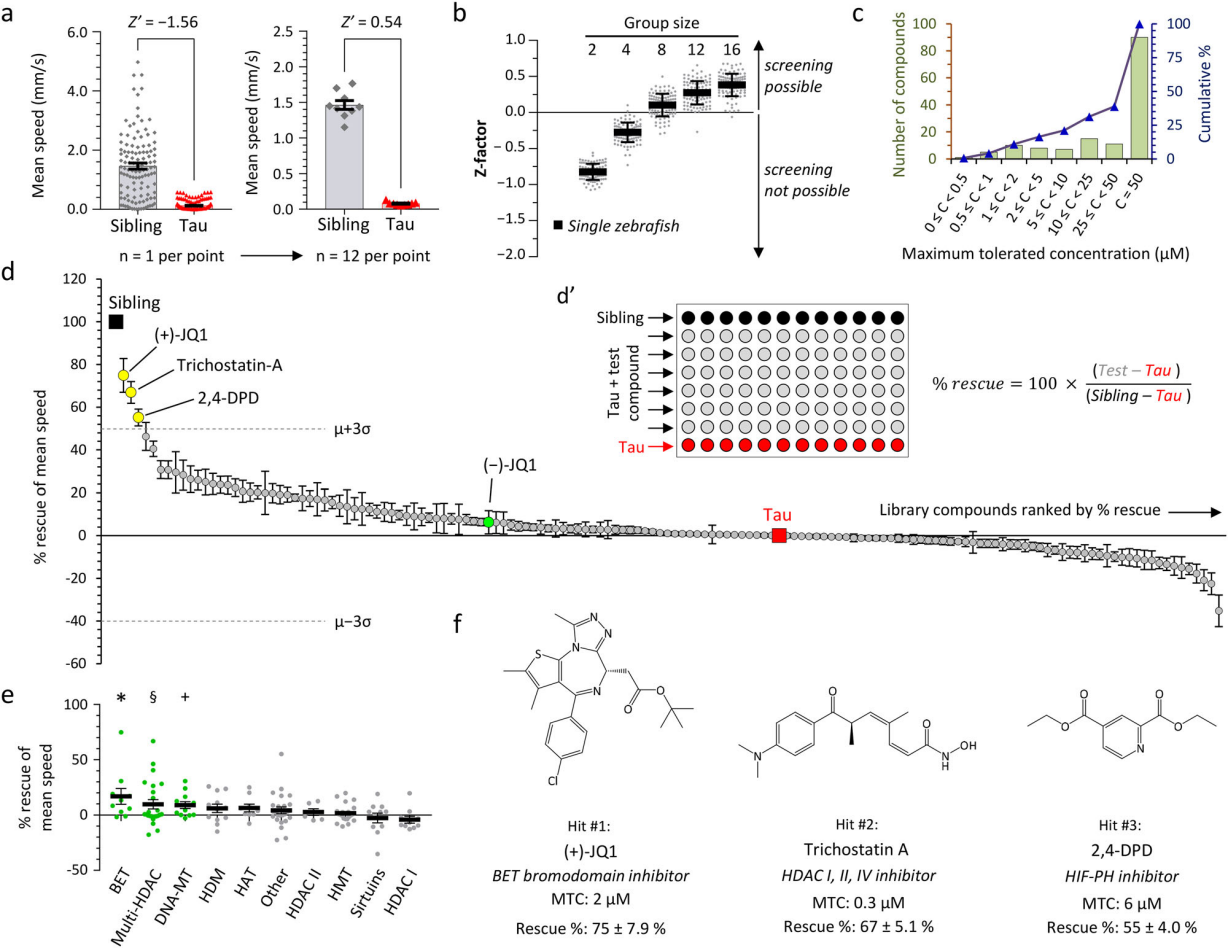
Small molecule screen to identify inhibitors of epigenetic readers, writer and erasers that rescue hypokinesia in transgenic zebrafish expressing human 4R/0N-Tau. **a** Mean speed during the light phase of the visual motor response (VMR) in non-expressing siblings (Sib; *n* = 115) and Tau zebrafish (*n* = 130). Data points show individual zebrafish on the left graph and means of groups of 12 zebrafish on the right; bars show group mean ± SE on both graphs. **b** Z-factor (*Z’*) was calculated from the primary data shown in panel **a**, either for single zebrafish, or for groups containing 2–16 zebrafish, each in 100 different random groupings. Points show different groupings at each group size, bars show mean ±2 SD. **c** Binned distribution (green bars, left scale) and cumulative % distribution (blue line, right scale) of the maximum tolerated concentration (MTC) of 147 small molecule modulators of epigenetic readers, writers, and erasers in larval zebrafish. **d** Screen of 147 small molecule modulators of epigenetic readers, writers, and erasers for rescue of VMR light phase hypokinesia in Tau zebrafish exposed to compounds at MTC from 2dpf to 5dpf. Rescue calculated as shown in inset d’. Data points show % rescue ±SE for each compound (*n* = 12 zebrafish per data point), ordered by rank along the x-axis (details in Supplementary Table 15). The untreated Tau (0% rescue; *n* = 12) and Sib (100% rescue; *n* = 12) groups are indicated. Library mean rescue ±3 SD is indicated; compounds satisfying the a priori definition of a ‘hit’ (library mean +3 SD) are shaded yellow and labeled. (−)JQ1, the inactive stereoisomer of (+)JQ1, is shaded green for comparison. **e** Data from panel (**d**) grouped by biological target. Bars show group mean ± SE; targets showing significant rescue across compounds within a group are shaded green (*p = 0.042, ^§^*p* = 0.028, ^+^*p* = 0.013, group mean *vs*. rescue = 0; 2-tailed, one sample *t*-test). BET bromo- and extra-terminal domain containing proteins, HDAC histone deacetylases, DNA-MT DNA methyltransferases, HDM histone demethylases, HAT histone acetyltransferases, HMT histone methyltransferases, Other, targets listed in Supplementary Table 17. **f** Chemical structures, molecular targets, maximum tolerated concentrations, and activities of the three ‘hits’ identified by the screen. Source data are provided as a Source Data file.

We next investigated whether automated measurement of hypokinesia could be used as an unbiased and quantitative screening endpoint to detect interventions that rescue the neurological phenotype of Tau zebrafish. For these analyses, we compared Tau zebrafish with non-expressing siblings, to simplify breeding for subsequent screening, and to minimize background genetic variability that could adversely affect quantitative assay performance. This approach is valid, as Ctrl zebrafish and non-expressing siblings of Tau zebrafish showed identical responses in 96-well plate motor assays (Supplementary Fig. S13). Variability in measured motor activity was minimized by averaging the evoked responses to multiple cycles of alternating ambient illumination and darkness (visual motor response; VMR; Supplementary Fig. S16; Supplementary Table 13). Mean VMR light phase swimming speed showed the largest and most replicable difference between Tau zebrafish and their siblings in these assays. However, analysis of single zebrafish using this metric as an endpoint did not provide adequate performance as a screening assay (Fig. 7a). In contrast, and similar to our previous findings in pharmacological and toxicant-induced parkinsonism models^36^, averaging the responses of multiple zebrafish in each treatment group substantially improved assay performance at the expense of throughput (Fig. 7b). The optimal replicate size of 12 Tau zebrafish per group allowed 6 compounds to be tested in parallel with controls in each 96-well plate, with a consistently positive Z’ indicating a remarkable level of performance for a neurobehavioral assay (Fig. 7a, b).

We selected a library of 147 small molecule modulators of epigenetic readers, writers, and erasers for a screen using neurological endpoints in Tau zebrafish (Supplementary Table 14). This collection was chosen because the compounds target aspects of cell biology that are relatively unexplored in tauopathy, and the library size was manageable for solving the practical and logistical challenges posed by implementing the screening workflow. Zebrafish were exposed to library compounds from 2 to 5 dpf, and screening was completed in two stages: (i) the maximum tolerated concentration (MTC) of each compound was defined experimentally in WT zebrafish using survival and morphological endpoints (Fig. 7c; Supplementary Fig. S17); (ii) each compound was then tested at MTC in 12 Tau zebrafish, and their mean VMR light phase swimming speed at 5 dpf was normalized to the phenotypic window defined by 12 untreated Tau zebrafish and 12 non-expressing siblings in the same assay (Fig. 7d, d’; Supplementary Fig. S18). These controls provided stringent inbuilt assay QC, and normalization of responses to within-assay controls allowed comparison between assays. Both QC benchmarks and data analysis were automated to eliminate bias and accelerate workflow (Supplementary Figs. S19, S20), and compounds were screened with their identities masked until the entire library had been analyzed.

Mean ± SE % phenotypic rescue for each library compound is shown in Fig. 7d, in comparison with untreated Tau zebrafish (0% rescue) and non-expressing siblings (100% rescue; numerical data, chemical identities, and targets are shown in Supplementary Table 15). Three of the compounds satisfied our a priori definition of a ‘hit’ (mean library rescue +3 SD; Fig. 7d, yellow data points; Fig. 7f; Supplementary Fig. S21), whereas the remainder produced rescue scores that were distributed around the untreated Tau group. The top hit, (+)JQ1, rescued light phase swimming speed by 75% at a bath concentration of 2 µM (Fig. 7d, f). This compound disrupts binding of acetylated lysine residues to the bromodomains of bromo- and extraterminal-domain containing (BET) proteins, which regulate transcriptional responses to histone acetylation. The other two hits were Trichostatin-A (inhibits multiple histone deacetylases, HDACs) and 2,4-DPD (inhibits hypoxia-inducible factor-1α prolyl hydroxylase) (Fig. 7d, f). Clustering the library compounds by pharmacological target showed that BET, multi-HDAC, or DNA methyltransferase inhibitors overall improved motor function significantly in Tau zebrafish (Fig. 7e; Supplementary Fig. S22, Supplementary Tables 16, 17). There was substantial variability between compounds within each target class; for example, not all BET bromodomain inhibitors were active in this assay. This likely reflects variation in the pharmacokinetics of individual compounds, or their differential binding affinity for zebrafish proteins^42^.

In post-screen validation assays, repurchased (+)JQ1, Trichostatin-A, and 2,4-DPD all showed activity in rescuing Tau zebrafish VMR light phase motor function, confirming the identities of the three hits and validating the screening outputs (Supplementary Fig. S23). We focused the remainder of this study on the top hit, (+)JQ1.

### (+)JQ1 rescues motor function, improves survival, prevents microgliosis, and restores expression of PSD95 in Tau zebrafish through a specific pharmacological action

**Fig. 8:**
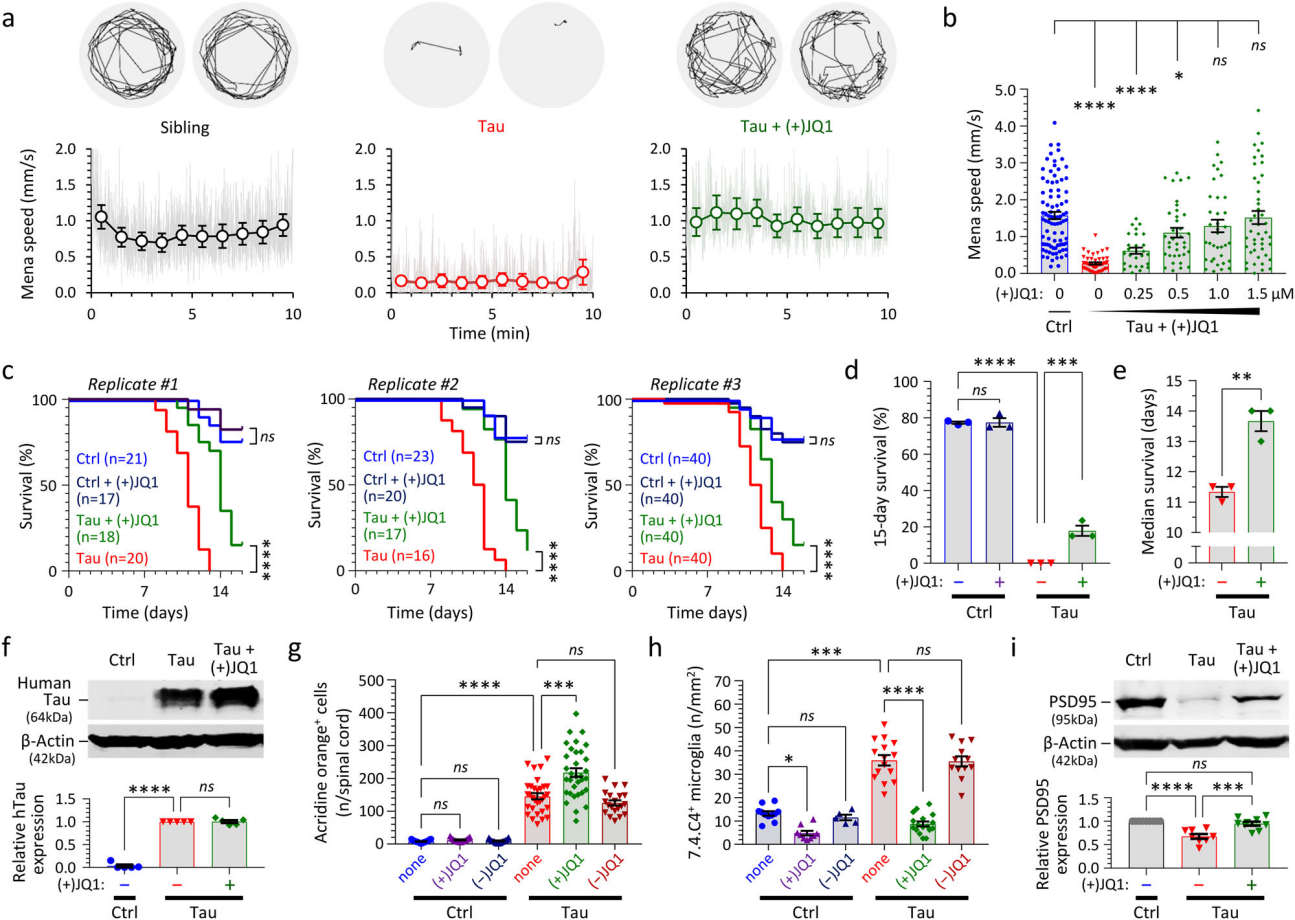
(+)JQ1 improves survival and rescues motor function, microgliosis, and expression of PSD95 in transgenic zebrafish expressing human 4R/0N-Tau. Zebrafish were exposed to repurchased (+)JQ1 or (−)JQ1 from 2dpf. **a** VMR light phase swimming speed at 5dpf. Example 1-min vectors are shown above. The graphs below show mean group frame-to-frame displacement scaled to speed (gray) and 1-min binned group mean ± SE (colored markers), similar to Fig. 5b. **b** Relationship between mean speed during the light phase of the VMR at 5dpf and (+)JQ1 concentration; data points show individual zebrafish (*n* = 27–88 zebrafish/group, combined from 4 experimental replicates), bars show mean ± SE. ****p < 0.0001, *p < 0.05, 1-way ANOVA with Dunnett’s multiple comparisons test (Supplementary Table 18). **c** Zebrafish survival in three biological replicate cohorts. Group sizes are indicated in the graph legends. ****p < 0.0001, *ns* not significant, Mantel–Cox test (Supplementary Tables 19, 20). **d** 15-day % survival of the three cohorts shown in **c**. Bars show mean ± SE; ****p = 6.9 ×10^−9^, ***p = 0.00049, 1-way ANOVA with Šidák multiple comparisons test. **e** Median survival of [Tau] and [Tau + (+)JQ1] zebrafish from three cohorts shown in **c**. Bars show mean ± SE; **p = 0.0087, 2-tailed unpaired *t*-test. **f** Western blot showing expression of human Tau (above) and β-Actin (below) at 5dpf. Quantification of mean ± SE relative Tau expression in 5 biological replicate experiments is shown in the accompanying graph. ****p = 5.7 × 10^−12^, 1-way ANOVA with Dunnett’s multiple comparisons test. **g** Acridine Orange labeled spinal cord cells quantified as shown in Fig. 2a, d. Data points show individual zebrafish (*n* = 15–24 zebrafish/group), bars show mean ± SE. ****p < 10^−15^, ***p = 1.4 ×10^−8^, 1-way ANOVA with Šidák multiple comparisons test. **h** Microglia quantified at 5dpf, as shown in Fig. 2h, i. Data points show mean values for 10–12 sections from individual zebrafish (*n* = 5–17 zebrafish/group), bars show group mean ± SE. ****p < 10^−15^, ***p = 9.5 × 10^−14^, *p = 0.010, 1-way ANOVA with Šidák multiple comparisons test. **i** Western blot showing expression of PSD95 (above) and β-Actin (below) at 5dpf. Quantification of mean ± SE relative PSD95 expression in 8 biological replicate experiments is shown in the accompanying graph. ****p = 0.000018, ***p = 0.00035, 1-way ANOVA with Tukey multiple comparisons test. Source data are provided as a Source Data file.

(+)JQ1 rescued hypokinesia in Tau zebrafish in a concentration-dependent manner (Fig. 8a, b, Supplementary Fig. S24, Supplementary Table 18). Exposure to the inactive stereoisomer (−)JQ1 at the same concentrations did not improve motor function in Tau zebrafish (Supplementary Fig. 25a), demonstrating the requirement of a specific ligand-bromodomain interaction for activity in this assay. (+)JQ1 did not increase the swimming speed of Ctrl zebrafish (Supplementary Fig. S25b), excluding non-specific stimulation of zebrafish motility as the explanation for the observed rescue. Exposure of Tau zebrafish to (+)JQ1 did not silence transgene expression, since mCherry expression was quantitatively similar throughout the CNS of (+)JQ1-exposed and untreated Tau zebrafish (Supplementary Fig. S26). Together these essential controls suggest that (+)JQ1 rescues neurological phenotypes in Tau zebrafish by modulating BET bromodomain function.

(+)JQ1 increased the median survival of Tau zebrafish by 15–20% but did not alter the survival of Ctrl zebrafish (Fig. 8c–e; Supplementary Tables 18–20). (+)JQ1 did not reduce 0N/4R-Tau levels or alter Tau phosphorylation (determined by electrophoretic mobility and immunoreactivity to phosphoepitope-specific antibodies) in Tau zebrafish (Fig. 8f), compatible with a mechanism of action conceptually downstream of Tau. Interestingly, (+)JQ1 did not decrease acridine orange labeled cells (Fig. 8g), suggesting that hypokinesia in this model is not caused by spinal cord cell death. However, (+)JQ1, but not (−)JQ1, decreased CNS microglial abundance substantially in Tau zebrafish at 5 dpf (Fig. 8h; the reduced density of microglia in (+)JQ1-exposed Tau zebrafish may delay phagocytic removal of apoptotic neurons, potentially accounting for the modest increase in AO-labeled cells seen in Fig. 8g). Western blot analysis showed that exposure to (+)JQ1 restored expression of the post-synaptic marker PSD95 in Tau zebrafish (Fig. 8i). Intriguingly, several components of the Tau zebrafish phenotype, including decreased motor activity in the dark phase of the VMR and disrupted OKRs, were not rescued significantly by (+)JQ1 (Supplementary Fig. S27). It is possible these phenotypes are mediated by distinct mechanisms that are not targeted by this compound.

### (+)JQ1 inhibits microglial phagocytosis of synaptic material in both Tau transgenic zebrafish and a rat primary cortical culture model expressing human 4R/0N-Tau

**Fig. 9:**
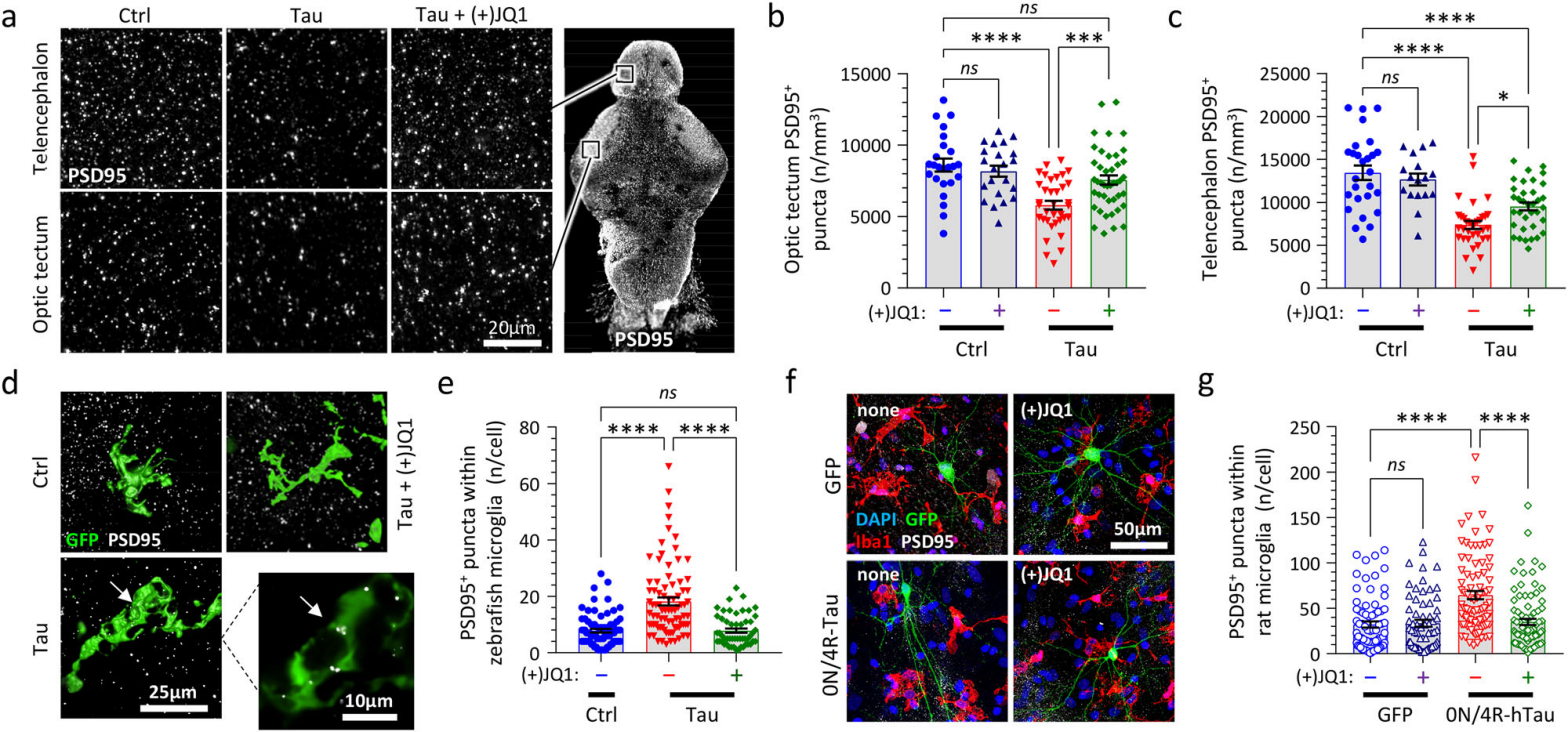
(+)JQ1 inhibits microglial synaptic elimination in both transgenic zebrafish and rat primary culture models expressing human 4R/0N-Tau. **a** Whole mount immunofluorescence for PSD95-immunoreactive post-synaptic puncta in the telencephalon (above) and optic tectum (below; approximate regions of images shown in low-magnification panel to right). Labeled brains were imaged by confocal microscopy. Maximum intensity projections are shown. PSD95-immunoreactive post-synaptic puncta were quantified in tissue volumes from (**b**) optic tectum and (**c**) telencephalon using unbiased automated image analysis algorithms. Data points show individual zebrafish (*n* = 17–43 zebrafish/group), bars show mean ± SE. ****p < 0.0001, ***p < 0.001, *p < 0.05, 1-way ANOVA with Šidák multiple comparisons test. **d** Fixed whole mount brains from [Tau or Ctrl] x Tg(*mpeg1:egfp*) zebrafish immunolabeled for GFP and PSD95 at 4dpf and imaged by confocal microscopy. Alpha shaded 3D projections of microglia are shown. The inset shows a single confocal plane from a Tau zebrafish, illustrating PSD95-immunoreactive synaptic material inside the cytoplasm of the microglial cell (arrow). **e** PSD95^+^ puncta quantified inside microglia in Ctrl, Tau, and [Tau + (+)JQ1] zebrafish as shown in panel **d**. Data points show individual microglia (*n* = 52–83 microglia from 6 zebrafish/group), bars show group mean ± SE. ****p < 0.0001, 1-way ANOVA with Tukey multiple comparisons test. **f** Primary embryonic rat cortex cultures were established under conditions promoting microglial survival and differentiation. Neurons were transfected to express human 0N/4R-Tau and membrane-anchored GFP (mGFP), or mGFP alone. Cultures were exposed to 1 μM (+)JQ1 (or vehicle only) and labeled for Iba1 (microglia; red), mGFP (transfected neurons; green), PSD95 (synaptic puncta; white), and DAPI (nuclei; blue). **g** PSD95^+^ puncta were quantified inside microglia (Supplementary Fig. S32) from each of the primary rat cortex cultures. Data points show individual microglia (*n* = 65–87 microglia from 3 biological replicates per group), bars show group mean ± SE. ****p < 0.0001, 1-way ANOVA with Šidák multiple comparisons test. Source data are provided as a Source Data file. Exact *p*-values shown in Supplementary Table 21.

Since (+)JQ1 prevents microgliosis and restores PSD95 expression in Tau zebrafish, we next asked whether rescue of motor function might be explained by (+)JQ1 preventing microglial elimination of synapses. Microglial synaptic pruning was identified recently as an important mechanism in neurodegenerative disease models^43^ and synaptic loss was previously demonstrated histopathologically in PSP^44,45^. Volumetric imaging of whole mount brains, coupled with unbiased automated image analysis (Fig. 9a; Supplementary Fig. S28), showed that the density of PSD-95 immunoreactive synaptic puncta in Tau zebrafish was decreased in both the optic tectum (Fig. 9b) and dorsal telencephalon (Fig. 9c) compared with Ctrl. This deficit in synaptic abundance was rescued by exposure to (+)JQ1 (Fig. 9b, c, Supplementary Table 21). To test whether these findings might be attributable to microglial elimination of synapses, whole mount zebrafish brains were co-labeled for microglial and synaptic markers. PSD95-immunoreactive puncta were detected within the cytoplasm of zebrafish microglia, indicating phagocytosis of synaptic material (Fig. 9d; Supplementary Fig. S29). The frequency of PSD95 puncta within individual microglia was increased in Tau zebrafish compared with controls, and this increase was prevented by (+)JQ1 exposure (Fig. 9e; Supplementary Fig. S30, Supplementary Table 21). These data together suggest that (+)JQ1 inhibits microglial phagocytosis of synapses, potentially contributing to its activity in rescuing motor function and survival of Tau zebrafish.

To determine whether (+)JQ1-mediated inhibition of microglial synapse removal is conserved in a mammalian model, we adapted a well-established embryonic rat cortex primary culture system^46^ to yield neuron-astrocyte-microglia co-cultures^47^ (Fig. 9f; Supplementary Fig. S31). Similar to Tau zebrafish, PSD95-immunoreactive puncta were detected within cultured rat microglia, indicating phagocytosis of synaptic material (Supplementary Fig. S32). Transfection of neurons in the co-culture to express human 4R/0N-Tau and a membrane-anchored GFP (mGFP) marker substantially increased the abundance of intra-microglial PSD95 puncta, compared with controls in which neurons were transfected to express the mGFP reporter only (Fig. 9g; Supplementary Fig. S33). Importantly, the increase in microglial PSD95 puncta caused by neuronal 0N/4R-Tau expression was prevented by (+)JQ1 exposure (Fig. 9g; Supplementary Fig. S33, Supplementary Table 21).

These data together suggest that neuronal 0N/4R-Tau accumulation promotes microglial phagocytosis of synaptic material in both zebrafish and rat models, through a conserved mechanism that is inhibited by (+)JQ1.

### Loss of Brd4 phenocopies the effects of (+)JQ1 in Tau zebrafish

**Fig. 10:**
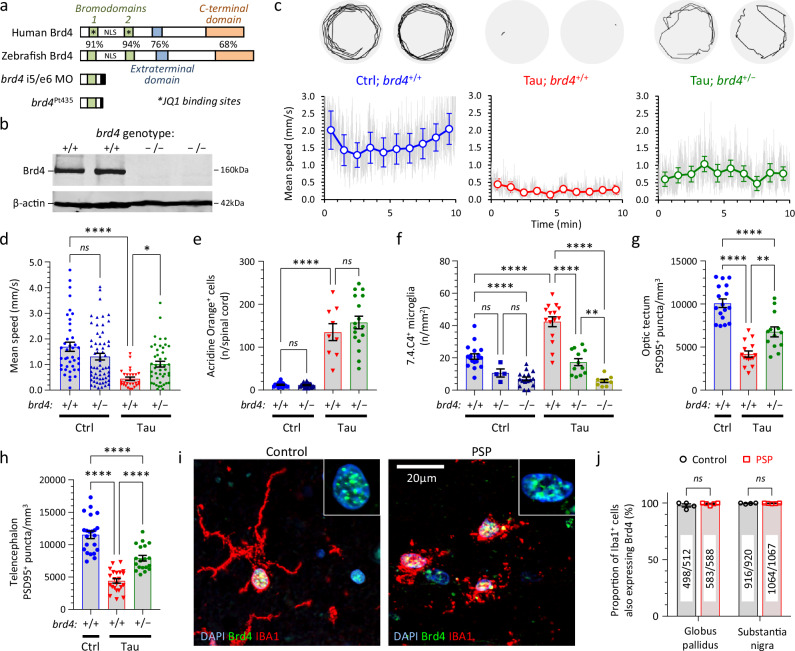
Brd4 regulates microgliosis and synaptic pruning in transgenic zebrafish expressing human 0N/4R-Tau and is expressed in human microglia. **a** Schematic showing key functional domains of the human and zebrafish Brd4 orthologues. Amino acid homology is indicated for each domain. Truncated products formed by a splice site-targeting morpholino, or a stable germline 11-bp deletion in exon 4 of *brd4* (pt435) are shown. **b** Western blot showing expression of Brd4 (above) and β-actin (below) in brains from WT and *brd4*^Pt435/Pt435^ (abbreviated to *brd4*^−/−^) zebrafish. **c**, **d** VMR light phase speed of [Ctrl], [Tau], and [Tau; *brd4*^+/−^] zebrafish. **c** Example 1-min swim vectors (above); mean speed during the light phase of the VMR (below). Gray traces show mean frame-to-frame displacement scaled to show speed, colored markers and bars show 1-min binned group mean ± SE, similar to Fig. 5b. **d** Mean speed of individual zebrafish (*n* = 28–62 zebrafish/group), bars show group mean ± SE. ****p = 2.9 × 10^−7^, *p = 0.029, 1-way ANOVA with Šidák multiple comparisons test. **e** Acridine orange labeled spinal cord cells quantified as shown in Fig. 2a, d. Data points show individual zebrafish (*n* = 9–24 zebrafish/group), bars show group mean ± SE. ****p = 3.2 × 10^−11^, 1-way ANOVA with Šidák multiple comparisons test. **f** Microglial density quantified as shown in Fig. 2h, i. Data points show individual zebrafish (*n* = 4–18 zebrafish/group), bars show mean ± SE. ****p < 0.0001, **p < 0.01, 1-way ANOVA with Šidák multiple comparisons test. **g**, **h** PSD95-immunoreactive synaptic puncta quantified in (**g**) optic tectum or (**h**) telencephalon. Data points show individual zebrafish (*n* = 13–22 zebrafish/group), bars show mean ± SE. ****p < 0.0001, 1-way ANOVA with Tukey multiple comparisons test. **i** Sections from control and PSP human substantia nigra labeled for Brd4 (green), Iba1 (microglia; red) and nuclei (DAPI, blue), and imaged by confocal microscopy. Inset panels show Brd4-immunoreactive microglial nuclei from the main panels at higher magnification. **j** The proportion of Iba1^+^ cells showing Brd4-immunoreactive nuclei in multiple sections of substantia nigra and globus pallidus from 4 control and 5 PSP cases. Data points show individual cases, bars show group mean ± SE, comparison by unpaired 2-tailed *t*-test. Numbers of [Brd4^+^, Iba1^+^]/[Iba1^+^] microglia analyzed in each group are indicated. Source data are provided as a Source Data file. Exact *p*-values for panels (**f**–**h**) shown in Supplementary Table 22.

We next used reverse genetics to identify the molecular target mediating the activity of (+)JQ1 in the zebrafish Tau model. Previous work showed that (+)JQ1 binds with high affinity to the BET protein Brd4^48^, which has been implicated in inflammatory signaling in other contexts^49^. Zebrafish have a single highly-conserved orthologue of human Brd4^50^. Antisense morpholino oligonucleotides (MO) that targeted splicing or translation of zebrafish *brd4* mRNA decreased Brd4 expression and mitigated microgliosis in Tau zebrafish at 3–4 dpf (Fig. 10a; Supplementary Fig. S34). However, progressive loss of MO during cell division following microinjection leads to recovery of gene knockdown by 2–3 dpf^18^. In order to test the role of Brd4 in Tau zebrafish at later timepoints when phenotypic abnormalities are most prominent, we engineered a stable 11-bp deletion in exon 4 of *brd4* that eliminated Brd4 expression, using Cas9/CRISPR (allele designation pt435; Fig. 10a, b; Supplementary Fig. S35). Homozygous *brd4*^−/−^ mutants lacking Brd4 showed morphological and motor deficits, and were not viable (Supplementary Figs. S36, S37). However, heterozygous *brd4*^+/−^ zebrafish showed no overt abnormalities, and [Ctrl; *brd4*^+/−^] zebrafish showed similar motor function to their [Ctrl; *brd4*^+/+^] siblings (Fig. 10d), allowing us to test the effect of the heterozygous *brd4* mutation on the neurological phenotypes of Tau zebrafish. The VMR light phase mean speed of [Tau; *brd4*^+/−^] zebrafish was significantly higher than their [Tau; *brd4*^+/+^] siblings, demonstrating that decreased Brd4 expression partially rescued hypokinesia in Tau zebrafish (Fig. 10c, d; Supplementary Fig. S38, Supplementary Table 22). Similar to (+)JQ1 exposure, reduced Brd4 expression did not rescue acridine orange labeled spinal cord cells (Fig. 10e, Supplementary Table 22) or OKR deficits (Supplementary Fig. S39) in Tau zebrafish. However, there was a robust decrease in CNS microglial abundance in Tau zebrafish harboring *brd4* mutations (Fig. 10f, Supplementary Table 22). The density of brain microglia was highest in *brd4*^+/+^ zebrafish, decreased in heterozygous *brd4*^+/−^ mutants, and lowest in homozygous *brd4*^−/−^ mutants. These data suggest that Brd4 regulates microglial activation, proliferation, migration, or viability in tauopathy, in an expression level-dependent manner. Together with resolution of microgliosis, Tau zebrafish harboring a heterozygous *brd4*^+/−^ mutation also showed rescue of synaptic abundance in the optic tectum and dorsal telencephalon (Fig. 10g, h; Supplementary Fig. S40, Supplementary Table 22). Mutation of the *brd4* gene was thus sufficient to phenocopy the key features of (+)JQ1-mediated rescue in Tau zebrafish, compatible with (+)JQ1 acting by inhibiting Brd4 in this model. Although additional binding activities of (+)JQ1 are not necessary to explain the findings, the data do not formally exclude contributions to phenotypic rescue from interactions between (+)JQ1 and the bromodomains of other proteins such as Brd2 or Brd3^48^.

### Brd4 expression in human microglia

Finally, we examined Brd4 expression in control and PSP human brains. Prior work showed that BRD4 mRNA is expressed in neurons and glia throughout the human brain, and is enriched in microglia compared with other glial cell types^51^. In view of our finding that Brd4 plays an important role in microgliosis and microglial synapse elimination in Tau zebrafish, we labeled human autopsy brain sections from 4 controls and 5 PSP cases with antibodies to Brd4 and Iba1 (a microglial marker) and examined expression in the substantia nigra and globus pallidus, two areas affected prominently by PSP pathology^1,5^. Strong punctate nuclear Brd4 immunoreactivity was found in both quiescent (highly ramified) microglia in control brains, and in activated (retracted processes) microglia in PSP brains (Fig. 10i; Supplementary Fig. S41). This pattern of nuclear Brd4 expression was found in 1414/1432 microglia examined in control brains, and 1648/1664 microglia in PSP brains (Fig. 10j; Supplementary Fig. S42, Supplementary Table 22). In addition, nuclear labeling was also observed in many Iba1^−^ (non-microglial) cells. There was no obvious difference in Brd4 labeling intensity or pattern between control and PSP brains, although critical functions of Brd4 in the regulation of gene expression have previously been shown to occur in the absence of changes in its expression level^52^.

## Discussion

Development of a zebrafish PSP model optimized for unbiased phenotype-driven chemical biology screening is a significant advance. By generating UAS responder zebrafish in the absence of Gal4, we avoided negative selection against larvae with impaired viability resulting from human 4R-Tau expression. Subsequent selection for strong transactivation by a pan-neuronal Gal4 driver yielded lines expressing Hsa.MAPT transgene RNA at approximately 12-fold higher levels than endogenous *mapta* and *maptb* (quantification of protein expression awaits the availability of suitable antibodies). Over-expression of total^53^ and exon 10-containing (4-repeat)^9^ MAPT mRNA in subjects homozygous for the PSP risk-associated MAPT H1 haplotype is modest and insufficient alone to cause PSP, suggesting that other factors in addition to Tau expression levels contribute to the initiation of pathogenesis. Consequently, transgenic over-expression models likely do not replicate upstream events in human tauopathies. However, these models provide an accessible experimental system that is appropriate for defining pathophysiological mechanisms downstream of Tau accumulation. For example, the PS19 mouse model expresses P301S mutant human 1N/4R-Tau protein at approximately 5-fold higher levels than endogenous murine Tau, develops pathological and clinical abnormalities reflective of human tauopathy over a time course of months, and has provided numerous key insights into the pathogenesis of disease^54–56^. Similarly, Tau zebrafish show reproducible PSP-relevant changes, at larval timepoints when screening and other analyses are practicable. Importantly, Tau zebrafish have construct validity as a PSP model, since their phenotypes are caused by the same WT human 0N/4R-Tau isoform that accumulates most prominently in PSP^6,7^. The model also has strong face validity as it replicates many of the core clinical (hypokinesia, oculomotor deficits, impaired survival), pathological (neurodegeneration, neuroinflammation, synapse loss) and biochemical (Tau hyperphosphorylation, mislocalization, misfolding, truncation, insolubility, and oligomerization) features of PSP.

We showed proof of concept that phenotypic screening in a zebrafish PSP model can identify small molecules that yield insights into the pathophysiology of tauopathy. The use of PSP-relevant neurological deficits as endpoints – made possible by the robust and rapid phenotype of the model, coupled with optimized assay workflows^36^ – allows analysis of small molecules without preconceptions about their mechanism of action in vivo. Screening is therefore unbiased with respect to the neurobiological mechanisms by which target engagement modulates phenotypic outcomes. Microglial synaptic elimination in tauopathy is a cell non-autonomous mechanism that requires the presence of microglia and end-differentiated neurons with synaptic connections, and presumably drives the appearance of neurological phenotypes by disrupting the physiology of neural circuits. This level of complexity would be difficult to replicate in vitro, and our unanticipated discovery that Brd4 regulates this mechanism highlights the value of screening in vivo. A separate bias relates to the contents of small molecule libraries, which are frequently chosen to engage a focused range of targets. Library selection for our initial screen was based partly on the relatively unexplored role of epigenetics in tauopathy, as we anticipated this would enhance the value of the study by increasing the likelihood of discovering novel modifiers. The modest library size further allowed us to address the multiple practical considerations involved in establishing a screen, including zebrafish breeding, chemical exposure, assay workflow, and data analysis. It is important to recognize that we surveyed a limited set of molecular targets in the present study as a result of this library selection. However, our work validates the zebrafish model and screening approach, allowing pursuit of more ambitious projects involving larger and more diverse small molecule libraries in future work.

Microglial synaptic elimination strongly influenced motor function and survival in Tau zebrafish, underlining its significance as a mechanism mediating phenotypic outcomes in this model. Recent work showed that synaptic loss in mouse Alzheimer disease models involves deposition of complement C1q and C3 at synapses, and complement-dependent microglial engulfment, activated via C3R receptors^43,57,58^. Complement was also involved in mouse tauopathy models, in which synaptic loss was rescued by C1q antibodies^59^ or loss of C3aR1 receptors^55^, and negatively regulated by Nptx2, a secreted neuronal pentraxin that binds C1q^60^. Brd4 has not been implicated in pathological synapse elimination previously, and it is currently unclear how it might regulate this process. A BET inhibitor was recently reported to downregulate basal and cytokine-simulated expression of complement components in cultured hepatocytes and humanized mice, and to reduce activated complement C3 and C5 levels in the circulation of cardiovascular disease patients^61^. However, it is currently unknown whether Brd4 regulates complement in the CNS and our observations might be attributable to alternative or indirect mechanisms, such as roles of Brd4 in microglial proliferation, migration, activation, or proinflammatory signaling^49,62–64^. Importantly, Tau zebrafish provide a tractable experimental system to address these possibilities systematically, facilitated by the extensive phylogenetic conservation of complement^65^ and microglial transcriptional programs^66^ between zebrafish and mammals, and by the ability to monitor microglial behavior directly in transgenic zebrafish by intravital microscopy^67^. As Brd4 is expressed in multiple CNS cell types, additional work will also be necessary to establish whether the critical role of Brd4 in microglial synapse elimination depends on a cell-autonomous function in microglia, or a Brd4-dependent signal from another cell type.

We did not observe obvious changes in Brd4 expression pattern or level by immunofluorescence in human PSP substantia nigra or globus pallidus compared with control. These findings are compatible with published studies. Bulk RNAseq analysis of human superior temporal gyrus showed a modest 20–30% upregulation of BRD4 mRNA in PSP subjects, and snRNAseq analysis in the same study suggested BRD4 upregulation was more prominent in glia than neurons, but none of these changes was statistically significant after adjustment for multiple comparisons^68^. However, previous work showed that Brd4 mediates key regulatory events in innate immune cells in the absence of alterations in its expression level^52^. Murine bone marrow macrophages exposed to LPS, a potent inflammatory stimulus, showed multiple Brd4-dependent gene expression changes accompanied by widespread binding of Brd4 to chromatin near the regulatory elements of LPS-stimulated genes^52^. This occurred in the presence of stable Brd4 levels^52^ suggesting that changes in Brd4 expression are unnecessary for it to drive profound alterations in cellular transcriptional programs. It will be of interest in future studies to determine how neuronal Tau accumulation influences sites of Brd4 chromatin occupancy, and any resulting changes in Brd4-dependent gene expression, in both neurons and glia.

The improved survival and neurological function we observed in Tau zebrafish exposed to (+)JQ1 raises several questions about the translational potential of bromodomain inhibitors in PSP, especially as compounds targeting BET bromodomains have already been tested in clinical studies for other indications (although with associated toxicity)^69,70^. Our findings that (+)JQ1-mediated mitigation of microglial synaptic phagocytosis is phylogenetically conserved between Tau zebrafish and a rat primary culture model, and that Brd4 is expressed in human microglia in PSP, are together encouraging that Brd4 may regulate microglial synaptic pruning in PSP in a pharmacologically-targetable way. Prior work supports the premise that bromodomain inhibitors can ameliorate neuroinflammation^63,64,71^. For example, (+)JQ1 reduced inflammatory marker expression in the CNS of a mouse Alzheimer’s disease model, but did not rescue neurological phenotypes^71^, possibly owing to its short half-life (0.9 h)^63^ coupled with intermittent administration (this is not a concern in zebrafish, which are bathed continuously in a large volumetric excess of (+)JQ1 solution). However, not all phenotypes we observed in Tau zebrafish were rescued by either (+)JQ1 or decreased Brd4 expression. While this may reflect the timing or potency of the experimental manipulations relative to pathogenesis in the model, these data also raise the intriguing possibility that specific components of the full phenotypic profile are driven by distinct molecular and cellular mechanisms. Functional imaging studies in patients provide evidence of microglial activation^72^ and synaptic loss^73^ during PSP pathogenesis, but it is not yet clear how these contribute to the progression of neurological deficits in PSP. It is also noteworthy that (+)JQ1 has shown potentially detrimental effects in other experimental models of neurological disease, including worsening neurological phenotypes in a mouse model of Huntington’s disease^74^, and a modest increase in Tau phosphorylation in cultured human neural cells harboring multiple APP and PSEN1 mutations^75^. The degree to which these observations reflect divergent models or experimental systems, pleiotropic functions of BET proteins, or the relatively low bromodomain selectivity of available tool compounds is unclear. However, it will be important to address these questions before advancing Brd4 as a pharmacological target in PSP.

Our screen identified several additional pathways that may contribute to 4R-tauopathy pathogenesis in vivo. Histone acetylation^76^ and HIF-1α signaling^77^ were implicated previously in other neurodegenerative diseases, but have not yet been investigated in PSP. Prior work showing DNA hypermethylation at multiple gene loci in PSP brains^78^ is compatible with our finding that DNA methyltransferase inhibitors as a group overall improved phenotypes in Tau zebrafish. Together, these observations provide preliminary evidence that Tau zebrafish may show predictive validity as a PSP model and suggest additional directions for future investigation. Given the rich yield from the initial screen, we are currently scaling the approach to interrogate larger libraries. We also anticipate that the zebrafish PSP model (together with the open-source software and methods we developed for its automated quantitative analysis^35,37,40^) will be useful for multiple additional downstream applications, including optimizing experimental PSP therapeutics rapidly in vivo; hypothesis-driven analysis of pathophysiological mechanisms; evaluation of PSP risk alleles using reverse genetics; and genetic verification of targets for small molecule screening ‘hits’.

## Methods

### Ethical approvals

All studies involving vertebrate animals were carried out with approval from the University of Pittsburgh Institutional Animal Care and Use Committee (IACUC), and in accordance with the NIH *Guide for the Care and Use of Laboratory Animals* (IACUC protocol number for zebrafish studies 23012444; IACUC protocol number for rat studies 24034765; University of Pittsburgh PHS Assurance Number D16-00118).

All studies involving human tissue were carried out with approval from the University of Pittsburgh Committee for Oversight of Research and Clinical Training Involving Decedents (CORID; protocol number 793). Permission to collect and analyze tissue for research was granted by next-of-kin at the time of autopsy.

### Zebrafish

Adult zebrafish were housed in 9 L tanks, in continuously circulating clean system water at 25 °C, under cyclic illumination by ceiling-mounted fluorescent white lights (70 lx) with 14 h light – 10 h dark (light on at 08:00). Zebrafish embryos were generated by crossing adults in breeding tanks and raised at 28.5 °C in E3 buffer (5 mM NaCl, 0.17 mM KCl, 0.33 mM CaCl_2_, 0.33 mM MgSO_4_; Sigma, St. Louis, MO) in 10 cm dishes, under cyclic illumination by incubator-mounted white LEDs (200 lx) with 14 h of light – 10 h of dark (light on at 08:00). Larval zebrafish <15 days post-fertilization were analyzed in the experiments shown; zebrafish sex is not established at this developmental point, and so the study included subjects with potential to differentiate into either male or female zebrafish. During colony management, adult zebrafish were euthanized according to NIH guidelines, by immersion in buffered tricaine 300 mg/L until 30 min after all opercular movements ceased. After completion of experiments, larval zebrafish <15dpf were euthanized according to NIH guidelines, by immersion in buffered tricaine 300 mg/L until all responses were abolished, followed by 5% sodium hypochlorite.

### DNA constructs

A p2A-nls DNA fragment was amplified from plasmid T2M-mCherry-2A-nls-eGFP (a gift from Dr. Steven Leach, Dartmouth College)^26^ using primers 5′-CGGGATCCGGAGCCACGAAC-3′ and 5′-CCATGTTATCCTCCTCGCCCTTGCTCAC-3′, digested with *Bam*HI, and inserted into the *Bam*HI/*Msc*I sites of pmCherry (Clontech, Palo Alto, CA) to make p2A-nls-mCherry. A p2A-nls-mCherry fragment was then amplified from p2A-nls-mCherry using primers 5′-GTGATATCCGGAGCCACGAACTTC-3′ and 5′-CCGCTCGAGCCGCTACTTGTACAGC-3′, digested with *Eco*RV and *Xho*I and inserted into pT2MUASMCS (a gift from Dr. Koichi Kawakami, National Institute of Genetics, Japan)^79^ to make pTol2-5UAS:p2A-nls-mCherry. The open reading frame of human 4R/0N-Tau was amplified from plasmid t2 (a gift from Dr. Matthew Farrer, University of Florida)^23^ using primers 5′-GAAGATCTGTCGACGAATTCCC-3′ and 5′-CCCAAACCCTGCTTGGCCAG-3′, digested with *Bgl*II and inserted into the *Bgl*II/*Eco*RV sites of pTol2-5UAS:p2A-nls-mCherry to make pTol2-5UAS:Hsa.MAPT-p2A-nls-mCherry. All DNA constructs were sequenced fully, and expression of mCherry was verified in transient co-transfection experiments prior to generating transgenic lines.

To generate mammalian cell culture expression plasmids, a DNA fragment encoding the CAAX membrane anchoring signal was inserted into the *Bsr*GI/*Not*I sites of pIRES2-GFP (Clontech; #2069-1) to generate pIRES2-mGFP. A human cDNA fragment encoding 0N/4R-Tau was amplified with primers 5′-GGAATTCATGGCTGAGCCCCGCC-3′ and 5′-AAGGCCTCACAAACCCTGCTTGG-3′ and inserted into the *Eco*RI/*Sma*I sites of pIRES2-mGFP to make pIRES2-Tau-mGFP. All plasmids were verified by restriction mapping and sequencing, and the expression of Tau and mGFP was confirmed by Western blotting and microscopy after transient transfection.

### Transgenic zebrafish

Stable transgenic zebrafish lines were generated exactly as described in our previous work^29,80^, by microinjecting pTol2-5UAS:p2A-nls-mCherry or pTol2-5UAS:Hsa.MAPT-p2A-nls-mCherry, together with mRNA encoding Tol2 transposase, into single-cell embryos from WT strain AB zebrafish. For pTol2-5UAS:Hsa.MAPT-p2A-nls-mCherry, we identified 10 different F0 chimeras by PCR of pooled F1 embryo DNA resulting from a WT outcross, using primers 5′-AGATCTGTCGACGAATTCCC-3′ and 5′-AATCCTGGTGGCGTTGGCCT-3′. mCherry expression was then evaluated in the F1 progeny of each F0 chimera, after crossing with a Tg(*eno2:Gal4FF*) neuronal driver line^81^. Four of the crosses generated progeny with bright fluorescence; the F0 parent in each cross was then crossed again with WT zebrafish, and the progeny raised to adulthood. F1 founders for each Tg(*UAS:Hsa.MAPT-p2A-nls-mCherry*) line were identified by PCR of fin clip DNA from adults. mCherry expression was verified in F2 progeny of F1 x Tg(*eno2:Gal4FF*) crosses. Lines were then derived from single F1 founders. Derivation, expansion, and maintenance of lines was carried out by WT outcross in the absence of a Gal4 driver, using fin clip PCR genotyping.

Of several Gal4 pan-neuronal driver lines tested, Tg(*elavl3:gal4-vp16*)^27^ yielded the strongest transactivation of the UAS alleles and was used for subsequent experiments. Identical phenotypes were observed in multiple different 4R-Tau responder alleles when crossed to the pan-neuronal driver. Line Tg(*UAS:Hsa.MAPT-p2A-nls-mCherry*)^pt433^ showed stable expression and minimal variegation over multiple generations (currently F9) and was used for the studies shown here. Tg(*UAS:p2A-nls-mCherry*)^Pt434^ controls were generated similarly but can be maintained on a Gal4 driver background as they have no phenotype other than neuronal mCherry fluorescence.

Other control lines Tg(*UAS:egfp*)^82^ and Tg(*UAS:Hsa.SNCA-p2A-nls-mCherry*)^29^ were reported in our previous work. Tg(*mpeg1:egfp*) zebrafish^83^ were crossed with Tau and Ctrl lines to express GFP throughout the cytoplasm of microglia for whole mount microscopy studies.

### Quantitative real-time RT-PCR

Total RNA was extracted from zebrafish larvae 3–6 dpf by column purification (RNAqueous; Thermo Fisher; #AM1912), verified by gel electrophoresis and spectrophotometry, and first-strand cDNA synthesized by oligo-dT priming (SuperScript III; Thermo Fisher; #18080051). Each PCR reaction included 0.5 μL cDNA (or ddH_2_O in negative controls), 1 μL gene-specific primer pair (0.1 μM each primer; sequences are shown in Supplementary Table 1), 10 μL iTaq Universal SYBR Green Supermix (BioRad, Hercules, CA; #1725120) and ddH_2_O to a final volume of 20 μL. Real-time PCR was carried out using a BioRad CFX Opus 96 system. After hot start, samples underwent amplification through 40 cycles of [95 °C, 15 s – 72 °C, 30 s], followed by melt curve analysis. Every reaction was completed in technical triplicate and all experiments involved multiple biological replicate samples. Quantitative analysis of gene expression was carried out using CFX Maestro software version 2.3.

### Brd4 knockdown with morpholino oligonucleotides

Custom morpholino oligonucleotides (MO; Gene Tools, Philomath, OR) were designed to target the translational start site of *brd4* mRNA 5′-CGTCCAGGCCGTCCCCCATACTAG-3′ or the slice acceptor at the intron 5/exon 6 boundary in the primary *brd4* transcript 5′-TCATGTCTAATGACACAGAAAGAGA-3′. MO stocks were diluted to a working concentration of 5 ng/nL in Danieau microinjection buffer (8 mM NaCl, 0.7 mM KCl, 0.4 mM MgSO_4_, 0.6 mM Ca(NO_3_)_2_, 5 mM HEPES, pH 7.6) with 0.5% phenol red. 1 to 2 nL of MO solution was injected into the yolk sac of each embryo at 1–4 cell stage. A standard non-targeting MO 5′-CCTCTTACCTCAGTTACAATTTATA-3′ (cat # PCO-StandardControl-100; Gene Tools) was used as a negative control at the same concentration and volume. Microinjected embryos were allowed to develop to 2–4 dpf then lysed in RIPA for Western blot (see below) or fixed for immunohistochemistry to quantify microglia.

### Brd4 knockout zebrafish

A custom gRNA 5′-GCCGGGGCAGGAGGGAUCC-3′ was designed to target a *Bam*HI restriction site at the 3′ end of zebrafish *brd4* exon 5. The gRNA was synthesized by Sigma to include additional hybridization sequences for tracrRNA. Embryos were microinjected at the single cell stage with 2nL of a solution containing gRNA, tracrRNA (cat # TRACRRNA05N; Sigma), *S.pyogenes* Cas9 (cat # M0386T; New England Biolabs) and phenol red. Each 2nL injection solution contained 130 pg of gRNA. Microinjected embryos were raised to adulthood. F0 chimeras were identified by genotyping the offspring of a wild-type outcross for loss of the targeted *Bam*H1 site. Pooled genomic DNA was amplified using PCR primers 5′-CCTATGGACATGGGAACAATCAA-3′ and 5′-GGAACCCTATGCAGTTATCAAACTG-3′, yielding a 530 bp product spanning exon5 and intron 6 of *brd4*. *Bam*HI digest of the WT PCR product results in restriction fragments of 333 bp and 197 bp, whereas mutation affecting the *Bam*HI site results in an uncut 530 bp product after digest. Offspring of F0 chimeras crossed to WT zebrafish were grown to adulthood and fin clip DNA obtained for genotyping by PCR and *Bam*HI digest. This identified F1 founders, whose DNA was then sequenced to characterize mutations. Overall, we identified 5 unique F1 founders with different mutations (including deletions of 8 or 11 bp, insertion of 5 bp and combined deletion/insertions of 1/45 and 5/5 bp). We used the 11 bp deletion allele Pt435 for further studies, as it abolished Brd4 expression by western blot. The allele was outcrossed to WT zebrafish for 4 generations before being in-crossed, or crossed to Tau zebrafish, for analysis.

### Survival studies

Tg(*elavl3:gal4-vp16*); Tg(*UAS:Hsa.MAPT-2A-nls-mCherry*) ‘Tau’ zebrafish, were identified by mCherry expression at 3dpf. Siblings not expressing mCherry, unrelated WT zebrafish, and Tg(*elavl3:gal4-vp16*); Tg(*UAS:2A-nls-mCherry*) ‘Ctrl’ zebrafish were used as controls for comparison. Zebrafish were maintained under standard conditions for zebrafish care after 5 days post-fertilization (dpf), including fresh circulating water and feeding three times daily. Different experimental groups were maintained in separate tanks and the number of surviving zebrafish in each tank was counted daily until 15dpf. A similar approach was used to compare Tg(*elavl3:gal4-vp16*); Tg(*UAS:Hsa.SNCA-2A-nls-mCherry*) or Tg(*elavl3:gal4-vp16*); Tg(*UAS:2A-nls-mCherry*); Tg(*UAS:egfp*) zebrafish to non-expressing siblings.

### Cell death assays

For acridine orange labeling, 2–7 dpf Tau and Ctrl zebrafish were immersed in acridine orange (cat # A6014, Sigma, St. Louis, MO) 5 μg/mL in E3 embryo water in darkness for 30 min. After three washes in E3, zebrafish were embedded individually in 1.5% agarose and viewed using an Olympus CKX41 inverted epifluorescence microscope. The number of fluorescently labeled cells in each spinal cord was counted manually.

For terminal deoxynucleotidyl transferase dUTP nick end labeling (TUNEL) and Caspase 3 labeling, 2–7 dpf Tau and Ctrl zebrafish were fixed in 4% paraformaldehyde, washed in PBS, and cryoprotected in 30% sucrose. 12-μm-thick sections were collected in a sagittal plane using a cryostat. TUNEL labeling was carried out using a kit (TUNEL Andy Fluor 647 Apoptosis Kit, cat # A052, ABP Biosciences, Beltsville, MD), following the manufacturer’s protocol exactly. Cleaved (active) Caspase 3 was detected by immunohistochemistry as described below. Brain sections were imaged by transmitted light or widefield epifluorescence microscopy using an Olympus BX51 microscope. Labeled cells were quantified and normalized to tissue section area using ImageJ^84^, as shown in Supplementary Fig. S7.

### Zebrafish immunohistology

Zebrafish were fixed and sectioned as above. Sections were mounted on glass slides, washed with phosphate-buffered saline + 0.3% Triton X-100 (PBST) 3 times for 5 min each, incubated for 60 min in blocking buffer (1% bovine serum albumin in PBST), then incubated with primary antibody (see below) at 4 °C for 16 h. After washing with PBST, sections were incubated with secondary antibody for 60 min at room temperature. For chromogenic labeling, biotinylated secondary antibodies (1:200 in PBS; cat # BA9200; Vector Laboratories, Burlingame, CA) were used, followed by incubation with HRP-avidin-biotin complexes (Vectastain, Vector Laboratories, Burlingame, CA), chromogenic histochemical reaction with 3,3′Diaminobenzidine substrate (Vector Laboratories), and counterstaining in Mayer’s hematoxylin (Sigma). For immunofluorescence, fluorophore-conjugated secondary antibodies – goat anti-mouse IgG Alexa Fluor 488, goat anti-rabbit IgG Alexa Fluor 488, donkey anti-goat IgG Alexa Fluor 555 (Invitrogen) – were diluted 1:1000 in PBS with DAPI (final concentration 200 ng/mL in PBS; cat # 10236276001; Roche) as a nuclear counter-label.

Primary antibodies and dilutions for immunohistology were as follows. Figure 2c: purified rabbit anti-active Caspase 3 (1:200; cat # 559565, BD Biosciences, San Jose, CA). Figure 2h: mouse anti-zebrafish microglial marker clone 7.4.C4 (1:25; DSHB, University of Iowa)^85^. Figure 3e–g: mouse anti-human phospho(S202/T205)-Tau (AT8; 1:500, Cat# MN1020, Thermo Fisher), mouse anti-human Tau IgG1 (MC1; 1:100; a gift from Dr. Peter Davies, Albert Einstein Medical College), mouse anti-human Tau IgM (Alz50; 1:100; a gift from Dr. Peter Davies, Albert Einstein Medical College), mouse anti-human phospho(T231)-Tau (AT180; 1:250; Cat# MN1040, Thermo Fisher), mouse anti-human phospho(T181)-Tau (AT270; 1:500; Cat# MN1050, Thermo Fisher), mouse anti-human phospho(S396/S404)-Tau IgG1 (PHF1; 1:1000; a gift from Dr. Peter Davies, Albert Einstein College of Medicine), rabbit anti-human phospho(S422)-Tau (1:500; Cat# 44-764G, Thermo Fisher). Figure 4f: mouse anti-human truncated Tau (TauC3; 1:100; Cat# AHB0061, Invitrogen).

### Whole mount immunofluorescence

For whole mount synapse and microglia labeling shown in Fig. 9a, d and Supplementary Figs. S28 and S40, zebrafish at 4 or 5 dpf were fixed with 4% PFA, washed with PBS, dissected to expose the brain, treated with acetone for 8 min on ice, blocked in 10% normal goat serum/PBST at room temperature for 120 min, then incubated at 4 °C for 24 h with rabbit anti-PSD95 primary antibody (1:1000; Abcam; # ab18258), and chicken anti-GFP (1:5000; Abcam; # ab13970) for microglial labeling. After washing three times with PBS, samples were incubated with goat anti-rabbit IgG Alexa Fluor 488 secondary antibody (1:1000; Invitrogen; # A11008), or with goat anti-rabbit Alexa Fluor 647 (1:1000; Invitrogen; # A-21244,) and goat anti-chicken Alexa Fluor 488 (1:1000; Invitrogen; # A 11039) for microglial co-labeling, in 1% normal goat serum/PBST for 120 min at room temperature. After 3 washes in PBS for 5 min each, samples were embedded in low melting point agarose with the dorsal surface of the brain in contact with the cover slip of a 35 mm glass bottom dish (MatTek, Ashland, MA). Volumetric image stacks were acquired through the dorsal telencephalon and dorsolateral optic tectum, using a Nikon Ti2E inverted microscope with a 40x long working distance water immersion objective (CFI Apo LWD Lambda S 40XC WI NA 1.15) and Nikon AXR resonance scanning confocal system (Nikon USA, Melville, NY). Images were analyzed using NIS-Elements software version 5.30.05. Custom algorithms were developed using the General Analysis 3 module to quantify synaptic puncta, microglial volume, and synaptic puncta inside microglia, in each 3D image stack. To eliminate bias, all images for each experiment were acquired using identical settings and quantified using the same parameters and thresholds.

### Human brain immunohistology

Postmortem human tissue was obtained from the neurodegenerative disease brain bank in the Division of Neuropathology at the University of Pittsburgh. PSP cases (3 female, 2 male) satisfied published neuropathological diagnostic criteria^86^. Controls (1 female, 3 male) showed no evidence of neurodegenerative disease. Age at death did not differ significantly between PSP cases (77.2 ± 10.8) and controls (85.5 ± 5.8; p = 0.17, 2-tailed *t*-test). The brain was bisected at the time of autopsy and the left hemisphere was fixed in formalin, following which representative brain regions were embedded in paraffin and sectioned for diagnostic evaluation. For the present study, slides were incubated at 60 °C for 30 min, deparaffinized in xylene 3 times for 4 min each, passed through an ethanol series (100%, 95%, 70% twice for 2 min at each concentration) to water, then treated with 0.1% (in 70% EtOH) Sudan Black (Cat # 199664; Sigma) for 5 min. Antigen retrieval was then carried out by boiling in Tris/EDTA buffer (10 mM Tris, 1 mM EDTA, 0.05% Tween-20, pH 9.0) for 20 min. After cooling to room temperature, sections were washed in ddH_2_O then PBS, incubated in blocking solution (Cat # MB-0710100; Rockland) for 60 min, then incubated with primary antibodies (rabbit anti-Brd4; 1:200; Cat # ab128874; Abcam; goat abti-Iba1; 1:500, Cat # ab5076, Abcam) for 48 h at 4 °C. After three washes in PBS for 5 min each, sections were incubated with secondary antibodies (goat anti-rabbit IgG Alexa Fluor 488; donkey anti-goat IgG Alexa Fluor 555; 1:1000; Invitrogen) for 60 min at room temperature. Slides were washed with PBS, incubated with DAPI for 5 min at room temperature, then mounted for microscopy.

### Western blot

Zebrafish were dissected, then the head region lysed and extracted with RIPA buffer (150 mM NaCl, 10 mM Tris-HCl, 1 mM EDTA, 1% Triton X-100, 0.1% SDS, and 0.1% Sodium deoxycholate) or DIGE buffer (7 M Urea, 2 M Thiourea, 30 mM Tris-HCl, 4% CHAPS). For dephosphorylation assays in Fig. 3b, samples were extracted with high salt buffer and proteins precipitated with saturated ammonia sulfate. The pellet was resuspended in 50 mM Tris-HCl (pH8.0), 1 mM MgCl_2_ and incubated with calf intestinal alkaline phosphatase (CIP; cat # M0525; New England Biolabs) or λ-protein phosphatase (λPP; cat # P0753; New England Biolabs) at 37 °C for 60 min before electrophoresis.

60 μg crude protein extract from each experimental group was loaded in each well of a 12% SDS-PAGE gel. After electrophoretic separation, proteins were transferred to nitrocellulose membrane (LI-COR), which was blocked using Odyssey blocking buffer (cat # 927-40000; LI-COR) for 60 min at room temperature followed by incubation with primary antibody (see below) at 4 °C for 16 h. After 3 washes of 10 min each in PBST, blots were incubated with secondary antibodies IRDye 800CW goat anti-Mouse IgG (1:10000; cat # 926-32210, LI-COR) and IRDye 680RD goat anti-Rabbit IgG (1:10000; cat # 926-68071, LI-COR) in LI-COR blocking buffer for 60 min at room temperature in the dark. After 3 further washes in PBST, blots were scanned using a LI-COR Odyssey near infrared scanner and immunoreactive bands were quantified from the raw scan data using LI-COR Empiria software. To display qualitative features in the figures, raw scan data were reversed to yield images with dark bands on a light background and then brightness and contrast adjusted until the features of interest were visible (all adjustments were made equally across the entire image). Full-length blot images are shown in Source Data where applicable.

Primary antibodies and dilutions for western blots were as follows. Figure 2g: mouse anti-Tyrosine Hydroxylase (1:1000; cat # MAB318, Millipore), rabbit anti-GAD65/67 (1:500; cat # AB1511, Millipore), rabbit anti-Synaptophysin (1:500; cat # ab32594, Abcam), rabbit anti-PSD95 (1:500; cat # ab18258, Abcam). Figures 3a–c, 4a, b, d, e, g, and 8f, i: mouse anti-Tau[210-241] (Tau5; 1:1000; cat# AHB0042, Thermo Fisher), rabbit anti-human Tau[243-441] (1:5000; Cat# A0024, Dako), mouse anti-human phospho(S202/T205)-Tau (AT8; 1:500, Cat# MN1020, Thermo Fisher), mouse anti-human phospho(T231)-Tau (AT180; 1:500; Cat# MN1040, Thermo Fisher), mouse anti-human phospho(T181)-Tau (AT270; 1:2000; Cat# MN1050, Thermo Fisher), mouse anti-human phospho(T212/S214)-Tau (AT100; 1:250; Cat# MN1060, Thermo Fisher), mouse anti-human phospho(S396/S404)-Tau IgG1 (1:1000; a gift from Dr. Peter Davies, Albert Einstein College of Medicine), rabbit anti-human phospho(S422)-Tau (1:1000; Cat# 44-764G, Thermo Fisher). Figure 10b: rabbit anti-Brd4 antibody (1:500; a gift from Dr. Igor Dawid, NIH)^50^. Supplementary Fig. S7: rabbit anti-Caspase 3 (1:500; Cat# ab13847, Abcam). Rabbit anti-Actin (1:2000; cat # A2066, Sigma) was used to confirm equal protein loading on blots.

For the non-denaturing, non-reducing gel shown in Fig. 4g, zebrafish were mechanically dissociated in TBS buffer (50 mM Tris, pH7.4, 274 mM NaCl, 5 mM KCl, 1 mM PMSF, 1x protease inhibitor cocktail; Roche, cat # 11836153001). The supernatant containing TBS-soluble proteins was mixed with 4x non-reducing sample buffer (ThermoFisher; cat # 84788) and samples were loaded without boiling onto a 10% polyacrylamide gel. Following electrophoretic separation of the proteins, western blot was completed as above.

### Analysis of motor activity

Measurement of larval zebrafish motor activity (Figs. 5a–h, 7a, b, d, e, 8a, b, and 10c, d) was carried out using our open-source MATLAB applications *LSRtrack* and *LSRanalyze*^37^ exactly as described in our prior work^29,81^ and in our detailed published protocol^35^. Briefly, 5dpf larvae were transferred to 96 well plates with black well surrounds and optical glass bottoms (Corning 96-well Special Optics Microplate; cat # CLS3720; Sigma) using a large-bore Pasteur pipette with a flame-polished aperture, then acclimatized to the recording chamber for 30 min at 28.5˚C in white light (200 lx, color temperature 3500 K). Motor responses were recorded under continuous ambient illumination for 60 min, and then the visual motor response was elicited during 3 cycles each of (10 min dark + 10 min light). Recordings of trans-illuminated zebrafish were made through the glass bottom of the 96-well plate, with an infrared light source (#BL812-880, Spectrum Illumination, Montague, MI) positioned above and a USB 3.0 camera (#FL3-U3-13Y3M-C, Point Grey Research, Richmond, BC, Canada) with a 50 mm lens and IR-pass filter positioned 1.2 m below. Video recordings were analyzed offline.

### Analysis of ‘O’-bend kinematics

Analysis of truncal kinematics during swimming (Fig. 5i–m) was carried out using our open-source MATLAB application *HiSpeedTracking*, exactly as reported in our previous work^38^. Briefly, 5dpf zebrafish were transferred to 15 mm diameter wells cut into a 0.3% agarose-filled plate and allowed to acclimatize to the recording chamber at 28.5 °C for 30 min. ‘O’-bend responses were elicited over 40 ‘dark flash’ stimulus cycles of abrupt transition between bright white light ambient illumination (1100 lx, 4900 K) and dark (<1 lx). Video was captured from below the plate under infrared illumination as described above but using a high-speed camera (Integrated Design Tools, Pasadena, CA; model #NX8- S2) with a macro lens (Rokinon 100 mm f2.8; B&H, New York) and infrared-pass filter (R72, 720 nm; B&H). Video segments of 1 s duration at 1000 frames/s were captured at each light-dark transition, synchronized to the stimulus using a USB relay. Video recordings were analyzed offline.

### Analysis of optokinetic reflex responses

Analysis of optokinetic reflexes (OKR; Fig. 6) was carried out using our open-source MATLAB applications *OKRtrack* and *OKRanalyze*^40^, exactly as reported in our previous work^39^. Briefly, 5 dpf zebrafish larvae were immobilized in 3.5% methylcellulose on a transparent platform suspended within a cylindrical back-projection screen. Larvae were illuminated by an infrared light source from below (880 nm; #BL34-880, Spectrum, Montague, MI) and responses captured from above at 30 frames/s, using a zoom microscope (#S8 APO, Leica Microsystems, Wetzlar, Germany) with an IR-sensitive camera (#FL3-U3-13Y3M-C, Point Grey Research, Richmond, BC, Canada). A sinusoidal grating pattern (15°/cycle, 99% contrast, sinusoidally-transformed spatially to appear linearly spaced from within the cylinder) was projected onto the screen so that the pattern filled the left visual field of the zebrafish, and the stimulus was animated so it appeared to move at 15°/s (=1 cycle/s). Reflex gain was analyzed over 60 s of stimuli alternating every 10 s between nasotemporal (NT) and temporonasal (NT) directions. Optokinetic nystagmus was then provoked by uninterrupted NT or TN stimuli for 60 s in each direction to evaluate saccades and movement range. Video recordings were analyzed offline.

### Small molecule exposure and phenotypic screening

The epigenetics screening library (Cayman Chemical, Ann Arbor, MI; cat # 11076; batch # 0468238) used in Fig. 7 consisted of 147 small molecule modulators dissolved in DMSO at 10 mM. Zebrafish were exposed to compounds in 6-well plates, starting at 2 dpf, with water changes and fresh compound at 3dpf and 4dpf, and phenotypic readouts at 5dpf. The first stage of the screen involved testing each compound in WT zebrafish at 50 µM in E3 embryo buffer (yielding a maximum DMSO concentration of 0.5%, which is within the limit of tolerance for zebrafish larvae^87,88^). Any compound causing overt toxicity (death, morphological abnormalities by light microscopy, or obvious neurological problems) was tested at progressively lower concentrations until the maximum tolerated concentration (MTC) of each compound had been established. Tau zebrafish were then exposed to the MTC for each compound from 2 to 5 dpf as above, in groups of 12 zebrafish per well of a 6-well plate. At 5dpf, compounds were washed off and zebrafish transferred in fresh E3 buffer to 96-well plates for motor activity measurements as described above. Groups of 12 Tau and non-expressing sibling zebrafish, grown in parallel under identical conditions but without small molecule exposure, provided controls for the motor activity assays. All experiments were completed without reference to the identity of the compounds; the library key was only revealed once analysis of screening data was completed. Data analysis workflows were automated using custom MATLAB scripts to ensure assay QC criteria were met, and to quantify small molecule rescue of the Tau phenotype across the VMR, while averaging responses of individual zebrafish to multiple light cycles to minimize variability. (+)JQ1 and (−)JQ1 were repurchased from Sigma (cat #s SML1524 and SML1525) for the experiments shown in Figs. 8–10.

### Rat primary cortical cultures

Rat cortical cultures were prepared as reported previously^46^. Briefly, embryonic day 16 – 17 (E16 – 17) Sprague–Dawley rat cortices were dissociated and plated on poly-L-ornithine–coated glass coverslips (Carolina, Burlington, NC; #633029) in six-well plates (670,000 cells/well), and cultured in a v/v mixture of: 77.5% Dulbecco’s modified minimal essential medium (DMEM-Glutamax; Thermo Fisher, Waltham, MA; #10566016), 10% F-12 Ham nutrient mixture (Sigma; #N6658), 10% bovine calf serum (Cytivia; #SH30072), with 25 mM HEPES (Thermo Fisher; #15630080) and 0.24% penicillin/streptomycin (Sigma-Aldrich; P0781) at 37 °C in 5% CO_2_. Fifty percent media volume was replaced at DIV 3, 7, and 10 with growth medium as above, supplemented with 100 ng/mL mouse IL-34 (Biolegend, San Diego, CA; #577602), 2 ng/mL TGF-β (Thermo Fisher/Peprotech, #100-21), and 1.5 μg/mL ovine wool cholesterol (Avanti Polar Lipids, Alabaster, AL; #700000P) to support the survival, growth, and differentiation of microglia^47^. Plasmids encoding (i) Human 0N/4R-Tau with membrane-anchored GFP (mGFP), or (ii) mGFP alone, were prepared using an EndoFree Plasmid Maxi Kit (Qiagen; #12362) to eliminate bacterial endotoxin carryover that could itself activate microglia. At DIV14, the growth medium was changed to DMEM with 2% bovine calf serum and 25 mM HEPES and cultures were transfected in 24-well plates with 1.5 μg plasmid DNA, 4 μL Lipofectamine 2000 (Thermo Fisher; #11668030) and 100 μL Opti-MEM (Thermo Fisher; # 31985062). Cells were allowed to express 0N/4R-Tau for 5 days, then (+)JQ1 at a final concentration of 1 μM (or an equivalent amount of DMSO only) was added to the culture medium for 24 h before samples were fixed for immunohistology.

### Immunohistology of primary cultures

After fixing in 4% paraformaldehyde and washing with PBS, samples were incubated with blocking solution (Rockland, Limerick, PA; #22159) for 2 h at room temperature, then with primary antibodies to PSD95 (1:1000; Thermo Fisher; MA1-046), ionized calcium binding adaptor molecule 1 (Iba1; 1:1000; WAKO; #019-19741) and GFP (1:1000; Abcam; #ab13970) in PBS at 4 °C overnight. After 3 × 5 min washes in PBS, samples were incubated for 2 h at room temperature with goat anti-mouse Alexa Fluor 647 (1:1000; Invitrogen; #A-21235), goat anti-rabbit Alexa Fluor 555 (1:1000; Invitrogen; #A-21428), and goat anti-chicken Alexa Fluor 488 (1:1000; Invitrogen; #A-11039). After 3 further PBS washes, samples were incubated in DAPI (0.2 μg/mL in PBS; Roche; #10236276001) and mounted for microscopy. Confocal images of labeled cells were acquired using a Nikon Ti2E inverted microscope with a 40x long working distance water immersion objective (CFI Apo LWD Lambda S 40XC WI NA 1.15) and Nikon AXR resonance scanning confocal system (Nikon USA, Melville, NY). Images were analyzed using NIS-Elements software version 5.30.05. Custom algorithms were developed using the General Analysis 3 module to quantify microglial volume and synaptic puncta inside microglia, in each 3D image stack. To eliminate bias, all images for each experiment were acquired using identical settings and quantified using the same parameters and thresholds.

### Statistics and reproducibility

All experiments were repeated in a minimum of three independent biological replicates (i.e., different animals, cultures, or samples, in experimental replicates carried out on different days). Many assays were also repeated in technical replicate, as indicated in figure legends. For experiments using human PSP and control postmortem material, samples from individual patients were considered biological replicates. Sample sizes were determined by power analysis where anticipated effect size and variability were available. Otherwise, sample sizes were determined by experimental sample availability and practical considerations such as throughput and capacity to analyze samples in parallel. For zebrafish motor assays, data points were removed if there were >5% tracking errors in individual wells, but all data from viable larvae were otherwise included in analysis. For other analyses, experiments were excluded and repeated if they failed technically (controls did not yield interpretable results, or QC metrics not met). Individual samples were excluded if no interpretable data were collected, for example tissue damaged during histological preparation, but all data were otherwise included in analysis. Unbiased automated assays were employed where possible, otherwise observers were blinded to sample identity. The small molecule library was screened blinded to compound identity. 2-tailed or 2-sided statistical tests were used throughout. Normally distributed data were analyzed using parametric statistical tests. *t*-test with Welch’s correction for unequal variance was used to compare 2 experimental groups. To compare 3 or more groups, 1- or 2-way ANOVA was used, depending on the experimental design and number of variables being evaluated, as detailed in each figure legend. Pairwise comparisons were carried out using Dunnett test to compare each group to a single control value, Tukey test to compare all groups with each other, or Šidák test where an experiment was designed specifically to compare particular pairs of groups from the set of possible permutations, or for repeated comparison of the same groups over time. One-sample *t*-test was used to compare an experimental group to a normalized (e.g. rescue = 0, or expression = 1) control. Survival data were analyzed by Mantel–Cox test. Categorical data were analyzed using Fisher’s exact test. All analyses were completed using Prism 10.2.2 (GraphPad).

### Reporting summary

Further information on research design is available in the Nature Portfolio Reporting Summary linked to this article.

## Supplementary information

Supplementary Information

Peer Review File

Description of Additional Supplementary Files

Supplementary Movie 1

Reporting Summary

## Source data

Source Data

## Data Availability

All data supporting the findings of this study are shown within the paper and Supplementary Figs. Reasonable requests for access to additional resources such as raw data should be addressed to the corresponding author. Source data are provided with this paper.

## Code availability

The MATLAB applications we developed for measuring zebrafish motor activity in 96-well plates^35,37^, zebrafish swimming kinematics^38^, and zebrafish optokinetic reflexes^40^ were published previously. Source code is provided in the supplementary data for each of these publications.
