# Supplementary material for: A human Tau expressing zebrafish model of progressive supranuclear palsy identifies Brd4 as a regulator of microglial synaptic elimination
Source: Nat Commun. 2024 Sep 18;15:8195. doi: 10.1038/s41467-024-52173-0 (PMC11410960; doi:10.1038/s41467-024-52173-0)
Supplement: Supplementary file 1 — Supplementary Information [file 41467_2024_52173_MOESM1_ESM.pdf]

***Supplementary figures and tables to accompany:***

# A human Tau expressing zebrafish model of progressive supranuclear palsy identifies Brd4 as a regulator of microglial synaptic elimination

Qing Bai, Enhua Shao, Denglei Ma, Binxuan Jiao, Seth D. Scheetz, Karen A. Hartnett-Scott, Vladimir A. Ilin, Elias Aizenman, Julia Kofler, Edward A. Burton

Nature Communications (2024)

**Contents**

Supplementary Figure S1 – Transgene constructs and expression .......... 5

Supplementary Table 1 – Primer sequences for quantitative real-time PCR analysis of gene expression .......... 6

Supplementary Figure S2 – Verification of gene primers for reverse transcription PCR .......... 6

Supplementary Figure S3 – Verification of reference gene primers for real-time quantitative RT-PCR .......... 7

Supplementary Figure S4 – Verification of human and zebrafish Tau primers for real-time quantitative RT-PCR .......... 8

Supplementary Table 2 – Amplification efficiency of each primer pair .......... 9

Supplementary Figure S5 – Expression of human Tau transgene relative to the endogenous zebrafish Tau paralogues *mapta* and *maptb* .......... 10

Supplementary Table 3 – Abbreviations and genotypes of zebrafish lines used in this study .......... 11

Supplementary Table 4 – Statistical analysis of survival in Tau zebrafish .......... 12

Supplementary Figure S6 - Survival of [GFP + mCherry] and [α-Synuclein + mCherry] zebrafish .......... 13

Supplementary Table 5 – Statistical analysis of survival in [GFP + mCherry] and [α-Synuclein + mCherry] zebrafish .......... 14

Supplementary Table 6 – ANOVA summary for Figure 2d (Acridine Orange labeling) .......... 15

Supplementary Table 7 – ANOVA summary for Figure 2e (TUNEL labeling) .......... 16

Supplementary Table 8 – ANOVA summary for Figure 2f (activated Caspase-3 labeling) .......... 17

Supplementary Figure S7 – Caspase 3 cleavage by Western blot in Tau zebrafish at 3 – 5 dpf .......... 18

Supplementary Figure S8 – Quantification of labeled CNS cells in zebrafish larval sections .......... 19

Supplementary Table 9 – Exact *p*-values for Figure 2g .......... 20

Supplementary Table 10 – ANOVA summary for Figure 2i (7.4.C4$^{+}$ microglia) .......... 21

Supplementary Figure S9 – No evidence that human Tau is transferred to cells not expressing the transgene .......... 22

Supplementary Figure S10 – Serial extraction of Tau zebrafish in RIPA then DIGE .......... 23

Supplementary Figure S11 – Relative expression of human MAPT mRNA during development in transgenic Tau zebrafish .......... 24

Supplementary Table 11 – Šidák multiple comparisons test for Figure 5b .......... 25

Supplementary Figure S12 – Biological replicate motor activity assays comparing Tau and Ctrl zebrafish .......... 26

Supplementary Table 12 – ANOVA summary for Supplementary Figure S12 .......... 27

Supplementary Figure S13 – Three different control groups behave similarly in motor activity assays .......... 28

Supplementary Figure S14 – Heterologous protein over-expression does not alone cause motor phenotypes .......... 29

Supplementary Figure S15 – Methodology and abnormalities of ocular movement in Tau zebrafish .......... 30

Supplementary Figure S16 – Abnormalities of the visual motor response in Tau zebrafish .......... 31

Supplementary Table 13 – Šidák multiple comparisons table for Supplementary Figure S16b .......... 32

Supplementary Table 14 – Small molecule screening data .......... 33

Supplementary Figure S17 – Small molecule screen part 1: Determination of maximum tolerated concentration .......... 34

Supplementary Figure S18 – Small molecule screen part 2: Rescue of motor phenotype in Tau zebrafish ........................ 35
Supplementary Figure S19 – Algorithm logic for analysis of video tracking data from rescue screening assays ................ 36
Supplementary Figure S20 – Example outputs from screening assay ........................................................................ 37
Supplementary Table 15 – Summary of data from the small molecule screen in Figure 7d ........................................ 38
Supplementary Figure S21 – Hits recovered from epigenetic library screen ............................................................ 42
Supplementary Table 16 – Data summary by target ..................................................................................................... 43
Supplementary Figure S22 – Ranked phenotypic rescue activity by target ............................................................... 44
Supplementary Table 17 – Properties of compounds categorized as 'other' in Figure 7e ........................................... 45
Supplementary Figure S23 – Verification of hits by analysis of repurchased compound ............................................ 46
Supplementary Figure S24 – Concentration-dependent rescue of motor function in Tau zebrafish by (+)JQ1 ................. 47
Supplementary Table 18 - Exact *p*-values for Figure 8b, 8d – 8i ................................................................................. 48
Supplementary Figure S25 – Specificity controls for (+)JQ1 rescue of motor function in Tau zebrafish ........................ 49
Supplementary Figure S26 – (+)JQ1 does not silence transgene expression in Tau zebrafish ...................................... 50
Supplementary Table 19 – Statistical analysis of Tau zebrafish survival in presence or absence of (+)JQ1 ..................... 51
Supplementary Table 20 – Statistical analysis of Ctrl zebrafish survival in presence or absence of (+)JQ1 ...................... 51
Supplementary Figure S27 – (+)JQ1 does not rescue OKR deficits in Tau zebrafish ....................................................... 52
Supplementary Figure S28 – Volumetric imaging of synapse abundance in zebrafish larval brain .................................. 53
Supplementary Table 21 – Exact *p*-values for Figure 9b, 9c, 9e, 9g ............................................................................... 54
Supplementary Figure S29 – Quantification of PSD95-immunoreactive synaptic material inside zebrafish microglia ....... 55
Supplementary Figure S30 – (+)JQ1 mitigates microglial accumulation of PSD95-immunoreactive synaptic material in Tau zebrafish ............................................................................................................................................................ 56
Supplementary Figure S31 – Rat primary co-culture system to test the activity of (+)JQ1 in mammalian microglia .......... 57
Supplementary Figure S32 – Quantification of PSD95-immunoreactive puncta inside rat microglia ............................... 58
Supplementary Figure S33 – (+)JQ1 mitigates accumulation of PSD95-immunoreactive puncta within rat microglia co-cultured with neurons transfected to express human 0N/4R-Tau ........................................................................ 59
Supplementary Figure S34 – Transient knockdown of Brd4 in Tau zebrafish mitigates microgliosis at 4dpf ..................... 60
Supplementary Figure S35 – Generation of a stable *brd4* null allele using Cas9/CRISPR .................................................... 61
Supplementary Figure S36 – Developmental phenotypes in *brd4*$^{-/-}$ zebrafish ................................................................ 62
Supplementary Figure S37 – Motor activity abnormalities in *brd4*$^{-/-}$ zebrafish ............................................................... 63
Supplementary Figure S38 – Heterozygous *brd4*$^{+/-}$ mutations partially rescue motor phenotypes in Tau zebrafish ......... 64
Supplementary Table 22 – Exact *p*-values for Figure 10d – 10i ........................................................................................ 65
Supplementary Figure S39 – Heterozygous *brd4*$^{+/-}$ mutation does not rescue OKR deficits in Tau zebrafish ..................... 66
Supplementary Figure S40 – Rescue of synaptic puncta in Tau zebrafish with heterozygous *brd4*$^{+/-}$ mutations ................ 67
Supplementary Figure S41 – Brd4 expression in the nuclei of human substantia nigra microglia ..................................... 68

Supplementary Figure S42 – Brd4 expression in microglia from multiple control and PSP cases .......................................69
References ........................................................................................................................................................ 70

**Supplementary Figure S1 – Transgene constructs and expression**

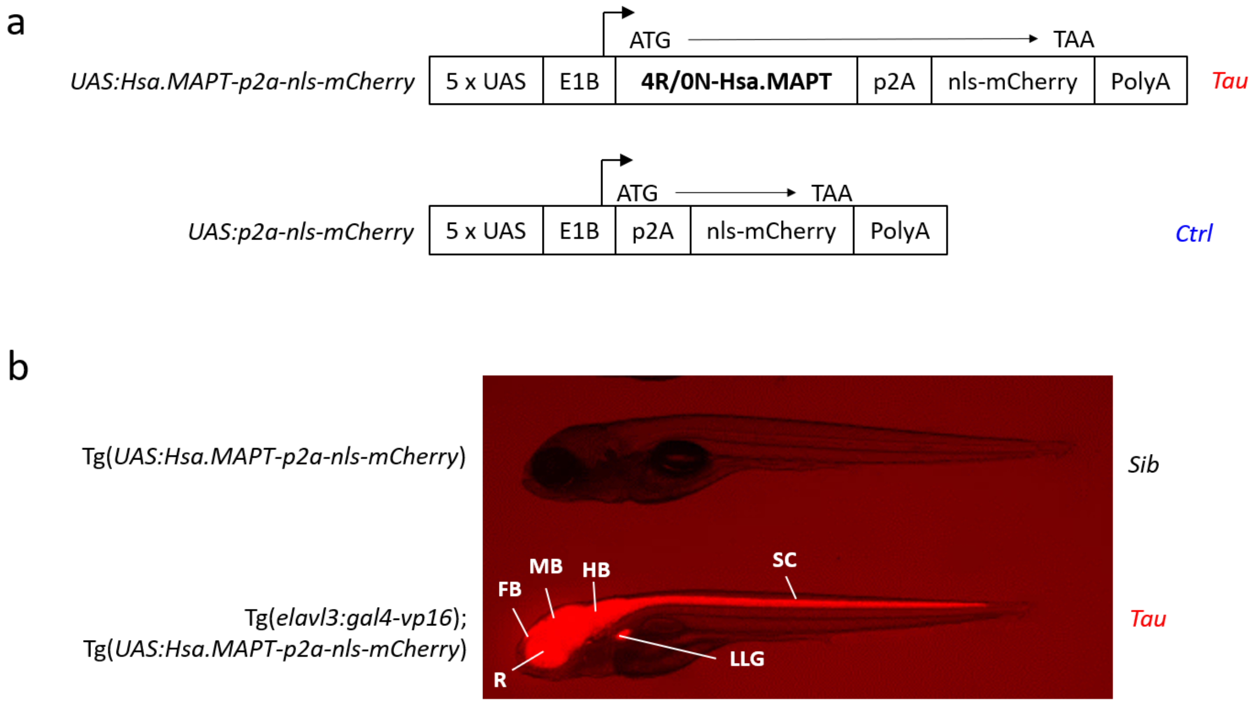


a: Schematic maps (not to scale) of the transgene constructs used to make Tau and Ctrl zebrafish. Key: UAS, upstream activating sequence; E1B, minimal promoter; p2A, self-cleaving viral 2A peptide; 0N/4R-Hsa.MAPT, human Tau cDNA lacking exons 2 and 3 but including exon 10, encoding human 0N/4R-Tau; nls-mCherry, mCherry fluorescent protein fused to a nuclear localization signal; PolyA, polyadenylation signal. Positions of the open reading frames are indicated, illustrating the bicistronic cassette in the Tau-2A-nls-mCherry construct.

b: Widefield epifluorescence image of a Tg(*elavl3:gal4-vp16*); Tg(*UAS:Hsa.MAPT-2a-nls-mCherry*) 'Tau' zebrafish and its Tg(*UAS:hsa.MAPT-2a-nls-mCherry*) sibling at 5 dpf, illustrating robust transactivation of the transgene in the forebrain (FB), midbrain (MB), hindbrain (HB), retina (R), spinal cord (SC) and lateral line ganglion (LLG) by the pan-neuronal Tg(*elavl3:gal4-vp16*) driver.

**Supplementary Table 1 – Primer sequences for quantitative real-time PCR analysis of gene expression**

| Species* | Gene | 5' Primer sequence | 3' Primer sequence | Product | Type§ |
|---|---|---|---|---|---|
| Dre | *bact1* | ACCGAGCGTGGCTACAGCTT | TCCCATCTCCTGCTCGAAGTC | 105bp | R |
| Dre | *gapdh* | AGATCGTGGCCATCAATGACC | CGCCTTCTGCCTTAACCTCAC | 108bp | R |
| Dre | *mapta* | AGCAGCCCTGGCACTCCTAA | AGGGGTCGAGCGAATCACTG | 105bp | E |
| Dre | *maptb* | CTAAAACCCCAGACCGCAGTG | TGATTTGGGTGGGGTGCGTA | 104bp | E |
| Hsa | MAPT | AAAACGAAGATCGCCACACCG | TGGGTGGTGTCTTTGGAGCG | 109bp | T |
| Dre | 18S rRNA | TGTTCAAAGCAGGCCGCCCA | AATCATGGCCCCGGGTTCCA | 104bp | R |

**Species: Dre = Danio rerio (zebrafish); Hsa = Homo sapiens (human)*

*§Type: R = reference gene; E = endogenous Tau paralogue; T = human Tau transgene*

The table shows the primer sequences used for real-time quantitative PCR analysis of gene expression in Supplementary Figures S2 – S5 and S11. Primers were designed to (i) avoid alternatively spliced regions of transcripts; (ii) distinguish between closely related genes; (iii) yield amplicons of similar size; (iv) avoid duplex formation and self-annealing.

**Supplementary Figure S2 – Verification of gene primers for reverse transcription PCR**

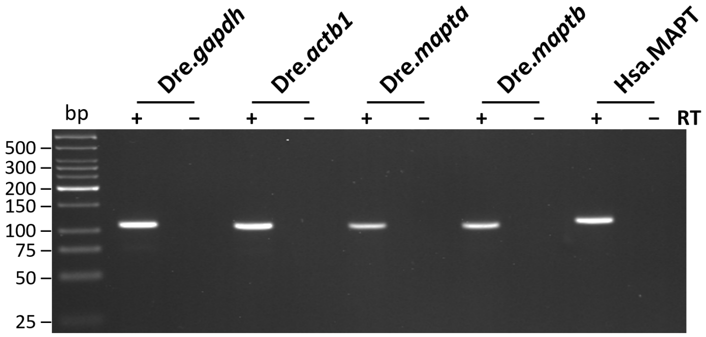


Prior to validating primer pairs for quantitative real-time PCR analysis, we completed 35-cycle PCR amplification of first strand cDNA from 5dpf Tau zebrafish. PCR products were separated by electrophoresis on a 4% LMW agarose gel. The picture shows the ethidium-stained gel photographed under UV illumination. Each primer pair generated a product of the expected size. Amplification was specific to RNA samples that had undergone reverse transcription (RT+). These data validate use of each primer pair to detect the intended target transcript in first-strand cDNA from Tau zebrafish.

**Supplementary Figure S3 – Verification of reference gene primers for real-time quantitative RT-PCR**

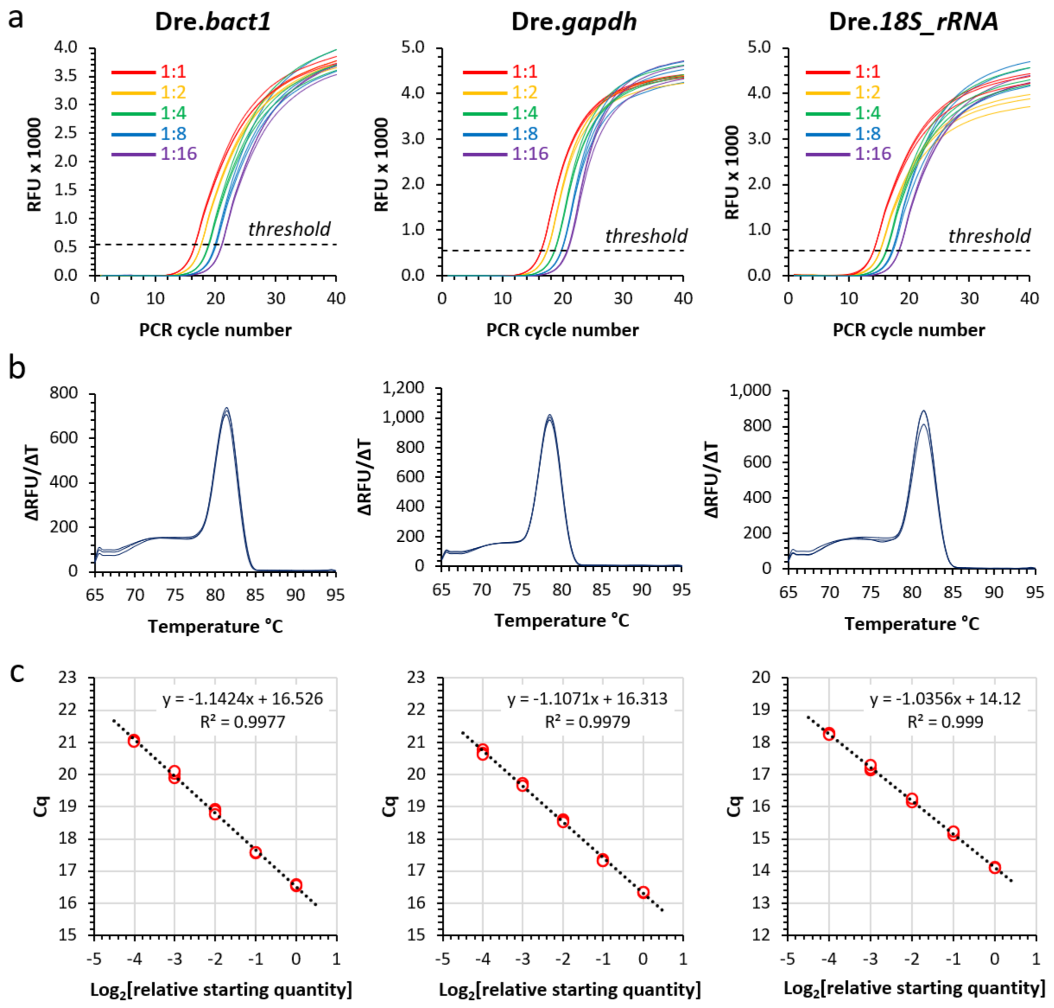


First-strand cDNA from 5dpf Tau zebrafish was subjected to real-time qPCR analysis, using a BioRad CFX Maestro system and software to quantify SYBR-Green fluorescence during amplification with primers for the reference genes Dre.*bact1* (left), Dre.*gapdh* (center), and Dre.*18S_rRNA* (right).

a: Amplification curves showing relative fluorescence units (proportional to product abundance; *y*-axis) as a function of PCR cycle (*x*-axis) for technical triplicates of each serial 2-fold dilution of the starting cDNA (color-coded according to the legend). Background subtraction and curve fitting were completed by the CFX-Maestro software. Threshold fluorescence for quantification was determined by the software algorithm to ensure each curve crossed the threshold during the exponential phase of amplification.

b: Melt curve analysis for each amplicon showing change in fluorescence signal with temperature (*y*-axis) as a function of temperature (*x*-axis). A single sharp peak, indicating a single amplification product, is seen for each primer pair. For clarity three technical replicates are shown of the amplicon from undiluted cDNA.

c: Graphs showing cycle of quantification (Cq; cycle at which amplification curve crosses threshold fluorescence in panel A) as a function of $\log_2$[relative cDNA starting quantity ($\propto$ 1/dilution factor)]. Three technical replicates are shown at each dilution. The linear regression line and equation are shown for each primer pair. $R^2$ values were > 0.99 in all cases.

These data validate use of these reference gene primer pairs for real-time qPCR analysis of Tau zebrafish.

**Supplementary Figure S4 – Verification of human and zebrafish Tau primers for real-time quantitative RT-PCR**

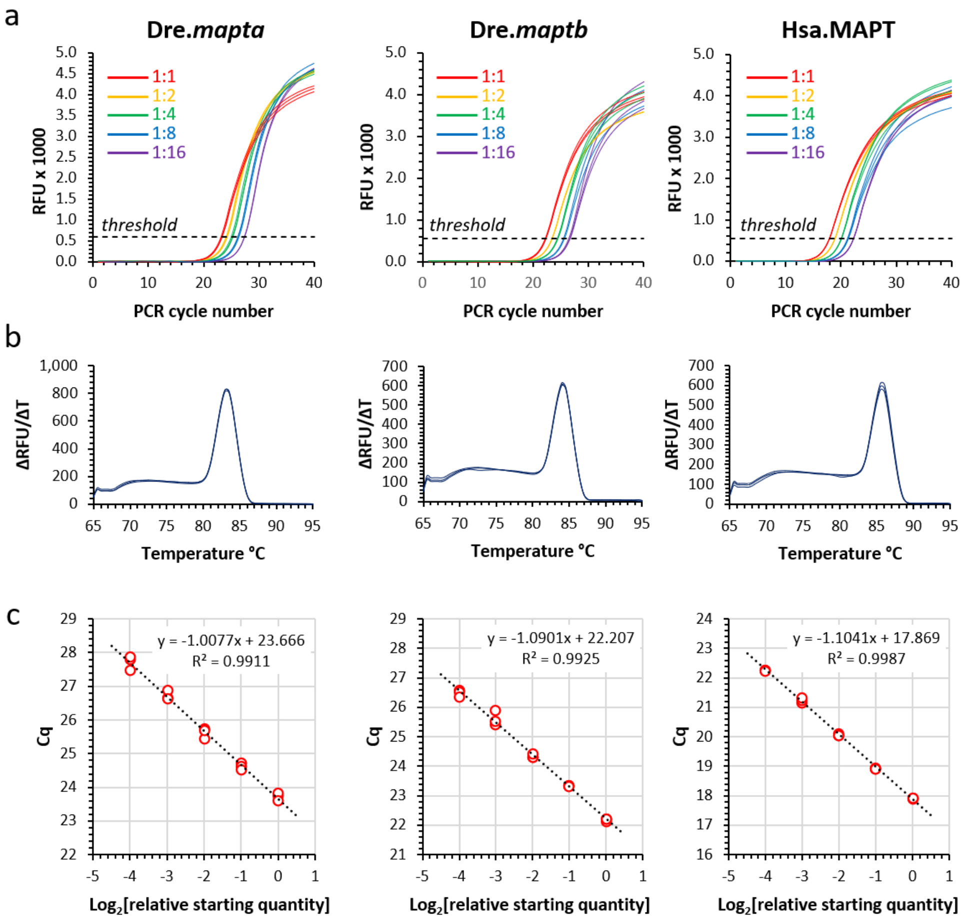


d

$$E_{gene} = 2^{\left(\frac{-1}{slope}\right)}$$

e

| Gene | E |
|---|---|
| Dre.*mapta* | 1.989471268 |
| Dre.*maptb* | 1.888648986 |
| Hsa.*MAPT* | 1.873494377 |

First-strand cDNA from 5dpf Tau zebrafish was subjected to real-time PCR analysis, similar to Supplementary Figure S3, but using primers for the dual zebrafish Tau paralogues, Dre.*mapta* (left) and Dre.*maptb* (center), and the human Tau transgene, Hsa.MAPT (right).

a: Amplification curves similar to Supplementary Figure S3a.

b: Melt curve analysis similar to Supplementary Figure S3b. A single sharp peak, indicating a single amplification product, is seen for each primer pair.

c: Graphs showing cycle of quantification as a function of $log_2$[relative cDNA starting quantity], similar to Supplementary Figure S3c. The linear regression line and equation are shown for each primer pair. $R^2$ values were > 0.99 in all cases.

These data validate the use of these primer pairs for real-time qPCR analysis of Tau zebrafish.

**Supplementary Table 2 – Amplification efficiency of each primer pair**

| Gene | E |
|---|---|
| Dre.*mapta* | 1.989471268 |
| Dre.*maptb* | 1.888648986 |
| Hsa.*MAPT* | 1.873494377 |

The amplification efficiency, $E$, for each Tau primer pair was calculated using the equation:

$$E_{gene} = 2^{\left(\frac{-1}{slope}\right)}$$

Where $slope$ = the gradient of the linear regression line for each gene in Supplementary Figure S4C.

The amplification efficiency relates the starting abundance, $A$, of the target cDNA in the sample to the abundance of the amplicon, $P$, after $n$ cycles of PCR:

$$P = A \times E^n$$

Correction for the differing amplification efficiency of each primer pair is essential for meaningful comparison of the abundance of different target mRNA species using this method.

**Supplementary Figure S5 – Expression of human Tau transgene relative to the endogenous zebrafish Tau paralogues *mapta* and *maptb***

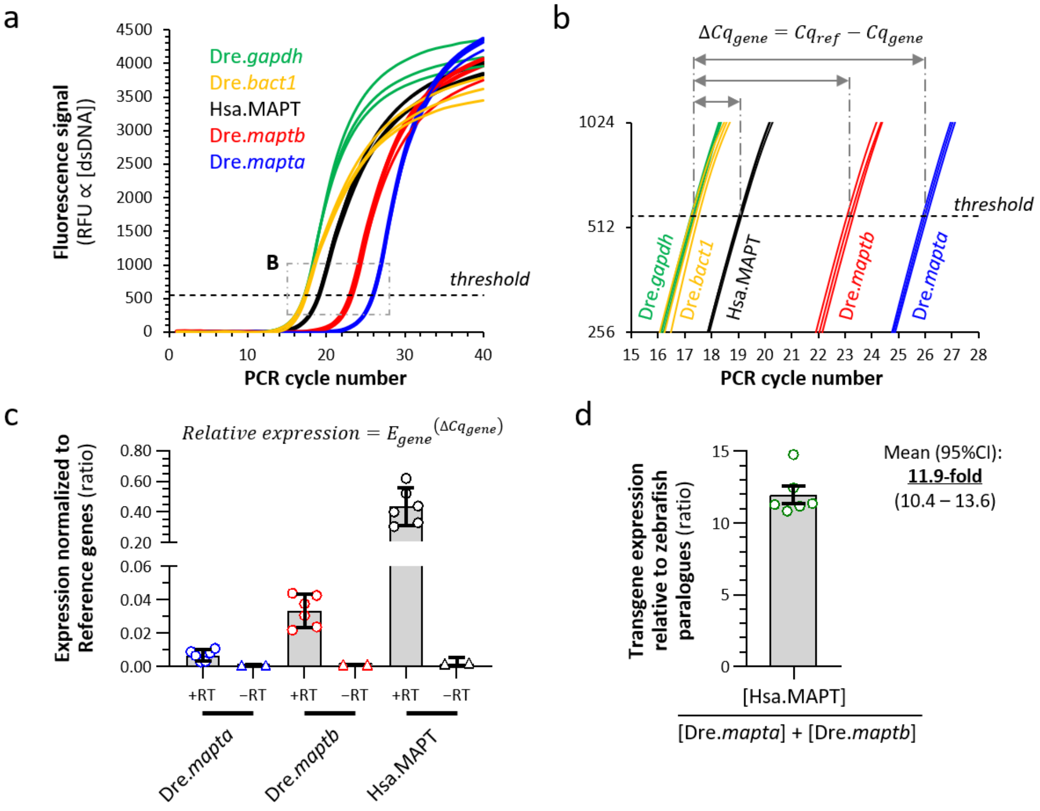


First-strand cDNA from Tau zebrafish at 5dpf was amplified by real-time quantitative PCR using primers for two zebrafish reference genes (Dre.*bact1*, Dre.*gapdh*), the dual zebrafish Tau paralogues Dre.*mapta* and Dre.*maptb*, and the Human Tau transgene, Hsa.MAPT. Three technical replicates were completed for each of six biological replicates (i.e. six different first-strand cDNA libraries derived from different Tau zebrafish at 5dpf), run across two replicate experiments.

a: Amplification curves are shown for one biological replicate. The curves for all 3 technical replicates of all 5 primer pairs are superimposed (genes are color coded according to the legend). The boxed area is shown in panel (b). The quantification threshold was determined by the software algorithm to ensure amplification of each target transcript was in its exponential phase at threshold.

b: The boxed area from panel (a) is expanded to illustrate the linear relationship between $\log_2$[fluorescence signal (∝ product abundance)] and PCR cycle around the quantification threshold. The difference between Cq for each gene of interest and mean Cq for the reference genes was calculated in each biological replicate, allowing comparison of the abundance of *mapta*, *maptb* and MAPT relative to the reference genes in each sample.

c: Normalized expression of each gene relative to the reference genes was calculated using the formula $E_{gene}^{(\Delta Cq_{gene})}$, as shown. Each point shows the mean value of three technical replicates for each different biological replicate, bars show group mean ± SE. Note the discontinuous y-axis to accommodate widely differing relative expression levels. RNA samples that were not reverse transcribed were also amplified in each experimental replicate to reveal any contribution to the measured signal from genomic DNA carry over. Whole-larval human Tau transgene mRNA was expressed at approximately 40% abundance of the reference genes, whereas the endogenous zebrafish Tau paralogues were expressed at much lower levels.

d: For each biological replicate, the ratio of transgene Tau (in reference gene units) to the sum of the two endogenous paralogues (also in reference gene units) was calculated. Data points show each biological replicate, bars show mean ± 95% CI. Overall, transgenic human MAPT mRNA was ≈12-fold more abundant than the two endogenous paralogues, in whole zebrafish at 5dpf.

Panels c and d are identical to Figure 1c and 1c’ of the manuscript and are reproduced here for clarity.

**Supplementary Table 3 – Abbreviations and genotypes of zebrafish lines used in this study**

| Abbreviation | Genotype |
|---|---|
| Tau | Tg(*elavl3:gal4-vp16*); Tg(*UAS:Hsa.MAPT-p2a-nls-mCherry*) |
| Ctrl | Tg(*elavl3:gal4-vp16*); Tg(*UAS:p2a-nls-mCherry*) |
| Sib | Siblings of Tau zebrafish without mCherry expression, includes a 1:1:1 ratio of:<br>(i) WT<br>(ii) Tg(*elavl3:gal4-vp16*)<br>(iii) Tg(*UAS:Hsa.MAPT-p2a-nls-mCherry*) |
| GFP | Tg(*elavl3:gal4-vp16*); Tg(*UAS:egfp*) |
| mCherry + GFP | Tg(*elavl3:gal4-vp16*); Tg(*UAS:p2a-nls-mCherry*); Tg(*UAS:egfp*) |
| α-Syn | Tg(*elavl3:gal4-vp16*); Tg(*UAS:Hsa.SNCA-p2a-nls-mCherry*) |

**Supplementary Table 4 – Statistical analysis of survival in Tau zebrafish**

| Group | Parameter | Replicate #1 | Replicate #2 | Replicate #3 |
|---|---|---|---|---|
| Ctrl vs. Tau | Mantel-Cox $\chi^2$ (df) | 50.44 (1) | 40.15 (1) | 77.02 (1) |
| | Mantel-Cox *p* | 0.000000000001230 | 0.000000000234980 | $< 10^{-15}$ |
| | Mantel-Haenszel hazard ratio (95% CI) | 13.51<br>(6.6 – 27.7) | 11.61<br>(5.4 – 24.8) | 31.00<br>(14.4 – 66.8) |
| Sib | Median survival | *undefined | *undefined | *undefined |
| Ctrl | | *undefined | *undefined | *undefined |
| Tau | | 8 days | 9 days | 8 days |

The table shows Mantel-Cox $\chi^2$ and *p*, and Mantel-Haenszel hazard ratio ± 95% confidence intervals for survival of Tau zebrafish in comparison with Ctrl zebrafish in each of the three replicate cohorts shown in Figure 1D. Similar results were found when comparing Tau and Sib zebrafish, and the statistical significance for both comparisons persisted after correction for multiple comparisons. In the lower part of the table, the calculated median survival for Tau zebrafish in each cohort is shown. The median survival for Ctrl and non-expressing sibling zebrafish in our zebrafish system is > 2 years and so was not reached (*undefined) in these experiments, which were deemed completed when no Tau zebrafish remained.

**Supplementary Figure S6 - Survival of [GFP + mCherry] and [α-Synuclein + mCherry] zebrafish**

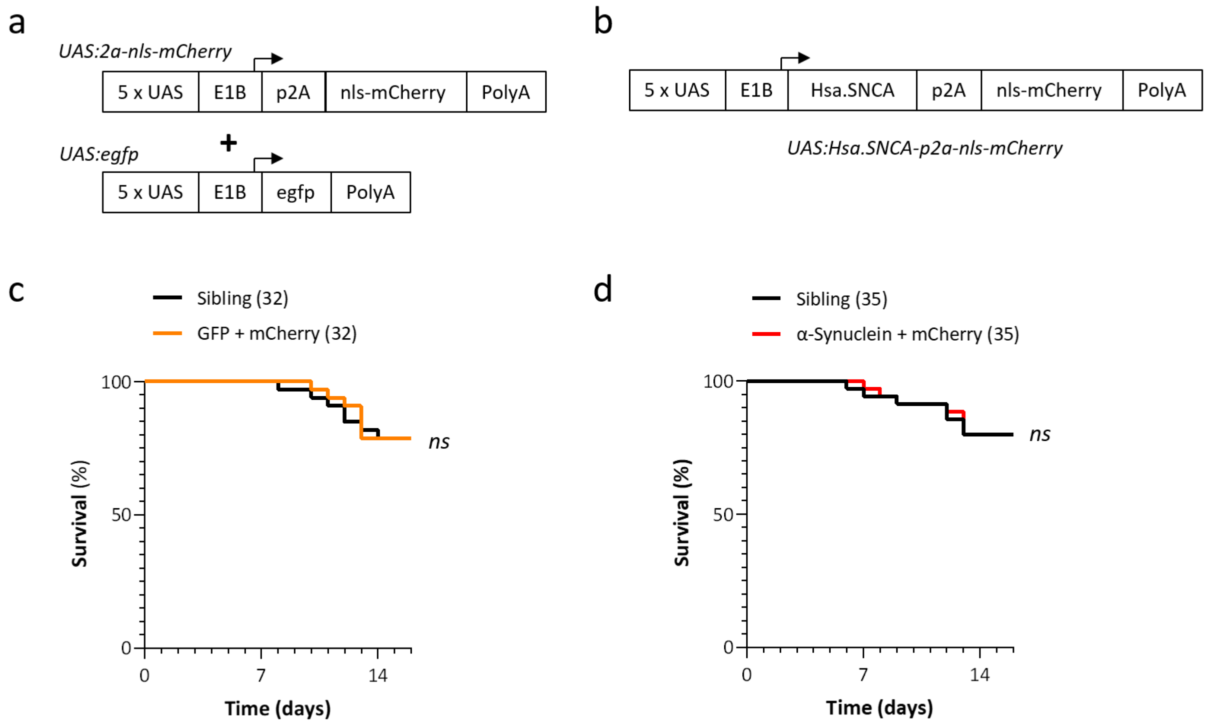


To test whether the compromised viability of Tau zebrafish (Figure 1d, e) is a non-specific consequence of simultaneously over-expressing two heterologous proteins (human WT 4R/0N-Tau and mCherry), we analyzed survival in two additional models that each express two transgenes under the same Tg(*elavl3:gal4-vp16*) driver used in the Tau model:

a: Tg(*elavl3:gal4-vp16*); Tg(*UAS:p2a-nls-mCherry*); Tg(*UAS:egfp*) zebrafish express both GFP and mCherry

b: Tg(*elavl3:gal4-vp16*); Tg(*UAS:Hsa.SNCA-p2a-nls-mCherry*)$^{pt423}$ zebrafish express both human α-Synuclein and mCherry. The UAS responder construct in this line is identical to Tau zebrafish, except that the human 4R/0N-Tau sequence is replaced by a cDNA encoding WT human α-Synuclein. We reported construction and validation of the pt423 transgenic line elsewhere[1].

c: Survival of Tg(*elavl3:gal4-vp16*); Tg(*UAS:p2a-nls-mCherry*); Tg(*UAS:egfp*) zebrafish (orange) compared with non-expressing siblings (black).

d: Survival of Tg(*elavl3:gal4-vp16*); Tg(*UAS:Hsa.SNCA-p2a-nls-mCherry*) zebrafish (red) compared with non-expressing siblings (black). This panel is identical to Figure 1f and is reproduced here for clarity and comparison.

For c and d, the starting numbers of zebrafish in each cohort are indicated in each graph legend.

Together with the statistical analysis shown in supplementary table 5, these data do not support the interpretation that the impaired survival of Tau zebrafish is a non-specific artifact of heterologous protein over-expression. Instead, this phenotype likely reflects a pathogenic property of human 0N/4R-Tau in this model.

**Supplementary Table 5 – Statistical analysis of survival in [GFP + mCherry] and [α-Synuclein + mCherry] zebrafish**

| **Comparison** | **Parameter** | | |
|---|---|---|---|
| | Mantel-Cox $\chi^2$ (df) | Mantel-Cox *p* | Mantel-Haenszel hazard ratio (95% CI) |
| Sib vs. mCherry + GFP | 0.0052 (1) | 0.94 | 0.96 (0.33 – 2.79) |
| Sib vs. Human α-Synuclein + mCherry | 0.0012 (1) | 0.97 | 0.98 (0.34 – 2.80) |
| *Sib vs. Human 4R/0N-Tau + mCherry** | *71.68 (1)* | *0.000000000003842* | *24.71 (11.76 – 51.91)* |
| ** Replicate #3 from Figure 1D, reanalyzed to compare Sib and Tau, similar to the other comparisons in this table* | | | |

The table shows Mantel-Cox $\chi^2$ and *p*, and Mantel-Haenszel hazard ratio ± 95% confidence intervals for survival of GFP/mCherry (Supplementary Figure S6C) or α-Synuclein/mCherry (Figure 1f, Supplementary Figure S6d) zebrafish in comparison with non-expressing Sibling zebrafish. The last row (in gray font) shows replicate #3 of Tau zebrafish (Figure 1d) reanalyzed to compare with non-expressing siblings, to provide a representative comparison with the additional controls.

**Supplementary Table 6 – ANOVA summary for Figure 2d (Acridine Orange labeling)**

2-way ANOVA (genotype, age) table:

| Source of Variation | % of total variation | *p* |
|---|---|---|
| *Interaction* | 10.76 | 0.000000000000239 |
| *Age* | 10.59 | 0.000000000000367 |
| *Genotype (Ctrl versus Tau)* | 48.13 | <0.000000000000001 |

| | SS (Type III) | DF | MS | F (DFn, DFd) | *p* |
|---|---|---|---|---|---|
| *Interaction* | 24385.55 | 5 | 4877.11 | F (5, 194) = 16.21 | P=0.000000000000239 |
| *Age* | 24005.98 | 5 | 4801.20 | F (5, 194) = 15.96 | P=0.000000000000367 |
| *Genotype* | 109092.54 | 1 | 109092.54 | F (1, 194) = 362.60 | P<0.000000000000001 |
| *Residual* | 58366.43 | 194 | 300.86 | | |

Šidák multiple comparisons test

| Comparison group 1 | Comparison group 2 | Predicted (LS) mean diff. | 95.00% CI of diff. | Adjusted *p* Value |
|---|---|---|---|---|
| Ctrl, 2 dpf | Tau, 2 dpf | -12.25 | -29.09 to 4.59 | 0.285789213561081 |
| Ctrl, 3 dpf | Tau, 3 dpf | -46.81 | -62.72 to -30.90 | 0.000000000001960 |
| Ctrl, 4 dpf | Tau, 4 dpf | -79.61 | -95.25 to -63.96 | <0.000000000000001 |
| Ctrl, 5 dpf | Tau, 5 dpf | -65.07 | -81.50 to -48.64 | <0.000000000000001 |
| Ctrl, 6 dpf | Tau, 6 dpf | -51.60 | -66.04 to -37.16 | <0.000000000000001 |
| Ctrl, 7 dpf | Tau, 7 dpf | -25.91 | -42.69 to -9.12 | 0.000359347541892 |

**Supplementary Table 7 – ANOVA summary for Figure 2e (TUNEL labeling)**

2-way ANOVA (genotype, age) table:

| **Source of Variation** | **% of total variation** | ***p*** |
|---|---|---|
| *Interaction* | 22.63 | <0.000000000000001 |
| *Age* | 39.03 | <0.000000000000001 |
| *Genotype (Ctrl versus Tau)* | 25.11 | <0.000000000000001 |

| | **SS (Type III)** | **DF** | **MS** | **F (DFn, DFd)** | ***p*** |
|---|---|---|---|---|---|
| *Interaction* | 211377.76 | 5 | 42275.55 | F (5, 91) = 28.63 | P<0.000000000000001 |
| *Age* | 364563.85 | 5 | 72912.77 | F (5, 91) = 49.37 | P<0.000000000000001 |
| *Genotype* | 234577.60 | 1 | 234577.60 | F (1, 91) = 158.84 | P<0.000000000000001 |
| *Residual* | 134387.46 | 91 | 1476.79 | | |

Šidák multiple comparisons test

| **Comparison group 1** | **Comparison group 2** | **Predicted (LS) mean diff.** | **95.00% CI of diff.** | **Adjusted *p* Value** |
|---|---|---|---|---|
| Ctrl, 2 dpf | Tau, 2 dpf | -12.25 | -29.09 to 4.59 | 0.001395687044297 |
| Ctrl, 3 dpf | Tau, 3 dpf | -46.81 | -62.72 to -30.90 | <0.000000000000001 |
| Ctrl, 4 dpf | Tau, 4 dpf | -79.61 | -95.25 to -63.96 | 0.000000000000055 |
| Ctrl, 5 dpf | Tau, 5 dpf | -65.07 | -81.50 to -48.64 | 0.012198377958896 |
| Ctrl, 6 dpf | Tau, 6 dpf | -51.60 | -66.04 to -37.16 | 0.999939505322290 |
| Ctrl, 7 dpf | Tau, 7 dpf | -25.91 | -42.69 to -9.12 | 0.729813635807312 |

**Supplementary Table 8 – ANOVA summary for Figure 2f (activated Caspase-3 labeling)**

2-way ANOVA (genotype, age) table:

| Source of Variation | % of total variation | *p* |
|---|---|---|
| *Interaction* | 15.14 | 0.000000060713328 |
| *Age* | 25.04 | 0.000000000008366 |
| *Genotype (Ctrl versus Tau)* | 34.76 | <0.000000000000001 |

| | SS (Type III) | DF | MS | F (DFn, DFd) | *p* |
|---|---|---|---|---|---|
| *Interaction* | 28418.35 | 5 | 5683.67 | F (5, 78) = 10.85 | P=0.000000060713328 |
| *Age* | 47000.02 | 5 | 9400.00 | F (5, 78) = 17.94 | P=0.000000000008366 |
| *Genotype* | 65239.59 | 1 | 65239.59 | F (1, 78) = 124.54 | P<0.000000000000001 |
| *Residual* | 40858.57 | 78 | 523.83 | | |

Šidák multiple comparisons test

| Comparison group 1 | Comparison group 2 | Predicted (LS) mean diff. | 95.00% CI of diff. | Adjusted *p* Value |
|---|---|---|---|---|
| Ctrl, 2 dpf | Tau, 2 dpf | -12.25 | -29.09 to 4.59 | 0.000146835705452 |
| Ctrl, 3 dpf | Tau, 3 dpf | -46.81 | -62.72 to -30.90 | 0.000000000000087 |
| Ctrl, 4 dpf | Tau, 4 dpf | -79.61 | -95.25 to -63.96 | 0.000000000459424 |
| Ctrl, 5 dpf | Tau, 5 dpf | -65.07 | -81.50 to -48.64 | 0.004836978515467 |
| Ctrl, 6 dpf | Tau, 6 dpf | -51.60 | -66.04 to -37.16 | 0.120399415095435 |
| Ctrl, 7 dpf | Tau, 7 dpf | -25.91 | -42.69 to -9.12 | 0.999742633267315 |

**Supplementary Figure S7 – Caspase 3 cleavage by Western blot in Tau zebrafish at 3 – 5 dpf**

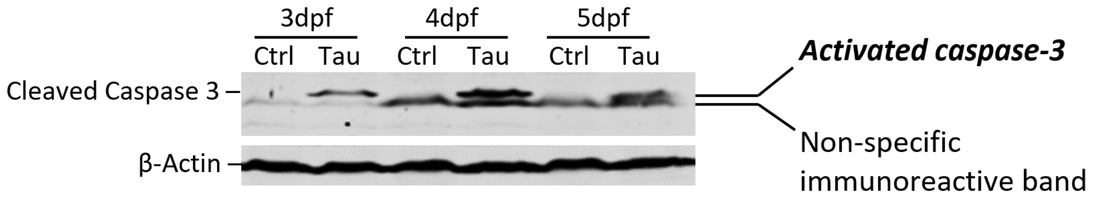


Western blots of larval zebrafish lysates at 3 – 5 dpf were probed with an antibody to cleaved (activated) Caspase-3 (upper panel) and the blot then re-probed with an antibody to β-Actin to confirm equal protein loading in all lanes (bottom panel). Lysates from Tau zebrafish at 3, 4 and 5 dpf, but not Ctrl zebrafish at the same ages, showed a prominent band at 26kDa representing activated Caspase-3. A non-specific band running immediately below activated Caspase-3 is present in every lane as indicated.

**Supplementary Figure S8 – Quantification of labeled CNS cells in zebrafish larval sections**

Since larval zebrafish brains are small, exhaustive counts of labeled cells are possible in each section. However, the brain area in each image varies according to the plane of the section, necessitating correction as follows:

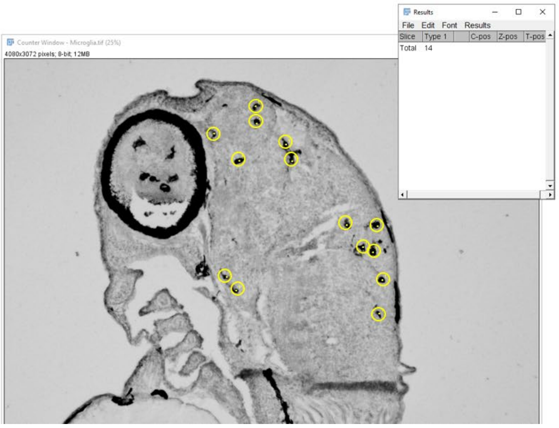


1. Count immunolabeled cells within region of interest (brain) on each section (n=14 in image shown)

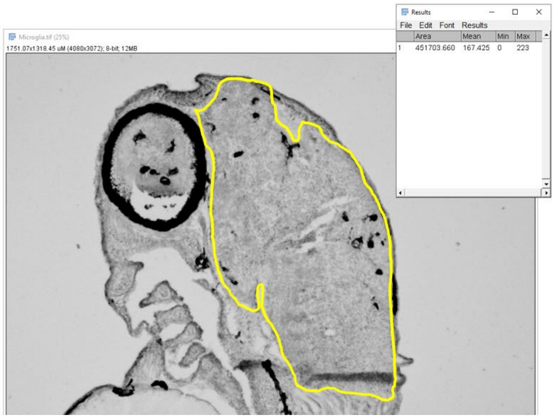


2. Determine area of region of interest on each section (= 451703.6 μm$^2$ = 0.452mm$^2$ in image shown)

3. Calculate 7.4.C4 immunolabeled cells (microglia) per unit area: 14 x 10$^6$/451703.6 = **30.99 microglia/mm$^2$** for the section shown above

4. The same analysis is completed on 10 – 12 serial sections from each zebrafish:

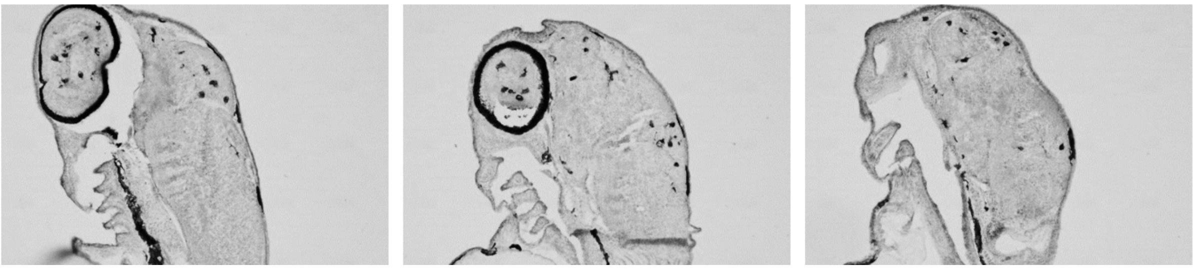

5. Mean labeling density (cell/mm$^2$) is calculated for each zebrafish.

6. Analysis is completed for mutliple zebrafish in each experimental group at each time point to arrive at data shown in Figure 2f and 2i.

7. Identical analysis is carried out in immunofluorescence sections such as TUNEL labeling in Figure 2b, 2e, except the area of interest is defined using anatomical landmarks in the DAPI labeled image, while blinded to the TUNEL image to increase rigor. The mask is then transferred to the TUNEL image for manual counting:

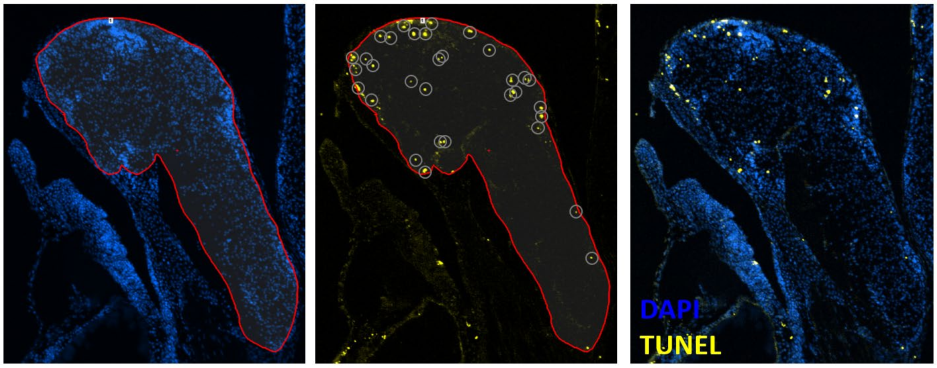


Area (DAPI) Cells (TUNEL) Merged image

**Supplementary Table 9 – Exact *p*-values for Figure 2g**

| Panel | Statistical test | Comparison group 1 | Comparison group 2 | *p* value |
|---|---|---|---|---|
| | | | | |
| 2g | One sample t-test comparing normalized Tau value to 1 | Ctrl = 1 | Tau TH | 0.002601978242698 |
| | | Ctrl = 1 | Tau GAD | 0.006680996854756 |
| | | Ctrl = 1 | Tau SYP | 0.005405732187820 |
| | | Ctrl = 1 | Tau PSD95 | 0.004708719289634 |

**Supplementary Table 10 – ANOVA summary for Figure 2i (7.4.C4$^{+}$ microglia)**

2-way ANOVA (genotype, age) table:

| Source of Variation | % of total variation | *p* |
|---|---|---|
| *Interaction* | 7.85 | 0.000000103003859 |
| *Age* | 41.53 | <0.000000000000001 |
| *Genotype (Ctrl versus Tau)* | 19.08 | <0.000000000000001 |

| | SS (Type III) | DF | MS | F (DFn, DFd) | *p* |
|---|---|---|---|---|---|
| *Interaction* | 1883.67 | 5 | 376.73 | F (5, 114) = 9.66 | P=0.000000103003859 |
| *Age* | 9959.19 | 5 | 1991.84 | F (5, 114) = 51.10 | P<0.000000000000001 |
| *Genotype* | 4576.22 | 1 | 4576.22 | F (1, 114) = 117.40 | P<0.000000000000001 |
| *Residual* | 4443.66 | 114 | 38.98 | | |

Šidák multiple comparisons test

| Comparison group 1 | Comparison group 2 | Predicted (LS) mean diff. | 95.00% CI of diff. | Adjusted *p* Value |
|---|---|---|---|---|
| Ctrl, 2 dpf | Tau, 2 dpf | -1.05 | -12.87 to 10.77 | 0.999956229578887 |
| Ctrl, 3 dpf | Tau, 3 dpf | -8.02 | -15.18 to -0.87 | 0.019633861731373 |
| Ctrl, 4 dpf | Tau, 4 dpf | -24.34 | -30.78 to -17.90 | <0.000000000000001 |
| Ctrl, 5 dpf | Tau, 5 dpf | -23.29 | -29.83 to -16.74 | 0.000000000000002 |
| Ctrl, 6 dpf | Tau, 6 dpf | -17.34 | -23.55 to -11.13 | 0.000000000103689 |
| Ctrl, 7 dpf | Tau, 7 dpf | -6.93 | -16.23 to 2.37 | 0.257070044455303 |

**Supplementary Figure S9 – No evidence that human Tau is transferred to cells not expressing the transgene**

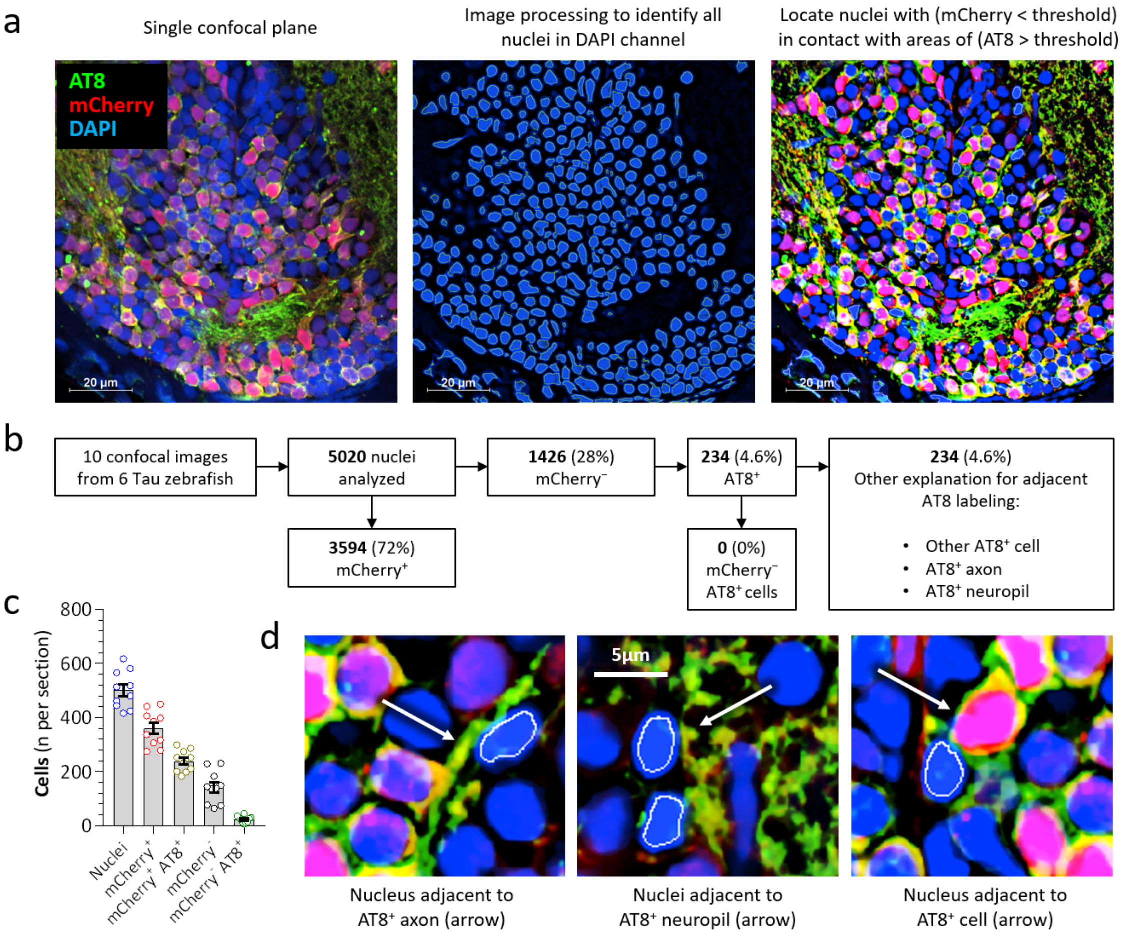


We examined Tau zebrafish brain sections for evidence that human Tau is taken up by cells adjacent to transgene-expressing neurons. An automated image analysis workflow identified candidate Tau-recipient cells (defined by AT8 immunoreactivity adjacent to nuclei lacking mCherry expression) for further manual analysis.

a: Image analysis workflow. The left panel shows a single confocal plane with three channels (DAPI, nuclei, blue; AT8, human [pS202, pT205]-Tau, green; mCherry, transgene expression, red) overlaid. The middle panel shows all nuclei identified in the DAPI channel after applying local contrast enhancement, thresholding, boundary smoothing and separation. The right image shows the processed channels overlaid; nuclei that did not express mCherry but were in contact with areas of AT8 immunoreactivity are outlined in white.

b, c: The algorithm identified 5020 nuclei in 10 confocal planes from 6 Tau zebrafish, of which 3594 (72%) were within neurons expressing the mCherry transgene. Of the remaining 1426 mCherry$^-$ (non-expressing) nuclei, 234 were identified as being of potential interest by proximity to areas of AT8 labeling and were evaluated manually. All 234 mCherry$^-$ nuclei were adjacent to AT8-immunoreactive structures in other cells, such as a neighboring cell body, axon, or neuropil. No definite AT8 immunoreactivity was found within mCherry$^-$ cells. The graph in panel c shows these data by section (data points) with the group mean ± SE.

d: Enlarged regions from panel (a) showing nuclei of cells identified as mCherry$^-$/AT8$^+$ by the algorithm (outlined in white) but found to show AT8 signal in an adjacent axon (left), neuropil (center), or cell body (right).

These data suggest that cell-to-cell transfer of human Tau in this model occurs rarely, or at quantities below threshold levels for detection, or does not occur. These experiments do not exclude human Tau seeds templating the misfolding of zebrafish Tau in adjacent cells, detection of which would require antibodies to misfolded zebrafish Tau paralogues.

**Supplementary Figure S10 – Serial extraction of Tau zebrafish in RIPA then DIGE**

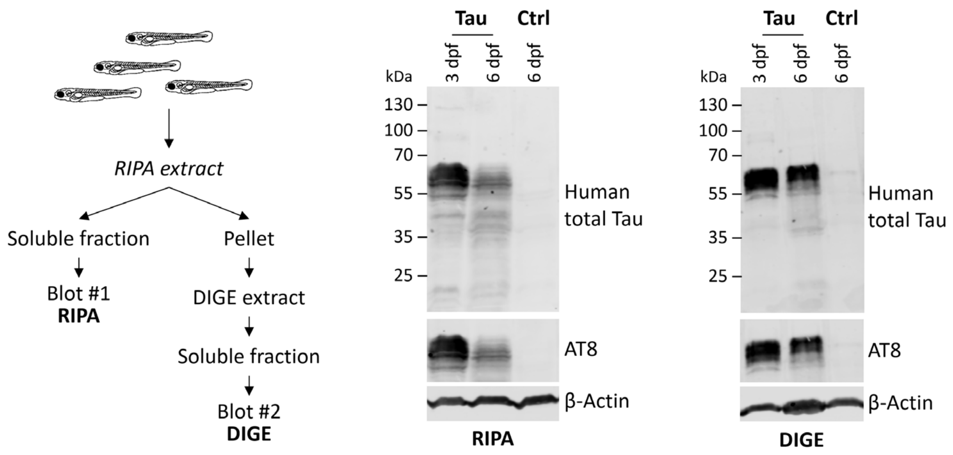


The blots in Figure 4b show separate zebrafish samples extracted using either RIPA or DIGE buffers in parallel. In this figure, the same zebrafish samples were extracted serially using RIPA first, and then the RIPA-insoluble pellet was re-extracted with DIGE, as summarized in the schematic to the left. The two western blots show the RIPA- (left) and DIGE- (right) extracted samples for 3dpf and 6dpf Tau zebrafish and 6dpf Ctrl zebrafish, probed with antibodies to human total Tau (top), human Phospho[S202, T205]-Tau (AT8; middle) and β-Actin (bottom).

This procedure is technically challenging in zebrafish as the starting material is small. However, the large reduction in Tau immunoreactivity in the RIPA-extracted samples between 3 dpf and 6 dpf is much less prominent in the DIGE-extracted samples. Together with the blots shown in Figure 4a – 4b, these data support the interpretation that human 0N/4R-Tau becomes less soluble in Tau zebrafish between 3dpf and 6dpf.

**Supplementary Figure S11 – Relative expression of human MAPT mRNA during development in transgenic Tau zebrafish**

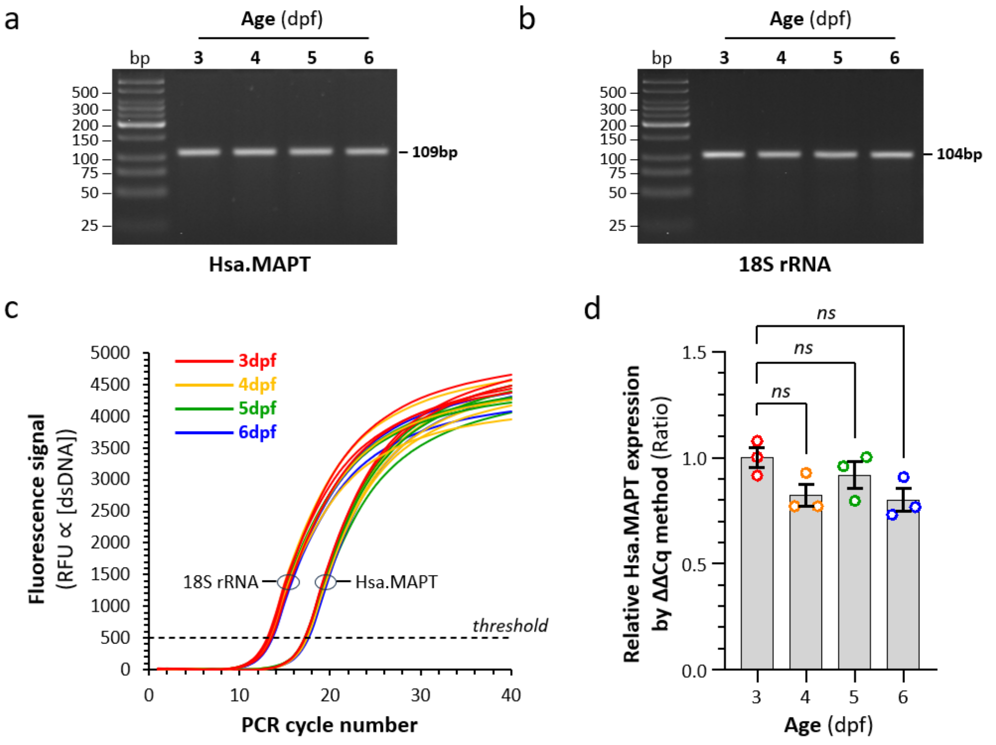


We employed real-time qRT-PCR to determine whether expression of the Tau transgene changes during development. Since expression of the common reference genes *bact1* (decrease) and *gapdh* (increase) change significantly during zebrafish development (not shown) we instead used a primer pair for 18S ribosomal RNA as a reference gene, providing an internal control for total RNA (see Supplementary Figure S3 and Supplementary table 1 for validation of the 18S-rRNA primer pair in this assay).

a, b: 25-cycle PCR amplification of first-strand cDNA from Tau zebrafish at 3-, 4-, 5- and 6-days post-fertilization (dpf) using primers to (a) Hsa.MAPT or (b) 18S rRNA. The PCR products were separated in a 4% LMP agarose gel. The images show the ethidium-stained gel photographed under UV illumination. A single product of expected size was generated by each primer pair at every developmental point. These data validate using these primers for detecting transgene expression and rRNA in Tau zebrafish and show that Hsa.MAPT transgene mRNA is expressed at all time points 3 – 6 dpf.

c: Real-time quantitative PCR amplification of first-strand cDNA from Tau zebrafish at 3 – 6 dpf using primers for 18S rRNA and the human Tau transgene. Three technical replicates were completed for each of three biological replicates (i.e. three different first-strand cDNA libraries derived from different zebrafish at each time point 3 – 6 dpf). The figure shows superimposed amplification curves for all 3 technical replicates at all four time points for one biological replicate (time points are color coded according to the legend). The curves within each set cross the quantification threshold at near-identical *x*-coordinates, suggesting abundance of RNA for both genes is similar in each of the samples.

d: Expression of the MAPT transgene relative to expression at 3dpf using the ΔΔCq method. Each data point shows the mean value of three technical replicates for each biological replicate. Bars show group mean ± SE. Data were analyzed using 1-way ANOVA with Dunnett's multiple comparisons to test to compare Hsa.MAPT cDNA abundance at each time point to 3pdf. Although a small (≈15%) decrease was seen in Hsa.MAPT between 3 and 6dpf, this was not statistically significant and may be accounted for by assay variability. This panel is identical to Figure 4c and is reproduced here for clarity.

**Supplementary Table 11 – Šidák multiple comparisons test for Figure 5b**

N1 = 48  N2 = 48  Df = 2,820

| Time bin | Mean difference | 95.00% CI of difference | Adjusted *p* value | *t* |
|---|---|---|---|---|
| 1 (0 ≤ *t* ≤ 2) | 1.20706 | 0.427597 to 1.98652 | 0.000036738918828 | 4.86218 |
| 3 | 1.47632 | 0.696864 to 2.25579 | 0.000000092117170 | 5.94682 |
| 5 | 1.44248 | 0.663022 to 2.22194 | 0.000000207777493 | 5.81050 |
| 7 | 1.59446 | 0.815003 to 2.37392 | 0.000000004697050 | 6.42270 |
| 9 | 1.57605 | 0.796585 to 2.35551 | 0.000000007575102 | 6.34851 |
| 11 | 1.45742 | 0.677960 to 2.23688 | 0.000000145412921 | 5.87068 |
| 13 | 1.50133 | 0.721869 to 2.28079 | 0.000000049941436 | 6.04755 |
| 15 | 1.65403 | 0.874565 to 2.43349 | 0.000000000966657 | 6.66263 |
| 17 | 1.70375 | 0.924286 to 2.48321 | 0.000000000247904 | 6.86291 |
| 19 | 1.69835 | 0.918890 to 2.47781 | 0.000000000287872 | 6.84117 |
| 21 | 1.68007 | 0.900612 to 2.45953 | 0.000000000476104 | 6.76755 |
| 23 | 1.67524 | 0.895778 to 2.45470 | 0.000000000543400 | 6.74808 |
| 25 | 1.51013 | 0.730665 to 2.28959 | 0.000000040174474 | 6.08298 |
| 27 | 1.48953 | 0.710073 to 2.26899 | 0.000000066742357 | 6.00003 |
| 29 | 1.33722 | 0.557759 to 2.11668 | 0.000002332954064 | 5.38649 |
| 31 | 1.34872 | 0.569259 to 2.12818 | 0.000001806082129 | 5.43281 |
| 33 | 1.58773 | 0.808270 to 2.36719 | 0.000000005597149 | 6.39558 |
| 35 | 1.52918 | 0.749715 to 2.30864 | 0.000000024975194 | 6.15972 |
| 37 | 1.42203 | 0.642567 to 2.20149 | 0.000000336853786 | 5.72811 |
| 39 | 1.29875 | 0.519292 to 2.07821 | 0.000005412699527 | 5.23154 |
| 41 | 1.11090 | 0.331437 to 1.89036 | 0.000238467585333 | 4.47484 |
| 43 | 1.26937 | 0.489913 to 2.04883 | 0.000010138302967 | 5.11320 |
| 45 | 1.33415 | 0.554692 to 2.11361 | 0.000002496948210 | 5.37414 |
| 47 | 1.29290 | 0.513441 to 2.07236 | 0.000006139794112 | 5.20797 |
| 49 | 1.28724 | 0.507778 to 2.06670 | 0.000006932828705 | 5.18516 |
| 51 | 1.38607 | 0.606610 to 2.16553 | 0.000000775541793 | 5.58327 |
| 53 | 1.38548 | 0.606024 to 2.16495 | 0.000000786022282 | 5.58091 |
| 55 | 1.66705 | 0.887586 to 2.44651 | 0.000000000679326 | 6.71508 |
| 57 | 1.73399 | 0.954530 to 2.51345 | 0.000000000106378 | 6.98474 |
| 59 | 1.70589 | 0.926433 to 2.48535 | 0.000000000233561 | 6.87156 |

The movement traces in Figure 5b were analyzed by calculating the mean swimming speed for each of the 48 zebrafish in each group, within contiguous 2-minute time bins spanning the 60 minutes of recording. For each time bin, Tau and Ctrl group means were compared using 2-way repeated-measures ANOVA (experimental group, time point) with Šidák multiple comparisons test. The adjusted *p* value is shown for each time bin.

**Supplementary Figure S12 – Biological replicate motor activity assays comparing Tau and Ctrl zebrafish**

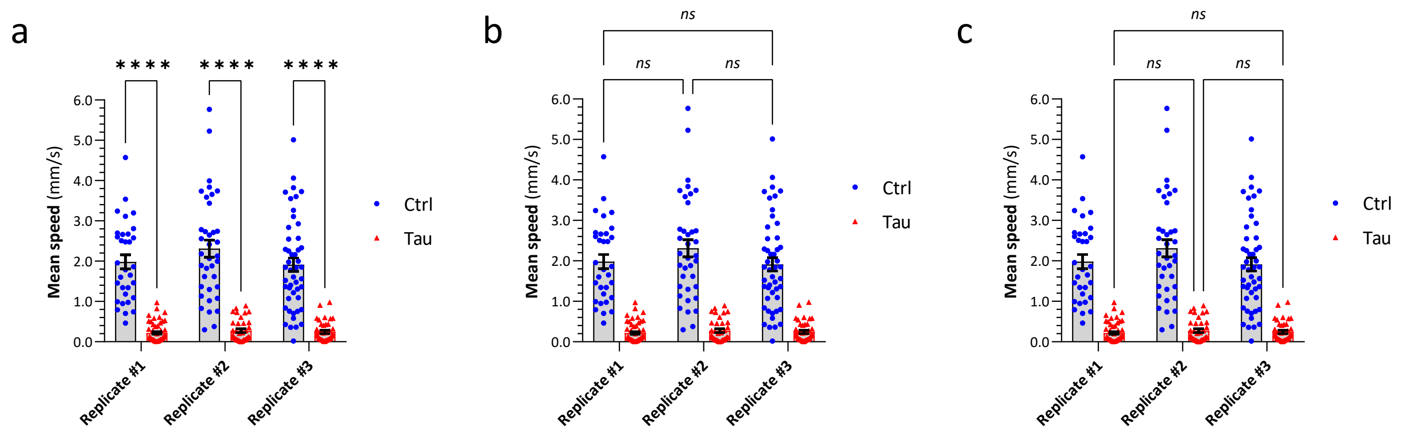


3 biological replicate experiments (same transgenic allele, different parents, crossed different days) comparing the mean speed of Tau and Ctrl zebrafish over 60 minutes of spontaneous swimming in bright white ambient illumination at 5 dpf. Data points show individual zebrafish, bars show group mean ± SE. Experimental groups and replicates were compared using 2-way ANOVA (genotype, replicate) with a single Tukey multiple comparisons test (see Supplementary Table S12). The same data are shown three times for clarity.

a: There was a robust difference between Tau and Ctrl in each assay
b: There was no difference between assays for Ctrl
c: There was no difference between assays for Tau

Given the absence of significant differences between the assays, it was admissible to combine these data to generate Figures 5c – 5g.

**Supplementary Table 12 – ANOVA summary for Supplementary Figure S12**

Details of the 2-way ANOVA (genotype, replicate) used in Supplemental Figure S12 to compare Ctrl and Tau zebrafish in 3 biological replicate assays.

| Source of Variation | % of total variation | *P* |
|---|---|---|
| *Interaction* | 0.3809 | 0.350146138610801 |
| *Experimental replicate* | 0.5807 | 0.202605208323934 |
| *Ctrl versus Tau* | 54.44 | <0.000000000000001 |

| | SS (Type III) | DF | MS | F (DFn, DFd) | *P* |
|---|---|---|---|---|---|
| *Interaction* | 1.395 | 2 | 0.6976 | F (2, 241) = 1.054 | P=0.350146138610801 |
| *Experimental replicate* | 2.127 | 2 | 1.064 | F (2, 241) = 1.607 | P=0.202605208323934 |
| *Ctrl vs Tau* | 199.4 | 1 | 199.4 | F (1, 241) = 301.3 | P<0.000000000000001 |
| *Residual* | 159.5 | 241 | 0.6619 | | |

Tukey multiple comparisons test

| | Comparison | Predicted (LS) mean diff. | 95.00% CI of diff. | Adjusted P Value |
|---|---|---|---|---|
| a: Ctrl vs. Tau each replicate | Replicate #1:Ctrl vs. Replicate #1:Tau | 1.765 | 1.406 to 2.124 | <0.000000000000001 |
| | Replicate #2:Ctrl vs. Replicate #2:Tau | 2.036 | 1.661 to 2.411 | <0.000000000000001 |
| | Replicate #3:Ctrl vs. Replicate #3:Tau | 1.671 | 1.331 to 2.012 | <0.000000000000001 |
| b: Compare Ctrl between replicates | Replicate #1:Ctrl vs. Replicate #2:Ctrl | -0.3263 | -0.7895 to 0.1368 | 0.222191178051007 |
| | Replicate #1:Ctrl vs. Replicate #3:Ctrl | 0.07036 | -0.3675 to 0.5082 | 0.923925766974799 |
| | Replicate #2:Ctrl vs. Replicate #3:Ctrl | 0.3967 | -0.02304 to 0.8164 | 0.068410230248918 |
| c: Compare Tau between replicates | Replicate #1:Tau vs. Replicate #2:Tau | -0.05549 | -0.4699 to 0.3589 | 0.946530094330830 |
| | Replicate #2:Tau vs. Replicate #3:Tau | -0.02320 | -0.4222 to 0.3758 | 0.989693422254547 |
| | Replicate #1:Tau vs. Replicate #3:Tau | 0.03229 | -0.4059 to 0.4705 | 0.983492703865083 |

A single Tukey test was completed to encompass all 9 comparisons, but data are shown in three groups of 3 for clarity.

**Supplementary Figure S13 – Three different control groups behave similarly in motor activity assays**

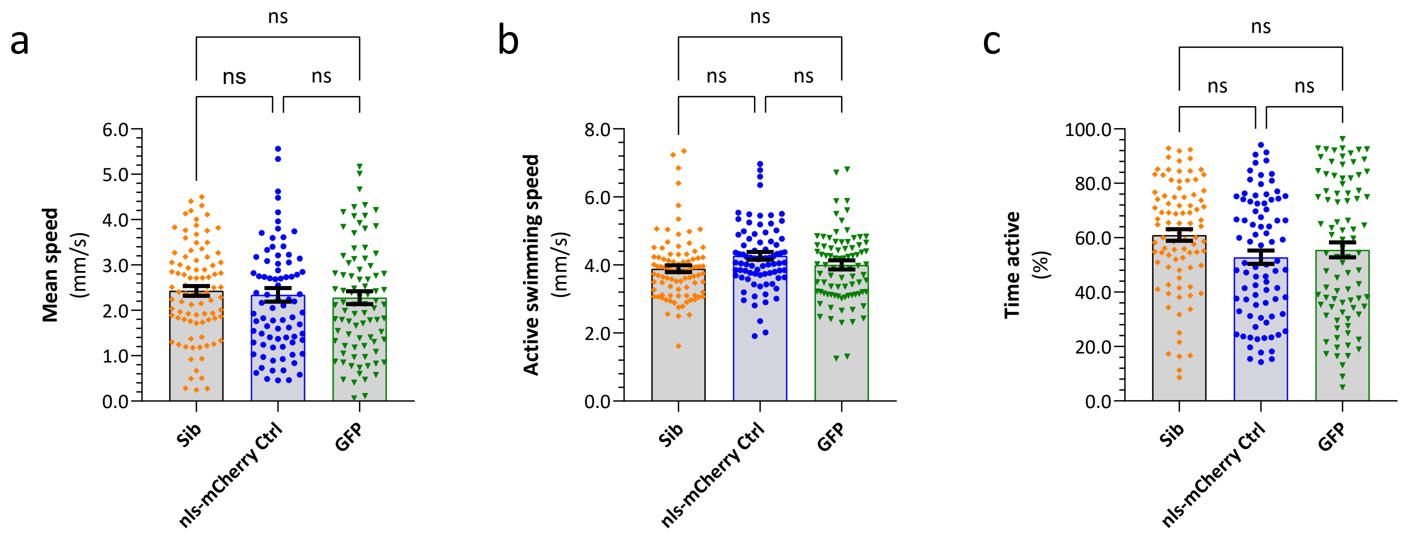


In Figure 5, we show data comparing Tau and Ctrl zebrafish, which contain exactly the same complement of transgenes, with the exception that human Tau is not expressed in Ctrl zebrafish. However, since there is substantial genetic heterogeneity in WT zebrafish that can influence outcomes in neurological assays, the possibility that some of the phenotypes are caused by genetic variation unrelated to the transgene should be considered.

To address this question, we tested two additional controls in this assay: (i) Siblings of Tau zebrafish that do not show mCherry fluorescence and therefore do not express human Tau (Sib; orange). These are a mixture of WT, driver and responder zebrafish that share background genetics with their Tau siblings. (ii) Tg(*elavl3:gal4-vp16*); Tg(*UAS:egfp*) zebrafish (GFP; green) that express a second responder allele unrelated to the Tg(*UAS:2a-nls-mCherry*) allele present in Ctrl zebrafish.

Motor activity assays were run and analyzed identically to those shown in Figure 5[2,3]. The graphs show:

a: Mean speed
b: Active swimming speed
c: % time active

In each graph, data points show individual zebrafish, bars show mean ± SE. Comparison between groups was carried out using 1-way ANOVA with Tukey multiple comparison test.

There were no statistically significant differences between the groups. Together with direct comparisons between Tau zebrafish and each of these controls, and replicate phenotypes in multiple transgenic Tau lines, these data strongly support our interpretation that the observed phenotypes in Figure 5 are attributable to expression of human 0N/4R-Tau. These data also support the use of these controls interchangeably in subsequent experiments.

**Supplementary Figure S14 – Heterologous protein over-expression does not alone cause motor phenotypes**

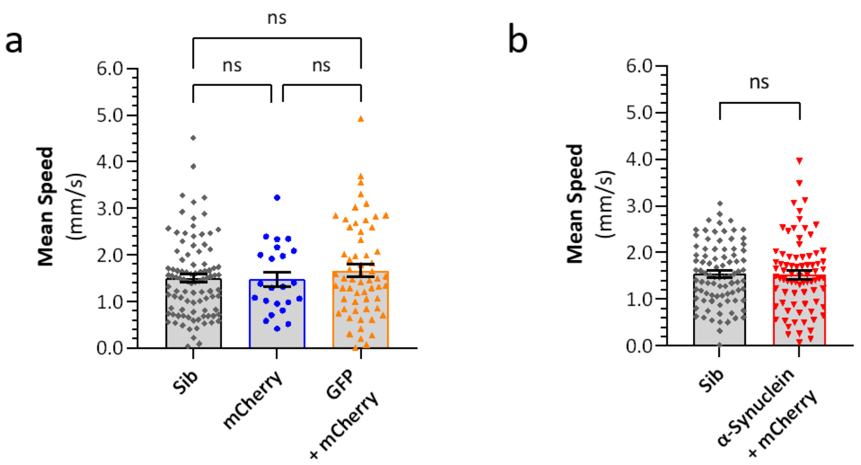


In Figure 5, we show data comparing Tau and Ctrl zebrafish, which contain exactly the same complement of transgenes, with the exception that human Tau is not expressed in Ctrl zebrafish. However, this means that Ctrl zebrafish express one less transgene than Tau zebrafish, and so these experiments do not fully exclude the possibility that phenotypes in Tau zebrafish are non-specific and arise from over-expression of multiple heterologous transgenes. To address this question, we analyzed two additional models that express the same number of transgenes as Tau zebrafish, using the same 96-well plate motor function assay. In each graph, data points show individual zebrafish, bars show mean ± SE (data in each panel are combined from biological replicates that did not differ significantly from each other, exactly as analyzed in Supplementary Figure 12).

a. Tg(*elavl3:gal4-vp16*); Tg(*UAS:egfp*); Tg(*UAS:p2a-nls-mCherry*) zebrafish (orange) express both GFP and mCherry simultaneously, replicating the same number of heterologous genes expressed under the same driver as Tau zebrafish. Control groups were siblings with mCherry fluorescence only (blue), and siblings lacking both mCherry and GFP fluorescence (grey). Comparison between groups was carried out using 1-way ANOVA with Tukey's multiple comparison test.

b. Tg(*elavl3:gal4-vp16*); Tg(*UAS:Hsa.SNCA-p2a-nls-mCherry*)$^{pt423}$ zebrafish express the same bicistronic responder cassette under the same driver as Tau zebrafish, but with human WT α-Synuclein cDNA replacing the human 0N/4R-Tau coding sequence. This replicates the expression system of the Tau model exactly, and also expresses an independent heterologous human protein. Controls were siblings lacking mCherry expression. Comparison between groups was carried out using a 2-tailed *t*-test with Welch's correction for unequal variance.

There were no statistically significant differences between the groups in either set of assays, showing that expressing mCherry with a second transgene (either GFP or human α-Synuclein) under the same pan-neuronal driver does not result in a motor phenotype. These data do not support the interpretation that the hypokinetic motor phenotype of Tau zebrafish is a non-specific artifact of heterologous protein over-expression. Instead, this phenotype likely reflects a pathogenic property of human 0N/4R-Tau in this model.

**Supplementary Figure S15 – Methodology and abnormalities of ocular movement in Tau zebrafish**

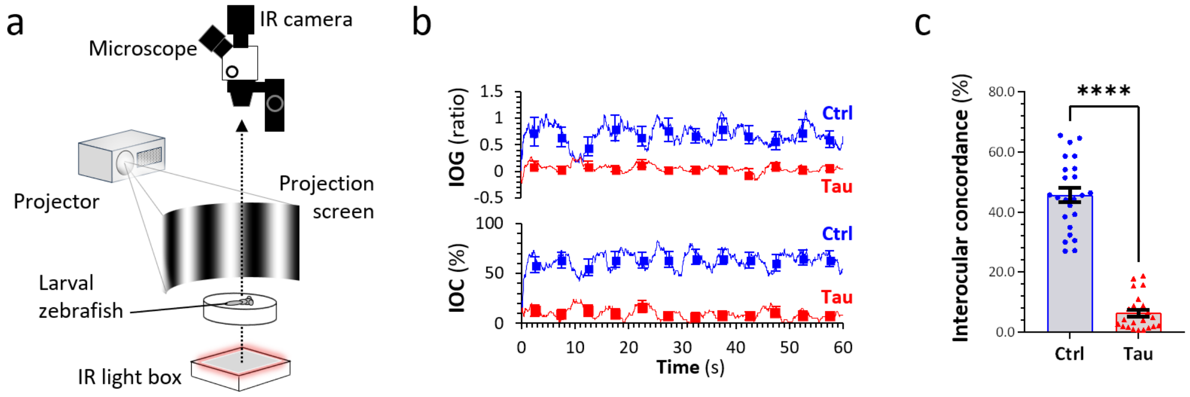


a: Illustration of the experimental configuration[4] used to elicit optokinetic reflexes in Figure 6. Note that the visual stimulus is presented to the left (stimulated) eye only.

b: Unilateral stimulus presentation in this assay allows analysis of the coordination between L and R eyes during the response. These graphs show how interocular gain (IOG; the ratio of change in angle of stimulated eye to change in angle of contralateral eye at each frame transition; see also Figure 6h) and interocular concordance (IOC; the proportion of frame transitions at which there was movement of both eyes in the same direction) vary in Ctrl (blue) and Tau (red) zebrafish during the same example segment of recording shown in Figure 6b.

c: Quantitative analysis of mean IOC for Ctrl (blue) and Tau (red) zebrafish. Data points show individual zebrafish, bars show mean ± SE. ****$p$<0.0001, Ctrl *vs*. Tau, 2-tailed unpaired $t$-test with Welch's correction for unequal variance.

**Supplementary Figure S16 – Abnormalities of the visual motor response in Tau zebrafish**

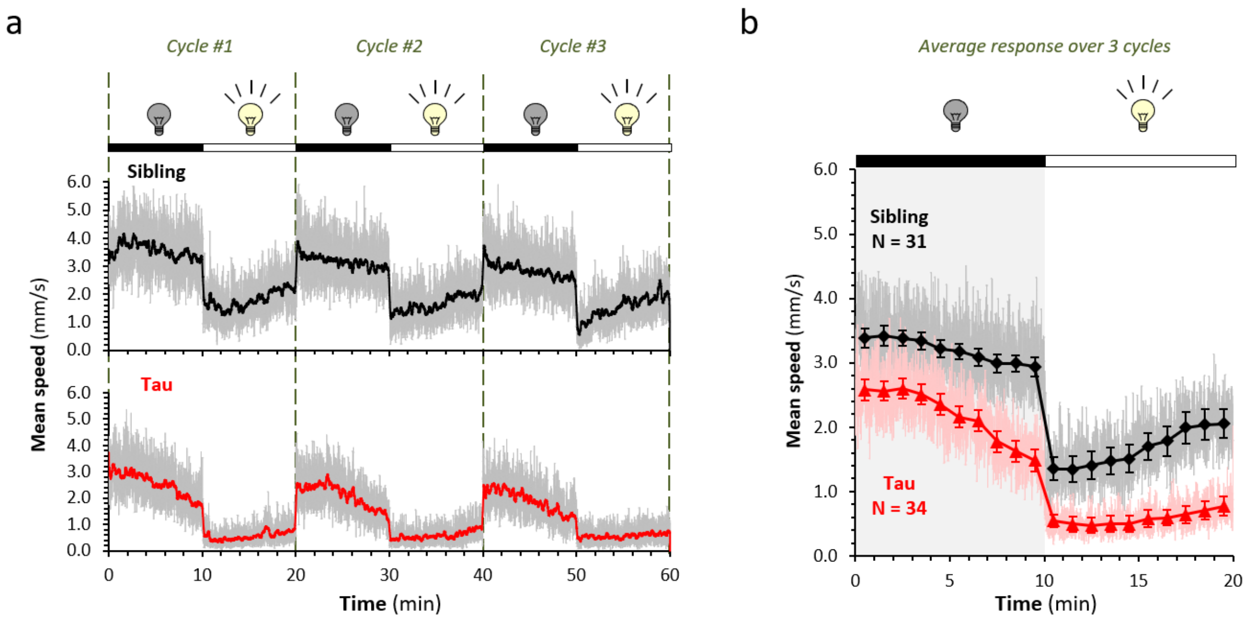


In order to optimize an assay of motor activity for high-throughput screening, it was necessary to reduce variability as far as possible. This was achieved by using the zebrafish responses to alternating periods of bright ambient light and darkness, as evoked responses tend to be less variable than spontaneous behavior, and responses to multiple stimuli can be averaged. In addition, to simplify the breeding scheme for executing a screen, and further reduce variability by ensuring the controls shared similar genetic background and were at exactly the same developmental age as Tau zebrafish, the assay was optimized using non-expressing siblings as controls. This is valid because siblings showed identical responses to other controls in all assays tested (Supplementary Figure S13).

a: Motor responses for 31 Sib and 34 Tau zebrafish over three consecutive stimulus cycles of [10 minutes darkness + 10 minutes light] as shown above the graph. The gray traces show mean group centroid displacement at each video frame transition scaled to show mean speed (y-axis); the colored lines (Sib, black; Tau, red) show the same data averaged over a 50-frame moving window.

b: The three stimulus cycles were averaged to provide mean activity waveforms for each individual zebrafish over a single cycle; the averaged traces for each experimental group are shown (gray). The mean speed for each zebrafish was calculated in contiguous 1-minute time bins over the 20-minute averaged stimulus cycle. This allowed calculation of group mean speed (solid markers) and SE (error bars) in each time bin across the averaged response cycle.

These analyses reduced response variability and increased statistical power to detect differences between smaller samples of zebrafish in each group, providing an essential basis for the screening assays shown in Figure 7. Since the difference between Ctrl and Tau was proportionately greater in the light phase of the response, and this was more quantitatively reproducible between biological replicates than the dark phase abnormalities, we used VMR light phase swimming speed as the readout for the screen.

**Supplementary Table 13 – Šidák multiple comparisons table for Supplementary Figure S16b**

| Time (Min) | Predicted (LS) mean diff. | 95.00% CI of diff. | Adjusted P Value |
|---|---|---|---|
| 0.5 (0 ≤ t <1 ) | 0.806912641366225 | 0.110909956833643 to 1.50291532589881 | 0.009458477660351 |
| 1.5 | 0.856180163187854 | 0.160177478655273 to 1.55218284772044 | 0.004191672094418 |
| 2.5 | 0.783846788425048 | 0.0878441038924668 to 1.47984947295763 | 0.013636095234470 |
| 3.5 | 0.832441664136623 | 0.136438979604041 to 1.52844434866920 | 0.006238104819818 |
| 4.5 | 0.868476591081594 | 0.172473906549012 to 1.56447927561418 | 0.003398073653293 |
| 5.5 | 1.01484090891840 | 0.318838224385823 to 1.71084359345099 | 0.000227994715967 |
| 6.5 | 0.988876888994308 | 0.292874204461726 to 1.68487957352689 | 0.000378316585406 |
| 7.5 | 1.21731838519924 | 0.521315700666660 to 1.91332106973182 | 0.000002950694177 |
| 8.5 | 1.36357402371917 | 0.667571339186584 to 2.05957670825175 | 0.000000082594425 |
| 9.5 | 1.45304057115750 | 0.757037886624914 to 2.14904325569008 | 0.000000007755137 |
| 10.5 | 0.810600031309298 | 0.114597346776716 to 1.50660271584188 | 0.008913110573539 |
| 11.5 | 0.853784509487666 | 0.157781824955085 to 1.54978719402025 | 0.004365266187774 |
| 12.5 | 0.928790748576850 | 0.232788064044268 to 1.62479343310943 | 0.001167796835428 |
| 13.5 | 0.977701580645162 | 0.281698896112580 to 1.67370426517774 | 0.000468767742351 |
| 14.5 | 1.01941306736243 | 0.323410382829847 to 1.71541575189501 | 0.000208291705206 |
| 15.5 | 1.12485552277040 | 0.428852838237817 to 1.82085820730298 | 0.000023443366506 |
| 16.5 | 1.18968867267552 | 0.493685988142940 to 1.88569135720810 | 0.000005566046220 |
| 17.5 | 1.35094928842505 | 0.654946603892466 to 2.04695197295763 | 0.000000114080609 |
| 18.5 | 1.33412558728653 | 0.638122902753946 to 2.03012827181911 | 0.000000174705283 |
| 19.5 | 1.28679395825427 | 0.590791273721686 to 1.98279664278685 | 0.000000564786257 |

The data shown in Supplementary Figure S16b were analyzed by 2-way repeated measures ANOVA (group, time) with Šidák multiple comparisons test to evaluate the difference between Ctrl and Tau at each time point during the response. The table shows the predicted least squares difference between experimental groups in each time bin, 95% CI of the difference and adjusted *p*.

**Supplementary Table 14 – Small molecule screening data**

| Category | Parameter | Description |
|---|---|---|
| Assay | Type of assay | Machine vision analysis of zebrafish motility |
| | Target | Agnostic/phenotypic screen |
| | Primary measurement | Rescue of Tau zebrafish hypokinesia |
| | Key reagents | Tau and Sib zebrafish |
| | Assay protocol | Measurement of light phase swimming speed during the visual motor response. See *Methods*. |
| | | |
| Library | Library size | 147 small molecules |
| | Library composition | Inhibitors of epigenetic reader, writers and erasers |
| | Source | Cayman Chemical, Ann Arbor, MI |
| | | |
| Screen | Format | 96 well plate |
| | Concentration(s) tested | Maximum tolerated concentration in WT zebrafish (0.5 – 50 μM) |
| | Plate controls | Untreated Tau and Sibling zebrafish |
| | Reagent/ compound dispensing system | Manual |
| | Detection instrument and software | IR video from FL3-U3-13Y3M-C camera, Point Gray Research; video analyzed by MATLAB running *LSRtrack* |
| | Assay validation/QC | See Figure 7 and S19. QC based on untreated Tau and Sib controls in each assay. |
| | Correction factors | None |
| | Normalization | Assay output normalized to controls in each assay as shown in Figure 7d' |
| | | |
| Post-HTS analysis | Hit criteria | Library mean + 3*SD |
| | Hit rate | 3/147 |
| | Additional assay(s) | Survival analysis, cell death, transgene expression, microgliosis, synapse abundance, microglial phagocytosis of synaptic material, see Figures 8 – 9 |
| | Confirmation of hit purity and structure | Results replicated with repurchased compound |
| | | |

**Supplementary Figure S17 – Small molecule screen part 1: Determination of maximum tolerated concentration**

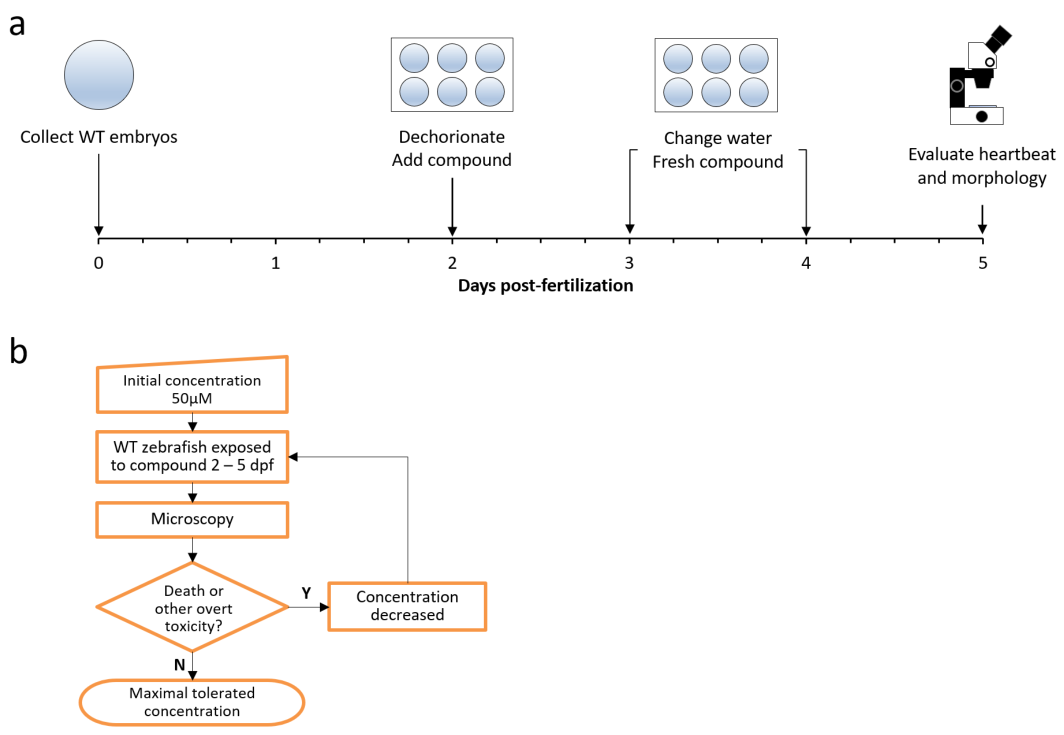


a: WT zebrafish embryos were collected after fertilization and housed in 6-well plates with daily changes of E3 buffer. The larvae were dechorionated at 2dpf and buffer replaced with fresh E3 containing library compound. The buffer was replaced with fresh E3 containing the compound at 3dpf and 4dpf, then microscopy carried out at 5dpf to evaluate for viability (heartbeat) and morphology.

b: Each compound was evaluated initially at 50µM final concentration in the buffer. If toxicity was found after exposure, the concentration was reduced. Iterative testing eventually provided the maximum tolerated concentration (MTC) for each small molecule in the library.

**Supplementary Figure S18 – Small molecule screen part 2: Rescue of motor phenotype in Tau zebrafish**

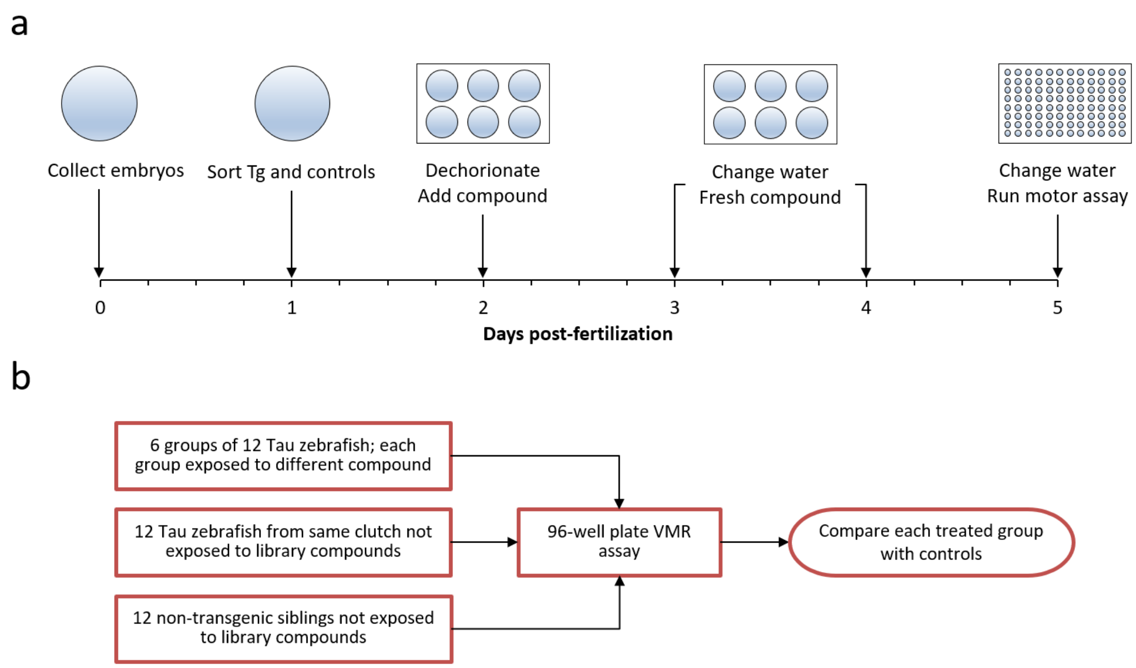


**A:** Zebrafish embryos from a Tg(*elavl3:gal4-vp16*) x Tg(*UAS:Hsa.MAPT-p2a-nls-mCherry*) cross were collected and housed in in 6-well plates with daily changes of E3 buffer. Embryos were genotyped by mCherry fluorescence at 1dpf and sorted into Tau and Sib groups. Larvae were then dechorionated at 2dpf, genotypes verified, and buffer replaced with fresh E3 containing library compound. There were 7 groups of Tau zebrafish: 6 of the groups received E3 containing a different small molecule at its MTC (as defined in part 1 of the screen), the 7$^{th}$ group received E3 only. A single group of 12 Sib zebrafish also received E3 only. Buffer was replaced with fresh E3 ± compound at 3dpf and 4dpf.

**B:** At 5dpf, compounds were washed off and replaced with fresh E3 and the VMR elicited as shown in Supplementary Figure S16 for quantitative analysis as shown in Supplementary Figure S19. The entire library was tested, 6 compounds at a time, in this way.

## Supplementary Figure S19 – Algorithm logic for analysis of video tracking data from rescue screening assays

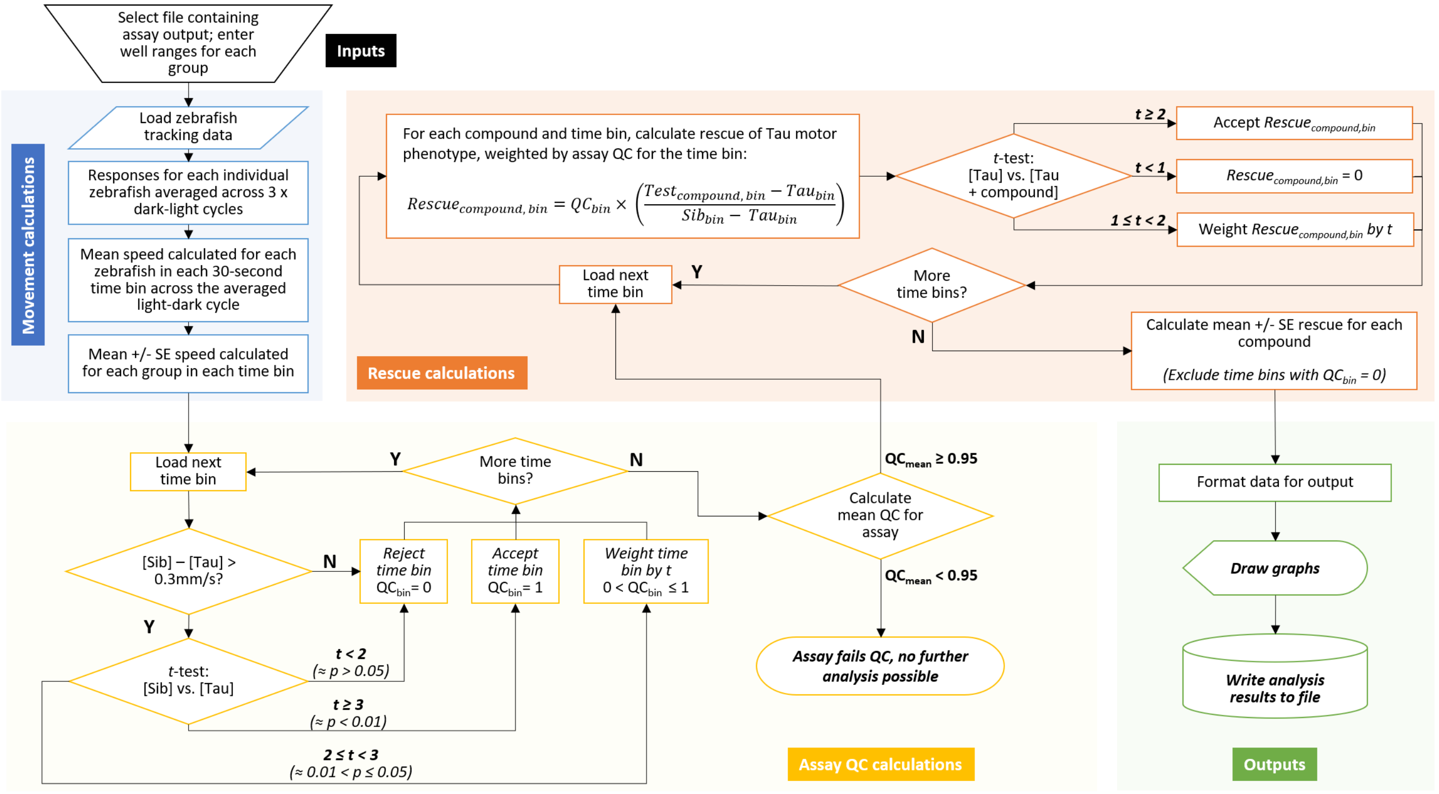


The flowchart depicts the algorithm used to calculate rescue of the abnormal VMR phenotype in Tau zebrafish by small molecule exposure. Calculations are split into 4 consecutive processes:

1. Movement calculations (blue) – individual zebrafish responses are averaged over 3 cycles of dark-light stimuli as shown in Supplementary Figure S16, then mean speed for each zebrafish calculated in contiguous 30-second time bins across the averaged stimulus cycle. Mean and SE speed are then calculated for each experimental group in each time bin across the stimulus cycle.

2. Assay quality control (QC) calculations (yellow) – the responses of untreated Tau and Sib zebrafish are compared to ensure that the phenotype in Tau zebrafish was sufficiently robust to make rescue calculations meaningful. Individual time bins where $Speed_{Sib} - Speed_{Tau} < 0.3$ mm/s are assigned QC=0 and rejected. Each accepted time bin is then analyzed to ensure that $Speed_{Sib}$ and $Speed_{Tau}$ are statistically significantly different at $p<0.01$ by *t*-test; if so, the time bin is assigned a QC score of 1 and accepted. If p > 0.05, the bin is assigned QC=0 and rejected. For $0.01<p<0.05$, QC is weighted linearly between 1 and 0. Mean QC is then calculated for the assay. If QC<0.95 the assay has failed quality control, and no further analysis is possible. This stringent QC process overall rejected < 5% of assays and ensured that data relating to phenotypic rescue were only accepted as part of the screen if the model behaved exactly as expected.

3. Rescue calculations (orange) – this calculates % rescue for each small molecule in each time bin according to the formula shown in Figure 7d' multiplied by the QC value for the bin. For each compound and each time bin, if $Speed_{Tau}$ and $Speed_{Tau+Chemical}$ are significantly different at $p<0.05$ by t-test, the value for % rescue in that time bin is accepted. If $p>0.1$, rescue is set to 0 for that time bin. For $0.1<p<0.05$, rescue is weighted by $(t-1)^3$. These stringent criteria for accepting data points minimized any contribution to overall rescue score from time bins in which effects were marginal. Mean rescue for each chemical is then calculated for the light and dark phases of the VMR, omitting any time bins that were rejected at stage 2 (QC).

4. Outputs (green) – QC metrics and binned rescue data are output graphically (see Supplementary Figure S20) and numerical data are written into a spreadsheet for downstream analysis.

**Supplementary Figure S20 – Example outputs from screening assay**

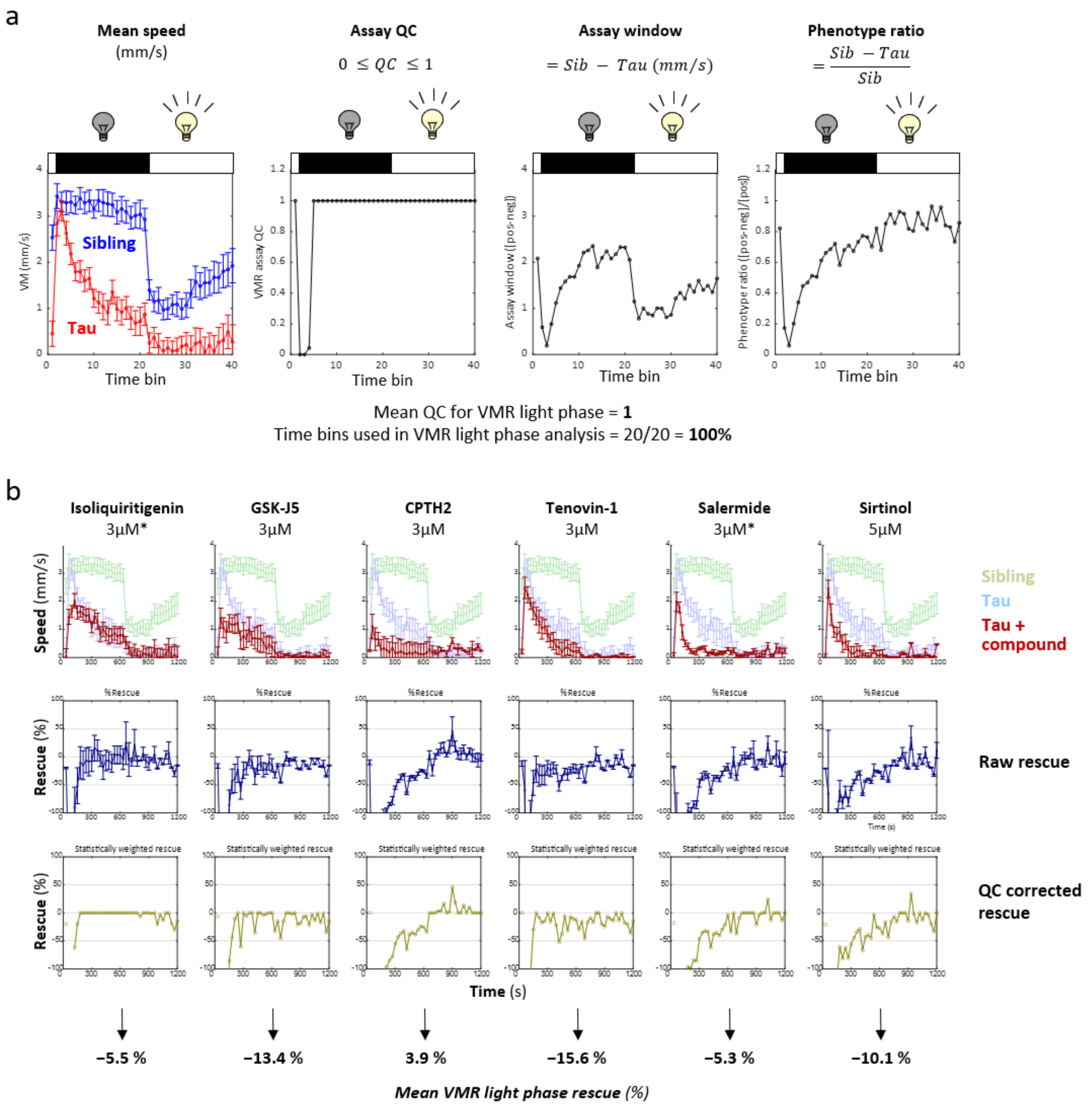


a: Mean ± SE speed of 12 Tau (red) and 12 Sib (blue) zebrafish in 30-second time bins, averaged across 3 x 20-minute dark-light stimulus cycles (first graph). There is a robust phenotype but overlap between Tau and Sib at the start of the dark phase would make rescue calculations impossible in these time bins. This is reflected in the QC score (second graph), which rejects bins 2 – 4 of the dark phase responses. The assay window (difference between Sib and Tau; third graph) and phenotype ratio (assay window as a fraction of Sib response) show the effect size attributable to Tau across the stimulus cycle. Overall, the VMR light phase in this experiment performed well as an assay to detect rescue of neurological function.

b: Automated outputs showing the effect of each of 6 compounds tested in the assay shown in panel A. In the top row, the responses of Tau zebrafish exposed to small molecules (red) are superimposed on the Tau (blue) and Sib (green) controls. None of the compounds improved the motor function of Tau zebrafish. The second and third rows show calculations for phenotypic rescue, either without (middle row) or with (bottom row) removal of points failing QC and correction for whether the effect was significant in each time bin. Compounds marked * were subsequently retested at lower concentrations.

**Supplementary Table 15 – Summary of data from the small molecule screen in Figure 7d**

Summary table of data from the epigenetic library screen. Rows show each compound, ranked by % rescue of the light phase of the VMR; hits shown in red, other compounds of potential interest shown in green. Columns (from left to right): rank; maximum tolerated concentration (i.e. concentration tested in this assay; µM); mean % rescue of the light phase of the VMR (negative values indicate that the compound worsened the motor phenotype of Tau zebrafish); SE of rescue; compound identity; molecular target.

| Rank | MTC (µM) | % Rescue | SE | Chemical | Target |
|---|---|---|---|---|---|
| **1** | **2** | **74.89** | **7.88** | **(+)-JQ1** | **BET protein bromodomains** |
| **2** | **0.3** | **66.92** | **5.11** | **Trichostatin A** | **class I, II, and IV histone deacetylases (HDACs)** |
| **3** | **6** | **55.17** | **3.92** | **2,4-DPD** | **HIF-α prolyl hydroxylase** |
| **4** | **5** | **46.32** | **6.53** | **Valproic Acid (sodium salt)** | **class I histone deacetylases (HDACs)** |
| **5** | **25** | **40.51** | **3.61** | **HC Toxin** | **histone deacetylases (HDACs)** |
| 6 | 50 | 30.75 | 4.17 | 5-Methyl-2'-deoxycytidine | DNA methylation |
| 7 | 50 | 30.7 | 4.16 | PFI-3 | SMARCA4 bromodomain and PB1(bromodomain 5) |
| 8 | 10 | 29.55 | 9.6 | Oxamflatin | histone deacetylases (HDACs) |
| 9 | 50 | 28.29 | 6.96 | Chidamide | histone deacetylases (HDACs) |
| 10 | 37.5 | 26.39 | 6.14 | SAHA-BPyne | class I and class II histone deacetylases (HDACs) |
| 11 | 50 | 25.92 | 4.55 | N-Oxalylglycine | JMJD2A, JMJD2C, and JMJD2E histone demethylases |
| 12 | 25 | 25.03 | 5.93 | 4-pentynoyl-Coenzyme A (trifluoroacetate salt) | lysine acetyltransferases |
| 13 | 50 | 24.16 | 4.86 | 2',3',5'-triacetyl-5-Azacytidine | DNA methyltransferase |
| 14 | 20 | 23.8 | 3.9 | S-(5'-Adenosyl)-L-methionine (tosylate) | Methyl donor |
| 15 | 0.75 | 23.59 | 4.19 | Lestaurtinib | JAK2, FLT3, TrkA |
| 16 | 30 | 22.39 | 4.07 | S-(5'-Adenosyl)-L-methionine chloride (hydrochloride) | Methyl donor |
| 17 | 50 | 20.67 | 5.09 | BML-210 | histone deacetylases (HDACs) |
| 18 | 50 | 20.29 | 4.2 | Tenovin-6 | p53 activator |
| 19 | 50 | 20.2 | 3.03 | Butyrolactone 3 | histone acetyltransferase Gcn5 |
| 20 | 50 | 19.96 | 10.06 | BIX01294 (hydrochloride hydrate) | G9a histone methyltransferase |
| 21 | 40 | 19.78 | 4.42 | CAY10398 | histone deacetylase (HDAC1) |
| 22 | 40 | 19.11 | 4.24 | trans-Resveratrol | SIRT1 activator, multiple actions |
| 23 | 20 | 19.1 | 3.43 | Bromosporine | Bromodomain proteins |
| 24 | 1.5 | 17.81 | 1.64 | Isoliquiritigenin | Guanyl cyclase activator |
| 25 | 40 | 17.39 | 5.69 | Ellagic Acid | coactivator-associated arginine methyltransferase 1 |
| 26 | 20 | 17.31 | 3.7 | OTX015 | BRD2, 3 and 4 |
| 27 | 50 | 16.95 | 7.67 | EPZ5676 | DOT1L histone methyltransferase |
| 28 | 1 | 16.62 | 5.1 | 3,3'-Diindolylmethane | histone deacetylases (HDACs) and DNA MTs |
| 29 | 50 | 15.46 | 2.85 | Sodium Butyrate | histone deacetylases (HDACs) |
| 30 | 12.5 | 13.96 | 4.96 | Chaetocin | HMTs |
| 31 | 50 | 13.5 | 7.93 | I-BET151 | BRD2, 3, 4 |
| 32 | 20 | 12.58 | 5.2 | MC 1568 | Class Iia HDACs |

| | | | | | |
|---|---|---|---|---|---|
| 33 | 1.5 | 12.51 | 7.63 | SGI-1027 | DNA MTs |
| 34 | 50 | 12.27 | 2.89 | UNC0224 | G9a histone methyltransferase |
| 35 | 50 | 11.22 | 3 | GSK-J1 (sodium salt) | H3K27 histone demethylase JMJD3 |
| 36 | 50 | 11.11 | 4.45 | RG-108 | DNA MTs |
| 37 | 50 | 10.7 | 4.44 | Tubastatin A | HDAC6 |
| 38 | 50 | 9.6 | 2.21 | Suramin (sodium salt) | Topoisomerase II |
| 39 | 50 | 9.33 | 4.54 | 2,4-Pyridinedicarboxylic Acid | Jumonji HDMs |
| 40 | 30 | 8.99 | 6.25 | Zebularine | DNA MTs |
| 41 | 50 | 8.78 | 4.12 | Etoposide | Topoisomerase II |
| 42 | 50 | 8.4 | 2.21 | I-BET762 | BET proteins |
| 43 | 20 | 8.08 | 6.79 | Anacardic Acid | HAT activity P300 and PCAF |
| 44 | 50 | 8.05 | 7.04 | Rucaparib (phosphate) | PARP1 |
| 45 | 50 | 7.83 | 2.5 | (+)-Abscisic Acid | Chromatin structure gene expression |
| 46 | 12.5 | 7.63 | 7.12 | AK-7 | SIRT2 |
| 47 | 50 | 7.51 | 2.43 | AGK2 | SIRT2 |
| 48 | 50 | 6.7 | 1.76 | Daminozide | HDMs KDM2A, PHF8, KDM7A |
| 49 | 50 | 6.34 | 1.73 | (R)-PFI-2 (hydrochloride) | SET7/9 HMT |
| **50** | **6** | **6.26** | **5.42** | **(−)-JQ1** | **Negative control for (+)-JQ1** |
| 51 | 0.7 | 6.12 | 5.07 | CAY10669 | HAT PCAF |
| 52 | 50 | 5.9 | 5.14 | CPI-203 | BRD4 |
| 53 | 1.5 | 4.93 | 2.37 | IOX1 | JMJ2DA HDM |
| 54 | 50 | 4.37 | 2.28 | Decitabine | DNA MTs |
| 55 | 50 | 4.19 | 1.68 | LAQ824 | HDACs |
| 56 | 50 | 3.9 | 1.58 | AMI-1 (sodium salt) | Arginine methyltransferases |
| 57 | 3 | 3.89 | 3.19 | CPTH2 (hydrochloride) | histone acetyltransferase Gcn5 |
| 58 | 50 | 3.43 | 1.63 | I-CBP112 (hydrochloride) | CBP EP300 |
| 59 | 50 | 3.34 | 4 | UNC0642 | G9a and GLP HMTs |
| 60 | 6 | 3.3 | 2.87 | PCI 34051 | HDAC8 |
| 61 | 0.75 | 3.12 | 1.86 | CAY10591 | SIRT1 class III HDACs |
| 62 | 50 | 3.11 | 2.39 | Tubacin | HDAC6 |
| 63 | 12.5 | 2.92 | 3.46 | BSI-201 | PARP1 |
| 64 | 1 | 2.89 | 1.54 | Splitomicin | Sir2p HDAC activity |
| 65 | 50 | 2.85 | 3.44 | RVX-208 | BET bromodomains BD2 |
| 66 | 1.5 | 2.81 | 4.23 | Apicidin | HDACs |
| 67 | 17.5 | 2.75 | 2.05 | SIRT1/2 Inhibitor IV | SIRT 1 and SIRT2 |
| 68 | 50 | 2.72 | 1.58 | UNC0646 | G9a and GLP HMTs |
| 69 | 50 | 2.6 | 1.79 | RSC-133 | HDAC and DNA MTs |
| 70 | 50 | 2.57 | 1.46 | B32B3 | H2A T120 phosphorylation |
| 71 | 50 | 2.14 | 0.96 | 5-Methylcytidine | DNA methylation |
| 72 | 50 | 1.93 | 0.88 | 3-Deazaneplanocin A (hydrochloride) | H3 K27 methyltransferase EZH2 |
| 73 | 50 | 1.26 | 0.58 | WDR5-0103 | Histone MTs |
| 74 | 1.5 | 1.1 | 0.3 | C646 | p300 HAT |
| 75 | 50 | 0.92 | 0.9 | UNC1215 | L3MBTL3 methyl lysine reader |
| 76 | 50 | 0.86 | 0.55 | GSK126 | H3 K27 methyltransferase EZH2 |
| 77 | 50 | 0.82 | 0.71 | BRD73954 | HDAC6, 8 |

| | | | | | |
|---|---|---|---|---|---|
| 78 | 50 | 0.77 | 0.47 | Nicotinamide | HDAC |
| 79 | 1.5 | 0.61 | 0.64 | Garcinol | p300 PCAF HATs |
| 80 | 50 | 0.53 | 4.34 | MI-2 (hydrochloride) | HMTs |
| 81 | 4 | 0.53 | 0.64 | Scriptaid | HDACs |
| 82 | 50 | 0.45 | 0.62 | Nullscript | Negative control for Scriptaid |
| 83 | 1.5 | 0.43 | 0.34 | GSK4112 | HDAC3 activator |
| 84 | 50 | 0.35 | 0.34 | UNC0631 | G9a and GLP HMTs |
| 85 | 50 | 0.27 | 0.11 | 6-Thioguanine | DNA methylation inhibitor |
| 86 | 50 | 0.24 | 0.09 | CBHA | HDACs |
| 87 | 1.5 | 0.15 | 0.81 | CCG-100602 | Rho/MKL1/SRF transcription |
| 88 | 50 | 0.04 | 0.28 | Gemcitabine | DNA methylation |
| 89 | 50 | -0.02 | 0.97 | Piceatannol | Resveratrol analog, multiple actions |
| 90 | 50 | -0.2 | 0.11 | S-Adenosylhomocysteine | DNA MTs |
| 91 | 50 | -0.26 | 0.21 | ITF 2357 | Class I and II HDACs |
| 92 | 6 | -0.32 | 0.46 | Lomeguatrib | $O^6$-Methylguanine-DNA methyltransferase (MGMT) inhibitor |
| 93 | 50 | -0.33 | 0.93 | SGC0946 | DOT1 H3K79 methylation |
| 94 | 50 | -0.39 | 0.3 | Octyl-α-ketoglutarate | Promotes demethylase function |
| 95 | 50 | -0.52 | 0.6 | GSK-J2 (sodium salt) | Negative control for GSK-J1 |
| 96 | 50 | -0.61 | 0.31 | coumarin-SAHA | Class I and II HDACs |
| 97 | 50 | -0.69 | 0.62 | SB939 | HDACs |
| 98 | 50 | -1.06 | 1.79 | PFI-1 | BRD2 and 4 bromodomains |
| 99 | 5 | -1.11 | 0.42 | Salermide | SIRT1 and SIRT2 HDAC |
| 100 | 25 | -1.12 | 0.85 | Sodium 4-Phenylbutyrate | HDACs |
| 101 | 50 | -1.13 | 1.16 | CUDC-101 | Class I and II HDACs, HER1 and 2 |
| 102 | 0.5 | -1.22 | 0.54 | 2-hexyl-4-Pentynoic Acid | HDACs (derivative of VPA) |
| 103 | 50 | -1.23 | 0.41 | Cl-Amidine (trifluoroacetate salt) | Protein arginine deaminases |
| 104 | 50 | -1.23 | 1.86 | HPOB | HDAC6 |
| 105 | 50 | -1.67 | 1.15 | GSK-LSD1 (hydrochloride) | Lysine demethylase 1 |
| 106 | 50 | -1.78 | 2.27 | MS-436 | BRD4 bromodomain 1 |
| 107 | 50 | -1.79 | 1.82 | EPZ005687 | EZH2 blocks trimethylation H3K27 |
| 108 | 50 | -2.16 | 1.78 | CI-994 | Class I HDAC |
| 109 | 50 | -2.26 | 1.59 | AZ 505 | SMYD2 lysine N-methyltransferase |
| 110 | 50 | -2.4 | 1.85 | α-Hydroxyglutaric Acid (sodium salt) | HDMs and DNA hydroxylases |
| 111 | 50 | -2.72 | 1.76 | AGK7 | Negative control for AGK2 |
| 112 | 50 | -2.79 | 0.84 | 5-Azacytidine | DNA MTs |
| 113 | 10 | -2.84 | 2.3 | UNC0638 | G9a and GLP HMTs |
| 114 | 50 | -3.14 | 5.05 | CAY10683 | HDAC2 and 6 |
| 115 | 50 | -3.62 | 1.92 | UNC1999 | EZH2 blocks trimethylation H3K27 |
| 116 | 3 | -3.64 | 1.95 | HNHA | HDACs |
| 117 | 50 | -3.65 | 1.9 | 3-Deazaneplanocin A | EZH2 blocks trimethylation H3K27 |
| 118 | 50 | -4 | 3.07 | CAY10603 | HDAC6 |
| 119 | 50 | -4.15 | 2.24 | F-Amidine (trifluoroacetate salt) | Protein arginine deaminases PAD1 and 4 |
| 120 | 0.5 | -4.17 | 2.19 | JIB-04 | Jumonji HDMs |

| | | | | | |
|---|---|---|---|---|---|
| 121 | 50 | -4.73 | 2.8 | Tubastatin A (trifluoroacetate salt) | HDAC6 |
| 122 | 50 | -4.97 | 1.88 | GSK343 | EZH2 blocks trimethylation H3K27 |
| 123 | 3 | -5.46 | 2.03 | Phthalazinone pyrazole | Aurora kinases |
| 124 | 50 | -6.74 | 3.09 | Suberohydroxamic Acid | HDAC1 and 3 |
| 125 | 50 | -6.82 | 1.89 | Mirin | Mre11-Rad50-Nbs1 phosphorylation of H2AX |
| 126 | 50 | -7.7 | 3.92 | Delphinidin (chloride) | p300/CBP HATs |
| 127 | 25 | -7.79 | 2.82 | Panobinostat | class I, II, and IV histone deacetylases (HDACs) |
| 128 | 50 | -7.96 | 2.84 | Sinefungin | methyltransferases |
| 129 | 50 | -8.24 | 3.95 | (-)-Neplanocin A | methyltransferases |
| 130 | 12.5 | -8.44 | 2.77 | GSK-J4 (hydrochloride) | H3K27 histone demethylase JMJD3 |
| 131 | 50 | -8.85 | 2.45 | Pimelic Diphenylamide 106 | Class I HDACs |
| 132 | 50 | -9.42 | 5.18 | RGFP966 | HDAC3 |
| 133 | 50 | -9.82 | 2.25 | M 344 | HDAC1, 6 |
| 134 | 5 | -10.12 | 3.75 | Sirtinol | SIRT1, 2 |
| 135 | 50 | -10.19 | 4.06 | UNC0321 (trifluoroacetate salt) | G9a HMT |
| 136 | 50 | -10.63 | 4.03 | JGB1741 | SIRT1 |
| 137 | 20 | -11.38 | 4.51 | DMOG | HIF-α prolyl hydroxylase |
| 138 | 50 | -11.51 | 4.35 | MS-275 | HDAC1 |
| 139 | 50 | -12.81 | 2.67 | Pyroxamide | HDAC1 |
| 140 | 3 | -13.41 | 2.9 | GSK-J5 (hydrochloride) | Negative control for GSK-J4 |
| 141 | 50 | -14.16 | 4.38 | 4-iodo-SAHA | Class I and II HDACs |
| 142 | 40 | -14.98 | 5.08 | 2-PCPA (hydrochloride) | Lysine-specific demethylase 1 (LSD1) |
| 143 | 3 | -15.56 | 2.69 | Tenovin-1 | SIRT1 and 2 |
| 144 | 40 | -17.76 | 4.4 | SAHA | Class I, II and IV HDACs |
| 145 | 3 | -20.87 | 4.95 | SGC-CBP30 | CBP EP300 |
| 146 | 50 | -22.51 | 5.09 | 3-amino Benzamide | PARPs |
| 147 | 12.5 | -35.23 | 7.43 | EX-527 | SIRT1 |

**Supplementary Figure S21 – Hits recovered from epigenetic library screen**

a

*Hit #1*

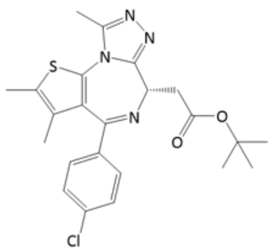

| (+)-JQ1 | |
|---|---|
| *Concentration:* | 2 µM |
| *VMR light rescue (QC 1.0):* | 75 ± 7.9 % |
| *VMR dark rescue (QC 0.96):* | 14 ± 2.5 % |
| *Spontaneous activity in light rescue (QC 0.28):* | 91 ± 15.7 % |
| *Target:* | BET bromodomain inhibitor |

*Hit #2*

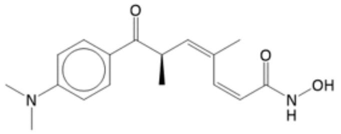

| Trichostatin-A | |
|---|---|
| *Concentration:* | 0.3 µM |
| *VMR light rescue (QC 1.0):* | 67 ± 5.1 % |
| *VMR dark rescue (QC 0.95):* | −6.2 ± 6.2 % |
| *Spontaneous activity in light rescue (QC 0.48):* | 61 ± 13.2 % |
| *Target:* | Reversible HDAC I, II, IV inhibitor |

*Hit #3*

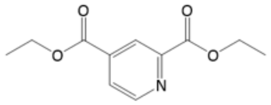

| 2, 4-Diethylpyridine dicarboxylate | |
|---|---|
| *Concentration:* | 6 µM |
| *VMR light rescue (QC 1.0):* | 55 ± 4.0 % |
| *VMR dark rescue (QC 0.95):* | 9.3 ± 4.4 % |
| *Spontaneous activity in light rescue (QC 0.81):* | 18 ± 8.1 % |
| *Target:* | Reversible HIF-PH inhibitor |

b

*Negative control*

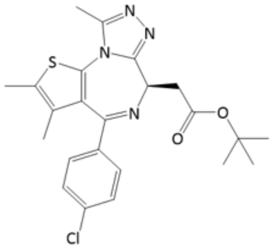

| (−)-JQ1 | |
|---|---|
| Concentration: | 5 µM |
| VMR light rescue (QC 1.0): | 6.3 ± 5.4 % |
| VMR dark rescue (QC 0.96): | −8.1 ± 4.4 % |
| Spontaneous activity in light rescue (QC 0.28): | −3.7 ± 2.5 % |
| Target: | Inactive stereoisomer of (+)-JQ1 |

c

*Other compound of potential interest #1*

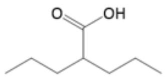

| Valproic acid | |
|---|---|
| *Concentration:* | 5 µM |
| *VMR light rescue (QC 1.0):* | 46 ± 6.5 % |
| *VMR dark rescue (QC 1.0):* | −0.6 ± 0.5 % |
| *Spontaneous activity in light rescue (QC 0.24):* | 68 ± 19.1 % |
| *Target:* | Reversible multi-HDAC inhibitor |

*Other compound of potential interest #2*

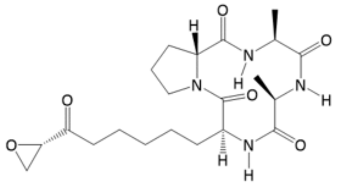

| HC Toxin | |
|---|---|
| *Concentration:* | 20 µM |
| *VMR light rescue (QC 1.0):* | 41 ± 3.6 % |
| *VMR dark rescue (QC 0.96):* | −7.8 ± 7.6 % |
| *Spontaneous activity in light rescue (QC 0.99):* | 88 ± 5.9 % |
| *Target:* | Reversible multi-HDAC inhibitor |

The chemical structures, maximum tolerated concentrations, phenotypic rescue, and molecular targets are summarized for compounds identified as active in this screen.

a: The three compounds that satisfied *a priori* criteria for a 'hit'
b: (−)JQ1 is a negative control stereoisomer of (+)JQ1; its lack of activity in this assay is strongly suggestive that (+)JQ1 exerts its effect through a specific receptor-ligand interaction
c: Other compounds that did not satisfy *a priori* criteria as 'hits', but appeared to show some activity in this assay and may therefore be of interest.

## Supplementary Table 16 – Data summary by target

| | **BET BDs** | **Multi HDAC** | **DNA MT** | **HAT** | **Other** | **HMT** | **Class 2 HDAC** | **HDM** | **Class 1 HDAC** | **Sirtuins** |
|---|---|---|---|---|---|---|---|---|---|---|
| Number of values | 10 | 24 | 12 | 9 | 25 | 23 | 6 | 12 | 10 | 11 |
| | | | | | | | | | | |
| Minimum | -1.778 | -17.76 | -2.792 | -7.701 | -22.51 | -10.19 | -4.726 | -14.98 | -12.81 | -35.23 |
| 25% Percentile | 1.873 | -1.127 | 0.0989 | 0.518 | -3.208 | -3.618 | -4.181 | -3.73 | -10.24 | -10.63 |
| Median | **10.95** | **0.6497** | **6.683** | **3.893** | **2.566** | **0.5313** | **0.9371** | **5.814** | **-7.798** | **2.755** |
| 75% Percentile | 22 | 24.96 | 15.59 | 14.14 | 9.195 | 3.339 | 11.17 | 19.6 | -0.7922 | 7.506 |
| Maximum | 74.89 | 66.92 | 30.75 | 25.03 | 55.17 | 19.96 | 12.58 | 25.92 | 19.78 | 19.11 |
| | | | | | | | | | | |
| Mean | **16.98** | **9.796** | **8.997** | **6.417** | **4.295** | **1.699** | **2.739** | **6.052** | **-4.137** | **-2.695** |
| Std. Deviation | 22.68 | 20.45 | 10.56 | 10.26 | 15.38 | 7.789 | 7.448 | 13.16 | 9.726 | 14.58 |
| Std. Error of Mean | 7.174 | 4.175 | 3.048 | 3.422 | 3.077 | 1.624 | 3.041 | 3.798 | 3.076 | 4.395 |
| Lower 95% CI of mean | 0.7528 | 1.16 | 2.289 | -1.473 | -2.056 | -1.669 | -5.077 | -2.307 | -11.09 | -12.49 |
| Upper 95% CI of mean | 33.21 | 18.43 | 15.71 | 14.31 | 10.65 | 5.067 | 10.56 | 14.41 | 2.821 | 7.099 |
| *One sample t test* | | | | | | | | | | |
| Theoretical mean | 0 | 0 | 0 | 0 | 0 | 0 | 0 | 0 | 0 | 0 |
| Actual mean | 16.98 | 9.796 | 8.997 | 6.417 | 4.295 | 1.699 | 2.739 | 6.052 | -4.137 | -2.695 |
| Discrepancy | 16.98 | 9.796 | 8.997 | 6.417 | 4.295 | 1.699 | 2.739 | 6.052 | -4.137 | -2.695 |
| 95% CI of discrepancy | 0.7528 to 33.21 | 1.160 to 18.43 | 2.289 to 15.71 | -1.473 to 14.31 | -2.056 to 10.65 | -1.669 to 5.067 | -5.077 to 10.56 | -2.307 to 14.41 | -11.09 to 2.821 | -12.49 to 7.099 |
| t, df | t=2.367, df=9 | t=2.346, df=23 | t=2.952, df=11 | t=1.875, df=8 | t=1.396, df=24 | t=1.046, df=22 | t=0.9008, df=5 | t=1.593, df=11 | t=1.345, df=9 | t=0.6131, df=10 |
| P value (two tailed) | **0.0421** | **0.0279** | **0.0132** | **0.0976** | **0.1755** | **0.3069** | **0.409** | **0.1394** | **0.2115** | **0.5535** |
| Significant? | Yes | Yes | Yes | No | No | No | No | No | No | No |

**Supplementary Figure S22 – Ranked phenotypic rescue activity by target**

a

| Target class | Number of compounds | Mean % rescue | Median rank |
|---|---|---|---|
| BET | 10 | 16.98 | 42 |
| Multi HDAC | 24 | 9.796 | 78.5 |
| DNA MT | 12 | 8.997 | 46.5 |
| HAT | 9 | 6.417 | 56 |
| HDM | 12 | 6.052 | 50 |
| Other | 25 | 4.295 | 65.5 |
| Class 2 HDAC | 6 | 2.739 | 81 |
| HMT | 23 | 1.699 | 79 |
| Sirtuins | 11 | -2.695 | 66 |
| Class 1 HDAC | 10 | -4.137 | 123.5 |

b

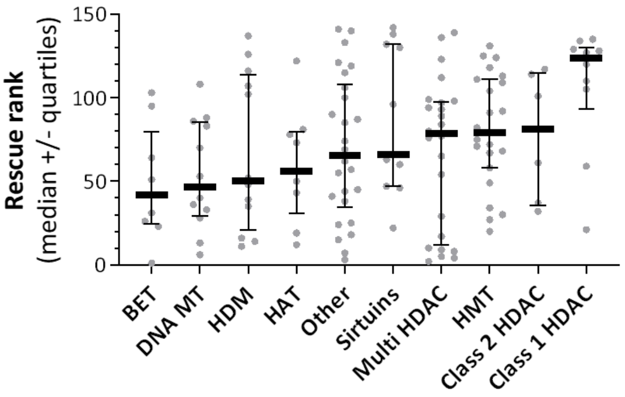


Rescue data grouped by pharmacological target, similar to Figure 7e and Supplementary Table 16, but analyzed by rank rather than % rescue.

a: Table showing mean % rescue and median rank by pharmacological target
b: Graph showing rescue rank by target. Data points show individual chemicals, bars show median and quartiles.

The data show similar trends to Figure 7e, with BET bromodomain inhibitors showing the lowest median rescue rank.

**Supplementary Table 17 – Properties of compounds categorized as 'other' in Figure 7e**

| Rank | Mean % rescue | Compound | Target |
|---|---|---|---|
| 3 | 55.16842 | 2,4-DPD | HIF-α prolyl hydroxylase |
| 15 | 23.59271 | Lestaurtinib | JAK2, FLT3, TrkA |
| 18 | 20.29308 | Tenovin-6 | p53 activator |
| 24 | 17.80692 | Isoliquiritigenin | Guanyl cyclase activator |
| 25 | 17.38939 | Ellagic Acid | coactivator-associated arginine methyltransferase 1 |
| 38 | 9.604486 | Suramin (sodium salt) | Topoisomerase II |
| 41 | 8.784877 | Etoposide | Topoisomerase II |
| 44 | 8.049176 | Rucaparib (phosphate) | PARP1 |
| 45 | 7.831244 | (+)-Abscisic Acid | Chromatin structure gene expression |
| 55 | 3.898963 | AMI-1 (sodium salt) | Arginine methyltransferases |
| 57 | 3.425327 | I-CBP112 (hydrochloride) | CBP EP300 |
| 62 | 2.921351 | BSI-201 | PARP1 |
| 69 | 2.565975 | B32B3 | H2A T120 phosphorylation |
| 74 | 0.915359 | UNC1215 | L3MBTL3 methyl lysine reader |
| 85 | 0.148336 | CCG-100602 | Rho/MKL1/SRF transcription |
| 87 | -0.02275 | Piceatannol | Resveratrol analog, multiple actions |
| 90 | -0.31731 | Lomeguatrib | $O^6$-Methylguanine-DNA methyltransferase (MGMT) |
| 100 | -1.22962 | Cl-Amidine (trifluoroacetate salt) | Protein arginine deaminases |
| 106 | -2.26145 | AZ 505 | SMYD2 lysine N-methyltransferase |
| 115 | -4.15488 | F-Amidine (trifluoroacetate salt) | Protein arginine deaminases PAD1 and 4 |
| 119 | -5.46097 | Phthalazinone pyrazole | Aurora kinases |
| 121 | -6.81759 | Mirin | Mre11-Rad50-Nbs1 phosphorylation of H2AX |
| 133 | -11.3792 | DMOG | HIF-α prolyl hydroxylase |
| 140 | -20.8708 | SGC-CBP30 | CBP EP300 |
| 141 | -22.5056 | 3-amino Benzamide | PARPs |

**Supplementary Figure S23 – Verification of hits by analysis of repurchased compound**

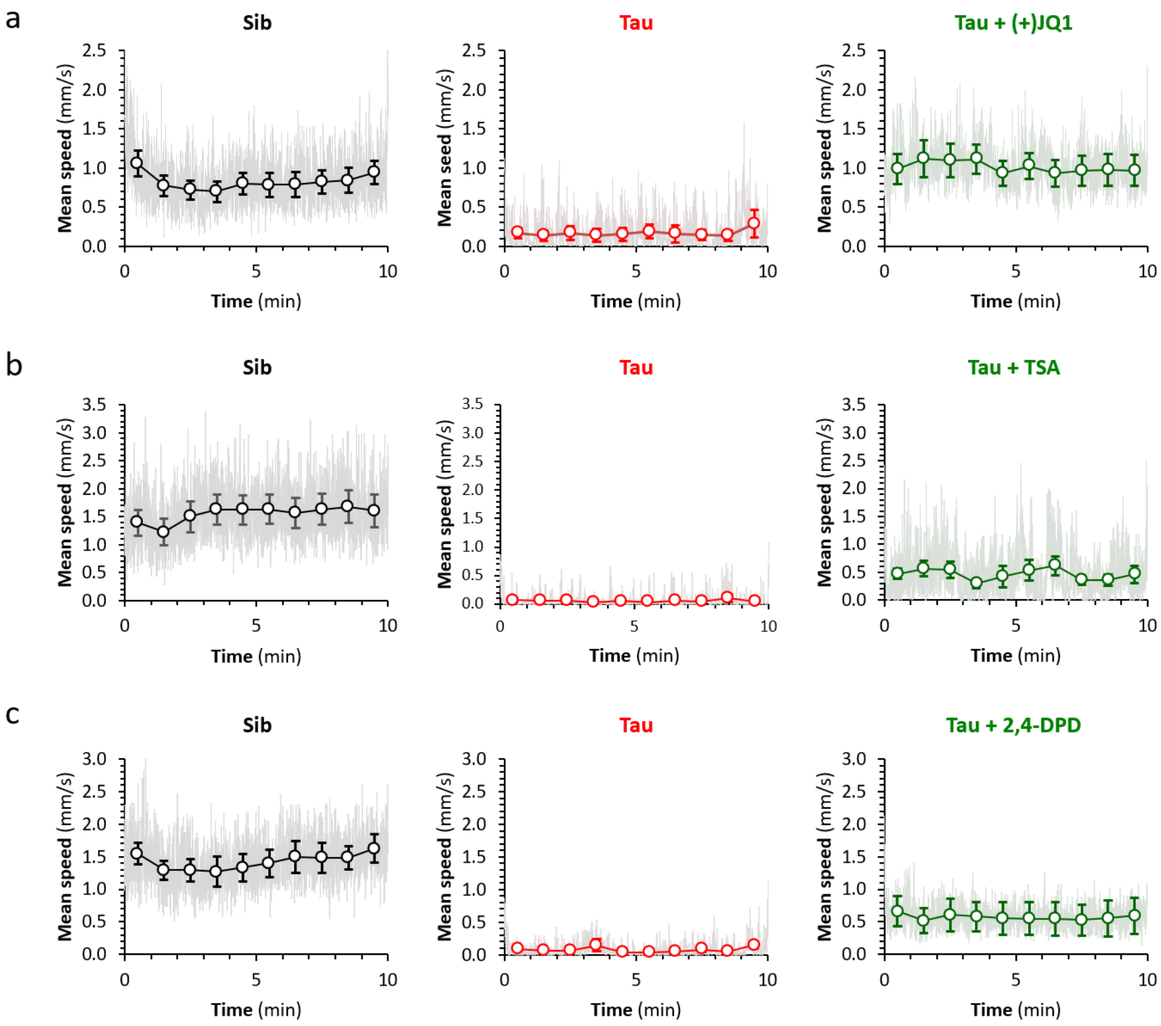


Library molecules that rescued motor phenotypes in the primary screen were repurchased and tested at the same purported concentrations in motor rescue assays to verify the molecular identities of the hits. Sibling controls (left column; black) and untreated Tau zebrafish (center column; red), were compared with Tau zebrafish exposed to compound following dechorionation at 2dpf until 5dpf (right column, green). 96-well plate motor assays were run at 5dpf as shown in Figures 5 and 8. The graphs show mean speed during the light phase of the VMR. The light traces show mean frame-to-frame displacement scaled to speed; colored markers and bars show 1-minute binned group mean ± SE, similar to Figure 8a.

a: (+)JQ1 (1.5µM) repurchased from Sigma (batch # 0000083981; these are the same data as Figure 8a reproduced here for clarity)

b: Trichostatin-A (0.2 µM) repurchased from Cayman Chemical (batch # 0437853431)

c: 2,4-Diethylpyridine dicarboxylate (6µM) repurchased from Cayman Chemical (batch # 181397-27)

All three compounds partially rescued VMR light phase hypokinesia in Tau zebrafish, verifying the identities of the hits found in the primary screen using the same assay.

**Supplementary Figure S24 – Concentration-dependent rescue of motor function in Tau zebrafish by (+)JQ1**

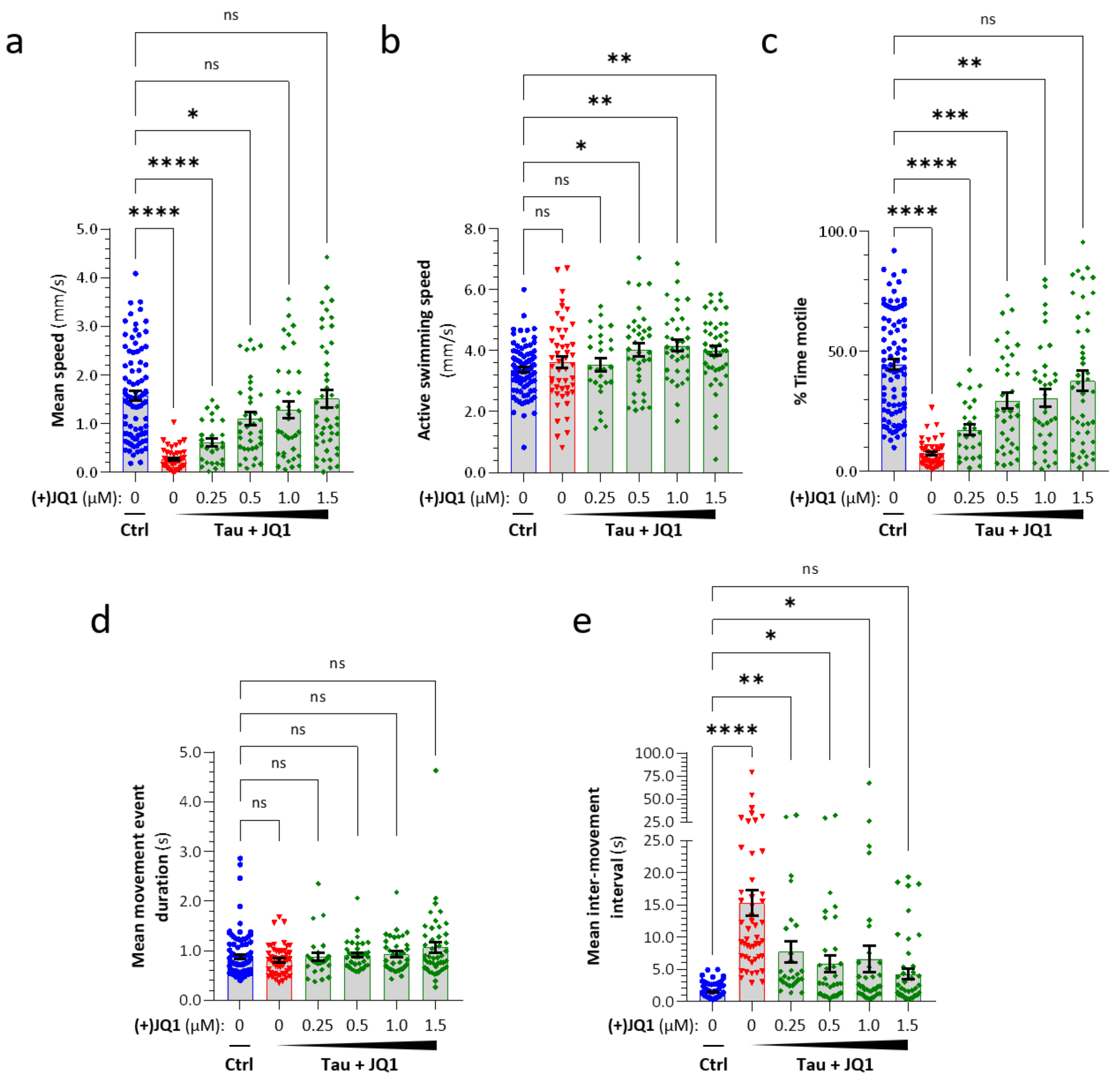


Similar analyses to Figure 5c – 5g, showing how abnormalities of motor activity in Tau zebrafish at 5dpf were altered by exposure to (+)JQ1 between 2 – 5 dpf, at incremental concentrations from 0.25 to 1.5µM. In each graph, data points show individual zebrafish, bars show mean ± SE. *****p*<0.0001, ****p*<0.001, ***p*<0.01, **p*<0.05, Ctrl *vs*. experimental group, 1-way ANOVA with Dunnett's multiple comparison test.

a: Mean speed (scalar distance traveled by zebrafish centroid/time of assay)
b: Active swimming speed (mean scalar speed of zebrafish centroid during movement events)
c: Time active % (proportion of video frame transitions at which displacement occurred)
d: Mean movement event duration (mean duration of movement episodes)
e: Mean inter-movement interval (mean duration of stationary periods between movements)

Motor abnormalities in Tau zebrafish improved with increasing concentrations of (+)JQ1.

**Supplementary Table 18 - Exact *p*-values for Figure 8b, 8d – 8i**

| Panel | Statistical test | Comparison group 1 | Comparison group 2 | Adjusted *p* value |
|---|---|---|---|---|
| | | | | |
| 8b | One-way ANOVA with Dunnett multiple comparisons test | Ctrl | Tau | 0.000000000000083 |
| | | | Tau + 0.25µM (+)JQ1 | 0.000003472349992 |
| | | | Tau + 0.5µM (+)JQ1 | 0.029010139082927 |
| | | | Tau + 1.0 µM (+)JQ1 | 0.359887638918267 |
| | | | Tau + 1.5 µM (+)JQ1 | 0.997796583767877 |
| | | | | |
| 8d | One-way ANOVA with Šidák multiple comparisons test | Ctrl | Ctrl + (+)JQ1 | 0.999942977282417 |
| | | Ctrl | Tau | 0.000000006937303 |
| | | Tau | Tau + (+)JQ1 | 0.000489002947961 |
| | | | | |
| 8e | 2-tailed t-test with Welch correction | Tau | Tau + (+)JQ1 | 0.008717663859809 |
| | | | | |
| 8f | One-way ANOVA with Dunnett multiple comparisons test | Ctrl | Tau | 0.000000000005670 |
| | | Tau | Tau + (+)JQ1 | 0.992031122504968 |
| | | | | |
| 8g | One-way ANOVA with Šidák multiple comparisons test | Ctrl | Ctrl + (+)JQ1 | 0.998757810893752 |
| | | Ctrl | Ctrl + (−)JQ1 | 0.999993794290067 |
| | | Ctrl | Tau | <0.000000000000001 |
| | | Tau | Tau + (+)JQ1 | 0.000000013851543 |
| | | Tau | Tau + (−)JQ1 | 0.483623885971886 |
| | | | | |
| 8h | One-way ANOVA with Šidák multiple comparisons test | Ctrl | Ctrl + (+)JQ1 | 0.010057465092475 |
| | | Ctrl | Ctrl + (−)JQ1 | 0.987460255302511 |
| | | Ctrl | Tau | 0.000000000000095 |
| | | Tau | Tau + (+)JQ1 | <0.000000000000001 |
| | | Tau | Tau + (−)JQ1 | 0.999907116208083 |
| | | | | |
| 8i | One-way ANOVA with Tukey multiple comparisons test | Ctrl | Tau | 0.000018040608642 |
| | | Tau | Tau + (+)JQ1 | 0.419406610554096 |
| | | Ctrl | Tau + (+)JQ1 | 0.000352844802280 |

**Supplementary Figure S25 – Specificity controls for (+)JQ1 rescue of motor function in Tau zebrafish**

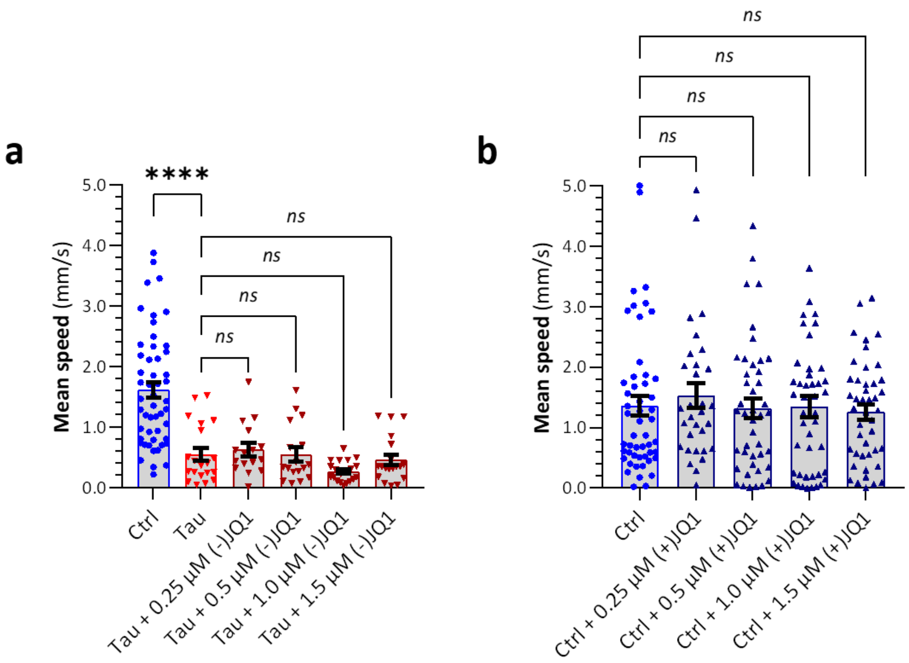


Mean swimming speed of Ctrl and Tau zebrafish during the light phase of the VMR is shown at 5 dpf following exposure to small molecules as indicated from 2 – 5 dpf.

a: Tau zebrafish exposed to incremental concentrations of the negative control stereoisomer (−)JQ1 that does not bind to protein bromodomains. Untreated Tau and Ctrl zebrafish are shown for comparison.

b: Ctrl zebrafish exposed to incremental concentrations of (+)JQ1. Untreated Ctrl zebrafish are shown for comparison.

In each graph, data points show individual zebrafish, bars show mean ± SE. ****$p$<0.0001, 1-way ANOVA with Dunnett's multiple comparisons test to compare each group to untreated (a) Tau or (b) Ctrl zebrafish.

In contrast to the robust (+)JQ1 dose-dependent rescue of VMR light phase hypokinesia in Tau zebrafish (Figures 8a – 8b, Supplementary Figure S24), the stereoisomer (−)JQ1 is inactive in this assay, strongly suggesting that the activity of (+)JQ1 is dependent on a specific interaction with a protein bromodomain. The lack of increased motor activity in Ctrl zebrafish exposed to (+)JQ1 further indicates that this compound does not influence zebrafish motility non-specifically, but instead acts by rescuing the phenotype caused by 0N/4R-Tau over-expression.

**Supplementary Figure S26 – (+)JQ1 does not silence transgene expression in Tau zebrafish**

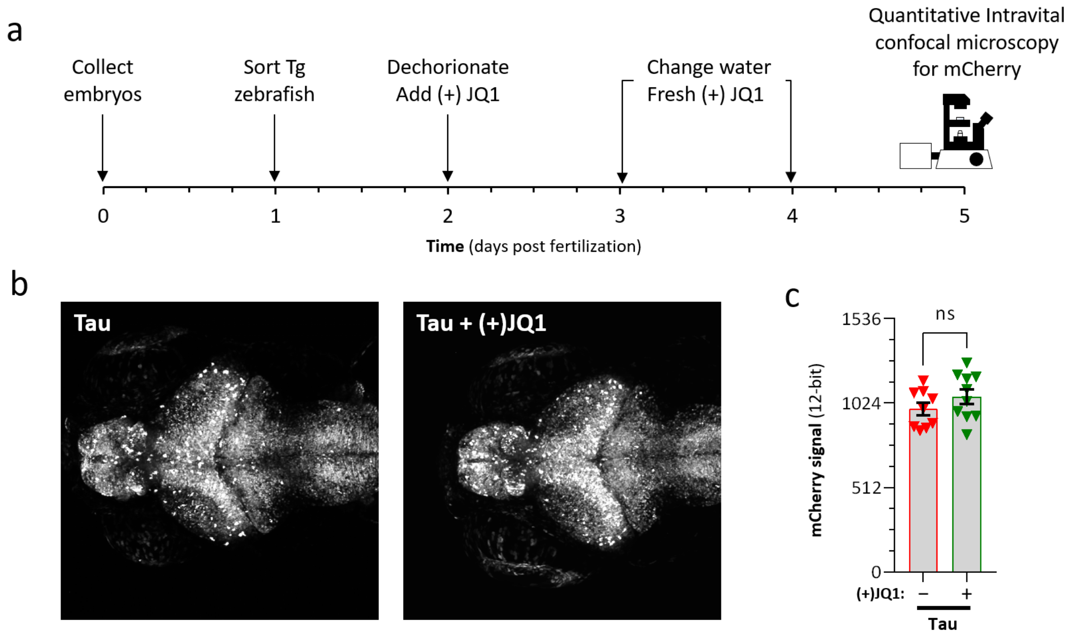


A critical validation for the screen shown in Figure 7 is to ensure that small molecules identified as rescuing phenotypes did not do so by silencing transgene expression, as compounds with this activity would likely be uninformative about the biology of tauopathy, instead reflecting properties of the transgene expression system. Co-expression of nls-mCherry with Tau in the model provides a marker of transgene expression and a rapid way to evaluate screen 'hits' for activity in interfering with transgene expression.

a: Schematic showing the design of the validation assay. Zebrafish were exposed to (+)JQ1 in an identical way to the screen, but instead of a movement assay, the endpoint was intravital confocal imaging.

b: Representative confocal Z-plane maximum intensity projections showing dorsal views of mCherry expression in live Tau zebrafish exposed to (+)JQ1 or no inhibitor as indicated.

c: Quantification of fluorescence signal in 9 zebrafish per group using NIS-Elements. Data points show individual zebrafish, bars show mean ± SE. Data analyzed by 2-way unpaired *t*-test.

There was no evidence of transgene expression being lost after exposure to (+)JQ1. Note that the steady-state level of 0N/4R-Tau could be influenced by other factors separate from transgene expression (such as Tau degradation, clearance, etc.). This is dealt with in Figure 8f.

**Supplementary Table 19 – Statistical analysis of Tau zebrafish survival in presence or absence of (+)JQ1**

| Group | Parameter | Replicate #1 | Replicate #2 | Replicate #3 |
|---|---|---|---|---|
| Tau vs. Tau + JQ1 | Mantel-Cox $\chi^2$ (df) | 16.4063968503581 (1) | 19.2694441904945 (1) | 20.1818785115746 (1) |
| | Mantel-Cox *p* | 0.000051112445882 | 0.000011350844466 | 0.000034474380916 |
| | Mantel-Haenszel hazard ratio (95% CI) | 6.44997684996026 (3.85 – 28.37) | 9.53997776573499 (3.60 – 27.00) | 3.82608678934647 (2.13 – 6.87) |
| Tau | Median survival | 11 | 11.5 | 11.5 |
| Tau + JQ1 | | 14 | 14 | 13 |

**Supplementary Table 20 – Statistical analysis of Ctrl zebrafish survival in presence or absence of (+)JQ1**

| Group | Parameter | Replicate #1 | Replicate #2 | Replicate #3 |
|---|---|---|---|---|
| Ctrl vs. Ctrl + JQ1 | Mantel-Cox $\chi^2$ (df) | 0.226102965462962 (1) | 0.0362666071172773 (1) | 0.0882470940061729 (1) |
| | Mantel-Cox *p* | 0.634428598517294 | 0.848965973660078 | 0.766417553251096 |
| | Mantel-Haenszel hazard ratio (95% CI) | 1.41471464022798 (0.34 – 6.0) | 0.883623837091171 (0.26 – 3.08) | 0.869975645802120 (0.35 – 2.18) |

**Supplementary Figure S27 – (+)JQ1 does not rescue OKR deficits in Tau zebrafish**

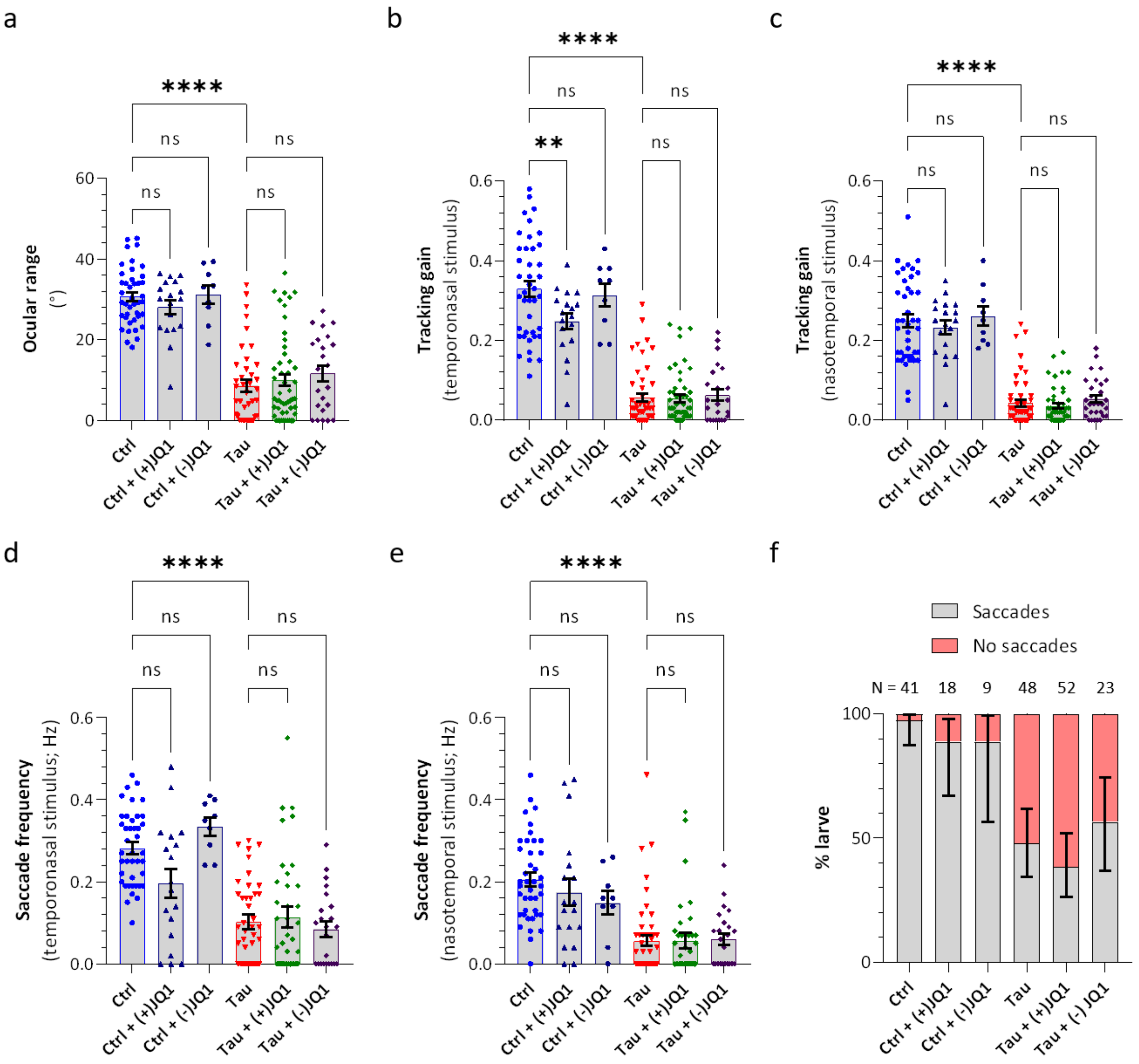


Similar analyses to Figure 6 and Supplementary Figure S15, showing quantification of optokinetic reflexes in Ctrl and Tau zebrafish at 5dpf after no treatment, exposure to (+)JQ1, or exposure to (−)JQ1 from 2 – 5 dpf. In graphs a – e, data points show individual zebrafish, bars show mean ± SE. ****$p$<0.0001, **$p$<0.01, 1-way ANOVA with Šidák multiple comparison test. In panel (f), bars show proportion ± 95% CI of zebrafish with and without saccadic movements during exposure to OKR stimuli.

In contrast to motor function and survival, there was no evidence that (+)JQ1 rescued OKR deficits in the Tau model after treatment at these time points.

**Supplementary Figure S28 – Volumetric imaging of synapse abundance in zebrafish larval brain**

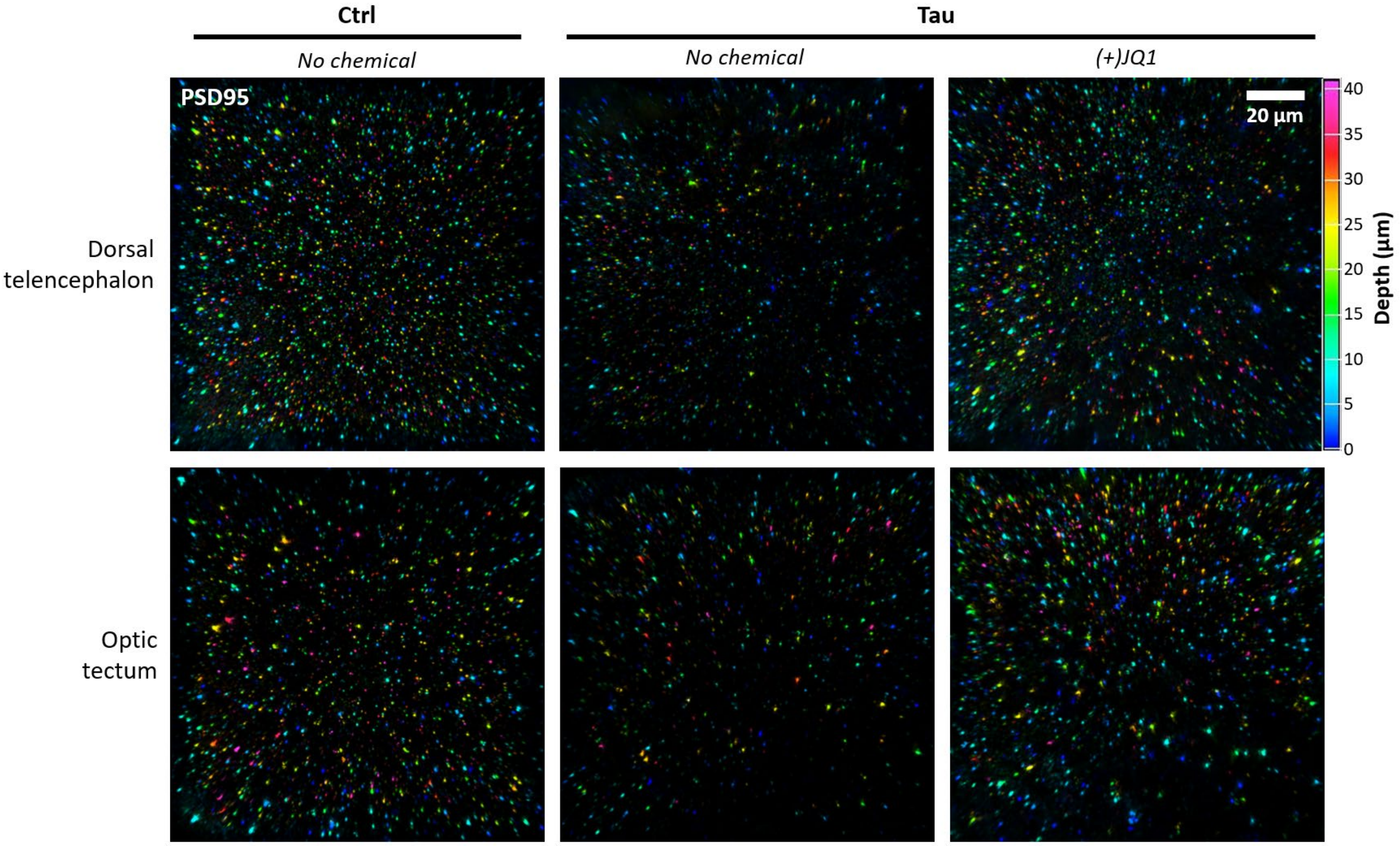


PSD95 whole mount immunolabeling and volumetric image acquisition in the telencephalon (upper row) and optic tectum (lower row) were carried out in Ctrl (left), Tau (center) and [Tau + (+)JQ1] (right) zebrafish. The figure shows the same samples as Figure 9a, but the images here are alpha-blended and pseudocolored to show depth within the image stack, illustrating the large volume of PSD95 expression data that was captured by this method.

**Supplementary Table 21 – Exact *p*-values for Figure 9b, 9c, 9e, 9g**

| Panel | Statistical test | Comparison group 1 | Comparison group 2 | Adjusted *p* value |
|---|---|---|---|---|
| | | | | |
| 9b | One-way ANOVA with Šidák multiple comparisons test | Ctrl | Ctrl + (+)JQ1 | 0.919409980094170 |
| | | Ctrl | Tau | 0.000003356940192 |
| | | Tau | Tau + (+)JQ1 | 0.000993889661747 |
| | | Ctrl | Tau + (+)JQ1 | 0.166096447592438 |
| | | | | |
| 9c | One-way ANOVA with Šidák multiple comparisons test | Ctrl | Ctrl + (+)JQ1 | 0.893099808802923 |
| | | Ctrl | Tau | 0.000000000173235 |
| | | Tau | Tau + (+)JQ1 | 0.025239927938240 |
| | | Ctrl | Tau + (+)JQ1 | 0.000024080791842 |
| | | | | |
| 9e | One-way ANOVA with Tukey multiple comparisons test | Ctrl | Tau | 0.000000000007919 |
| | | Tau | Tau + (+)JQ1 | 0.000000002081638 |
| | | Ctrl | Tau + (+)JQ1 | 0.966489749127617 |
| | | | | |
| 9g | One-way ANOVA with Šidák multiple comparisons test | GFP | GFP + (+)JQ1 | 0.998900495477148 |
| | | GFP | Tau | 0.000000022461457 |
| | | Tau | Tau + (+)JQ1 | 0.000000161286393 |
| | | GFP | Tau + (+)JQ1 | 0.974162547746396 |

**Supplementary Figure S29 – Quantification of PSD95-immunoreactive synaptic material inside zebrafish microglia**

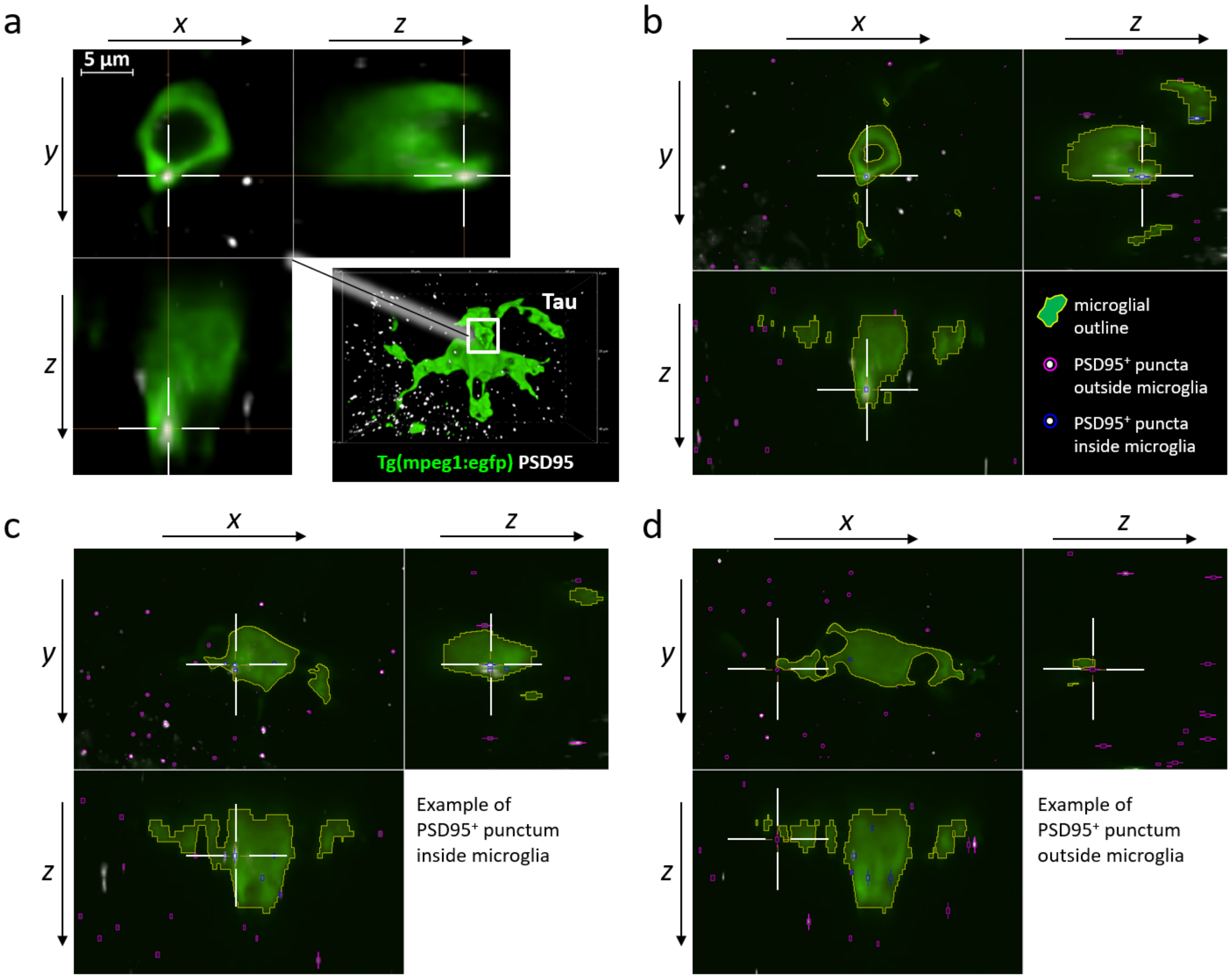


Confocal image stacks were acquired through whole mount zebrafish brains after immunolabeling for GFP, expressed from the Tg(*mpeg1:egfp*) transgene (labels the cytoplasm of microglia; green), and PSD95 (localized to post-synaptic densities; white).

a: The inset shows an alpha-blended 3D representation of a microglia from a Tau zebrafish brain. The three images show a single confocal plane through part of the microglial cell (x-y, top left). A PSD95 punctum that appears to be inside the microglial cytoplasm is indicated by cross hairs. The other two images show x-z and y-z reconstructions through the planes of the cross hairs, indicating that the punctum is unequivocally localized within the microglial cytoplasm in all three views.

b: A custom algorithm (written using NIS-Elements GA3) was used to identify, in 3 dimensions, the boundaries of each microglia (yellow) and the locations of all PSD95$^+$ puncta, then to assign each punctum to a position within (blue) or outside (magenta) microglia. The image shows the same confocal plane from panel (a), with multiple PSD95$^+$ puncta correctly assigned to locations within or outside microglia.

c, d: Further examples are shown of different confocal planes, in which PSD95$^+$ puncta are correctly assigned to positions (c) within the microglia, or (d) adjacent to but outside the microglia.

Using this algorithm, we were able to analyze the locations of a large number of PSD95 puncta with respect to a statistically robust sample of microglia (Figure 9d – 9e; Supplementary Figure S30).

**Supplementary Figure S30 – (+)JQ1 mitigates microglial accumulation of PSD95-immunoreactive synaptic material in Tau zebrafish**

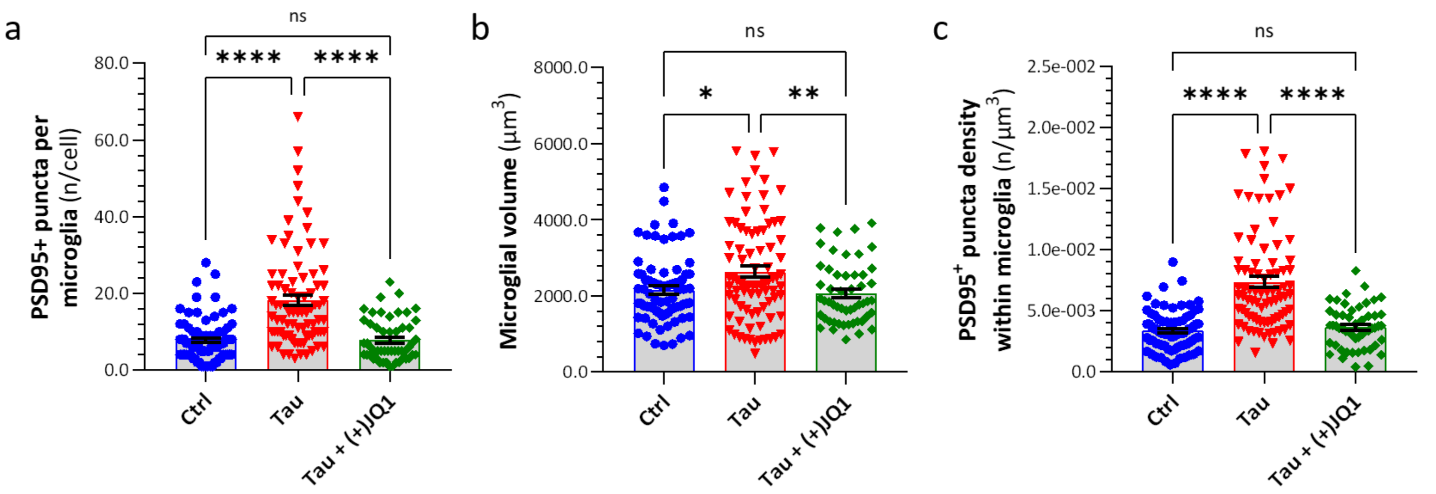


The method shown in Supplementary Figure S29 was used to quantify PSD95-immunoreactive puncta inside microglia of whole mount brain samples from 4dpf Ctrl (blue), Tau (red) or JQ1-treated Tau (green) zebrafish. Each line was first crossed with Tg(*mpeg1:egfp*) zebrafish to label microglial cytoplasm, and then fixed brains were immunolabeled for GFP and PSD95. In all three graphs, data points show individual microglia, bars show mean ± SE for the group. Multiple images were analyzed from 6 – 10 brains per group.

a: Number of $PSD95^+$ puncta per microglial cell

b: Volume of each microglial cell

c: Number of $PSD95^+$ puncta per cell normalized to the volume of the same cell to show density rather than abundance

In each graph ****$p<0.0001$, **$p<0.01$, *$p<0.05$, 1-way ANOVA with Tukey's multiple comparisons test.

Individual microglia in Tau zebrafish contained > 2-fold more PSD95-immunoreactive puncta than microglia from Ctrl zebrafish. Microglia from Tau zebrafish also showed slightly larger volume than Ctrl. The increase in microglial size in Tau zebrafish did not account for the increased abundance of $PSD95^+$ puncta, as the density of $PSD95^+$ puncta was also substantially increased in Tau zebrafish microglia compared with controls. These changes in $PSD95^+$ puncta/microglia, $PSD95^+$ puncta/volume, and microglial volume in Tau zebrafish were not seen after exposure to (+)JQ1.

Together these data support a model in which: (i) microglial phagocytosis of synaptic material contributes to the reduced density of $PSD95^+$ synaptic puncta seen in brains from Tau zebrafish; and (ii) (+)JQ1 decreases microglial phagocytosis of synaptic material.

**Supplementary Figure S31 – Rat primary co-culture system to test the activity of (+)JQ1 in mammalian microglia**

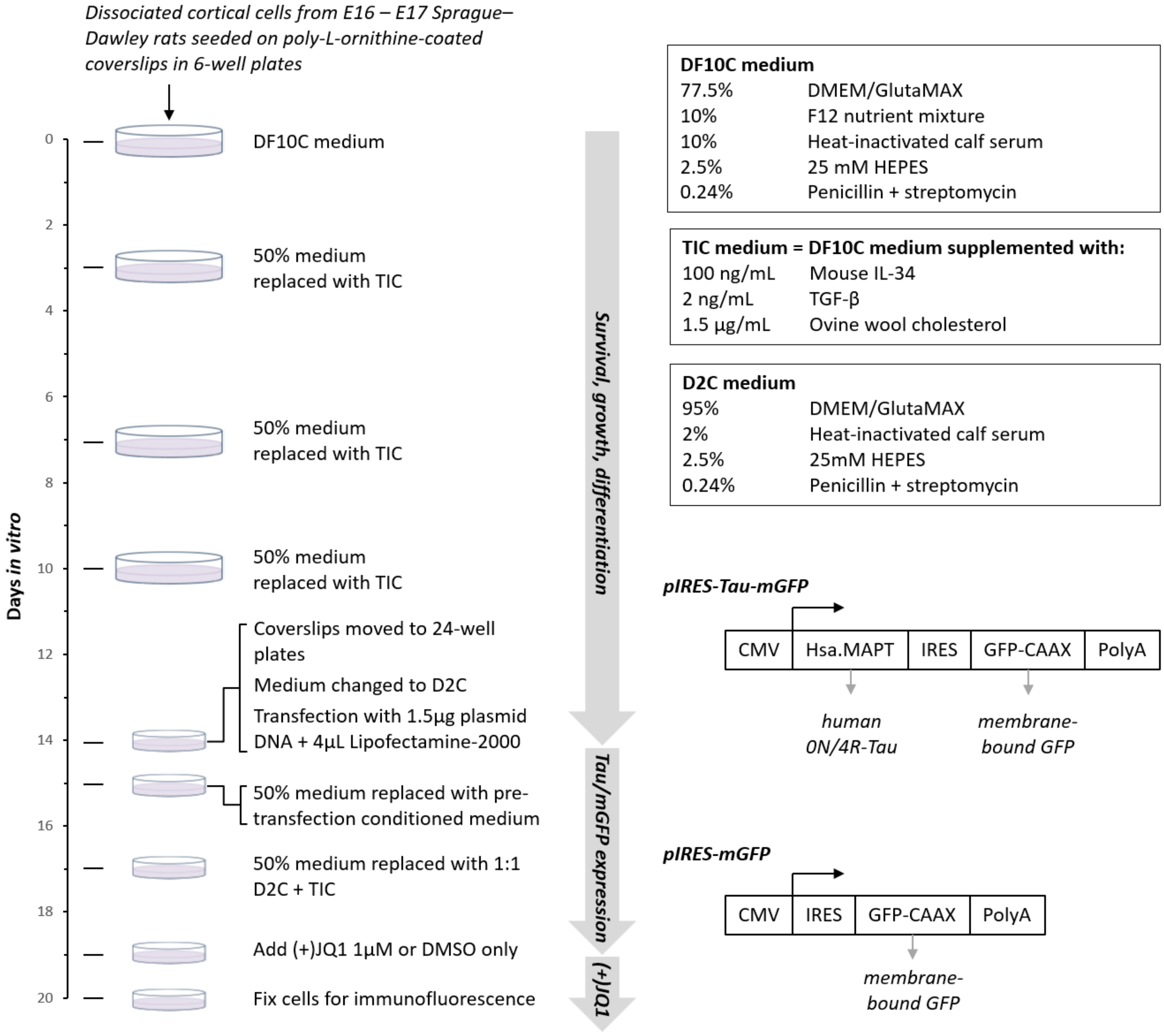


The left side of the schematic shows the procedure and time course for generating rat cortex primary cultures that contain neurons, astrocytes, and abundant microglia (this is modified from previous work[5] to enhance the survival and proliferation of microglia[6]). The key reagents used in this experiment are shown on the right side of the schematic. At 14 DIV, the established co-cultures were transfected so that some neurons expressed either: (i) human 4R/0N-Tau and membrane bound GFP (mGFP) as separate proteins, or (ii) mGFP alone, using the plasmid constructs shown lower right. The neurons expressed the transgene for 5 days, and then (+)JQ1, or DMSO alone at the same final concentration, was added to the culture medium. 24 hours later, the cells were fixed for immunofluorescence and microscopic analysis of synaptic material within microglia.

**Supplementary Figure S32 – Quantification of PSD95-immunoreactive puncta inside rat microglia**

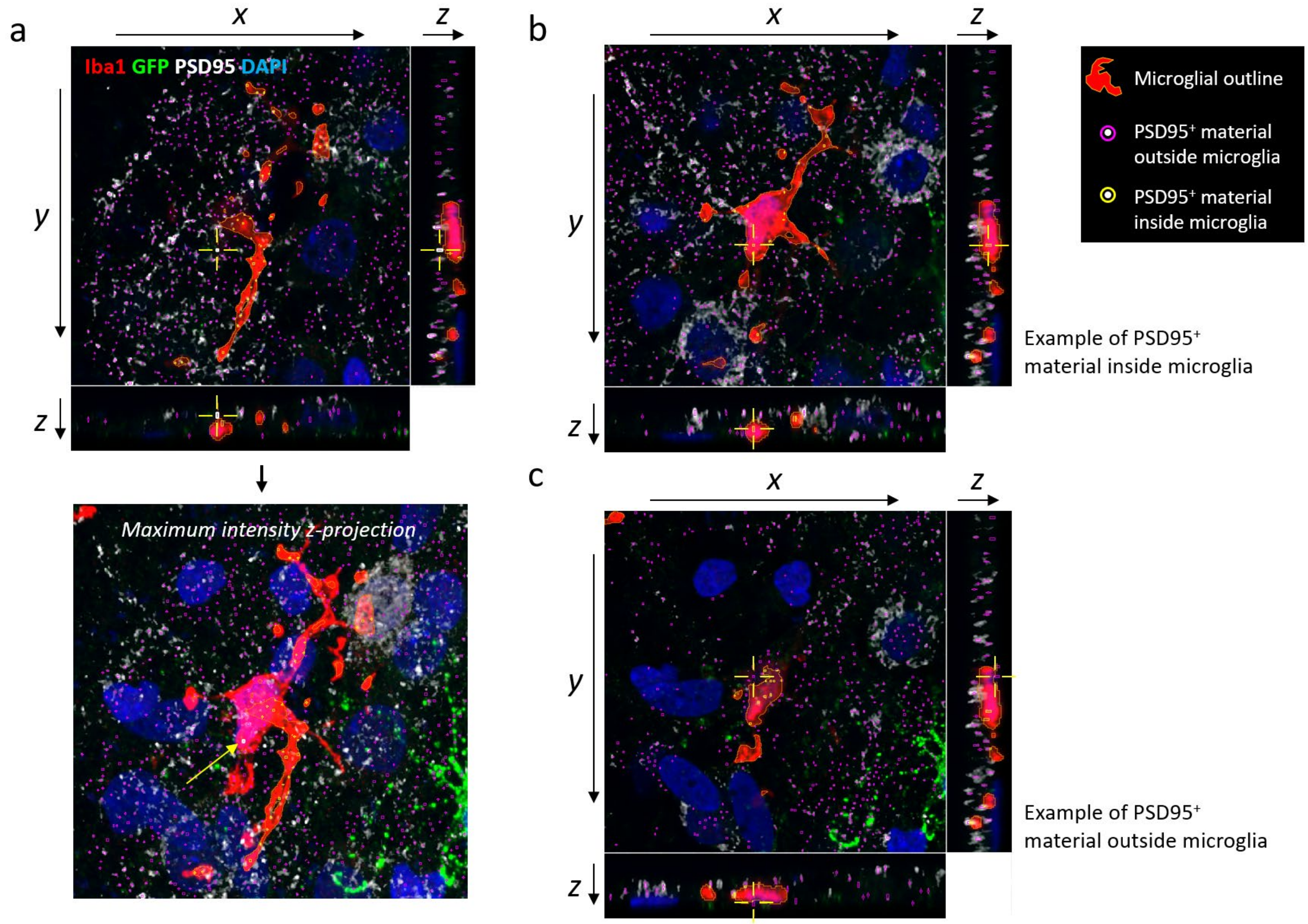


Confocal image stacks were acquired from rat primary cultures after immunolabeling for Iba1 (microglia; red), GFP (labels the plasma membrane of transfected neurons; green), PSD95 (post-synaptic densities; white) and DAPI (nuclei; blue). Similar to Supplementary Figure S29, a custom algorithm (written using NIS-Elements GA3) was used to identify, in 3 dimensions, the boundaries of each microglia (orange) and the locations of all PSD95$^+$ puncta, then to assign each PSD95$^+$ punctum to a position within (yellow) or outside (magenta) microglia.

a: Although contact cultures are flat compared with whole mount brain samples, the 3D analysis approach remains critical. The upper panel shows a single confocal plane through a microglial cell (x-y, top left), with x-z and y-z reconstructions through the planes of the cross hairs. A PSD95$^+$ punctum is unequivocally localized outside the microglial cytoplasm in the x-y plane, and it is seen lying above the microglia in the z-reconstructions. As a result, analysis of a z-plane maximum intensity projection image (lower panel) would incorrectly assign the punctum as inside the microglia (yellow arrow). Using stringent analysis parameters to maximize specificity in all three image planes, the algorithm performed well in correctly identifying PSD95$^+$ puncta inside microglia in this model.

b, c: Further examples are shown of different confocal planes, in which PSD95$^+$ puncta are correctly assigned to positions (b) within the microglia, or (c) closely adjacent to, but outside, the microglia.

Using this algorithm, we were able to analyze the locations of a large number of PSD95 puncta with respect to a statistically robust sample of microglia (Figures 9f – 9g; Supplementary Figure S33).

**Supplementary Figure S33 – (+)JQ1 mitigates accumulation of PSD95-immunoreactive puncta within rat microglia co-cultured with neurons transfected to express human 0N/4R-Tau**

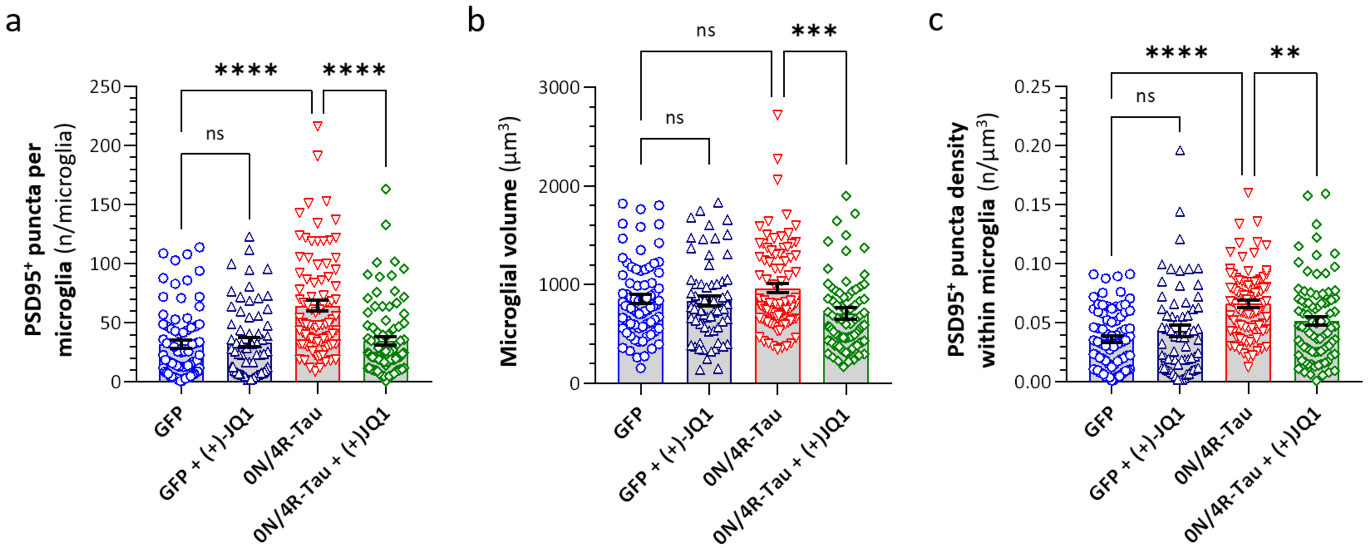


The methods shown in Supplementary Figures S38 and S39 were employed to quantify PSD95-immunoreactive puncta within microglia of primary rat cortex cultures, in which neurons were transfected to express GFP and 0N/4R-Tau, or GFP only, then exposed to D2C + TIC medium containing 1µM (+)JQ1, or medium containing the equivalent amount of DMSO only. Fixed cultures were immunolabeled for GFP (transfected neurons), Iba1 (microglia), and PSD95 (excitatory post-synaptic densities) and counter-labeled with DAPI (nuclei). In all three graphs, data points show individual microglia, bars show group mean ± SE.

a: Number of $PSD95^+$ puncta per microglial cell

b: Volume of each microglial cell

c: Number of $PSD95^+$ puncta per cell divided by the volume of the same cell to show density rather than abundance

In each graph *****p*<0.0001, ****p*<0.001, ***p*<0.01, 1-way ANOVA with Šidák multiple comparisons test.

Individual microglia from cultures in which neurons expressed human 0N/4R-Tau and GFP contained ≈ 2-fold more PSD95-immunoreactive puncta compared with microglia from control cultures, in which neurons were transfected to express GFP only. This increase was not present after treatment with (+)JQ1. Microglia from Tau-transfected cultures showed similar volumes to GFP-transfected cultures. A modest decrease in microglial volume in Tau-transfected cultures treated with (+)JQ1 did not account for the decreased abundance of microglial $PSD95^+$ puncta, as the density of $PSD95^+$ puncta was also substantially reduced after (+)JQ1 treatment.

Together these data provide an important validation that microglial phagocytosis of synaptic material in the presence of Tau over-expression, and its prevention by (+)JQ1, is phylogenetically conserved in a rat model. This suggests that the observations made in the zebrafish model may be translationally informative.

**Supplementary Figure S34 – Transient knockdown of Brd4 in Tau zebrafish mitigates microgliosis at 4dpf**

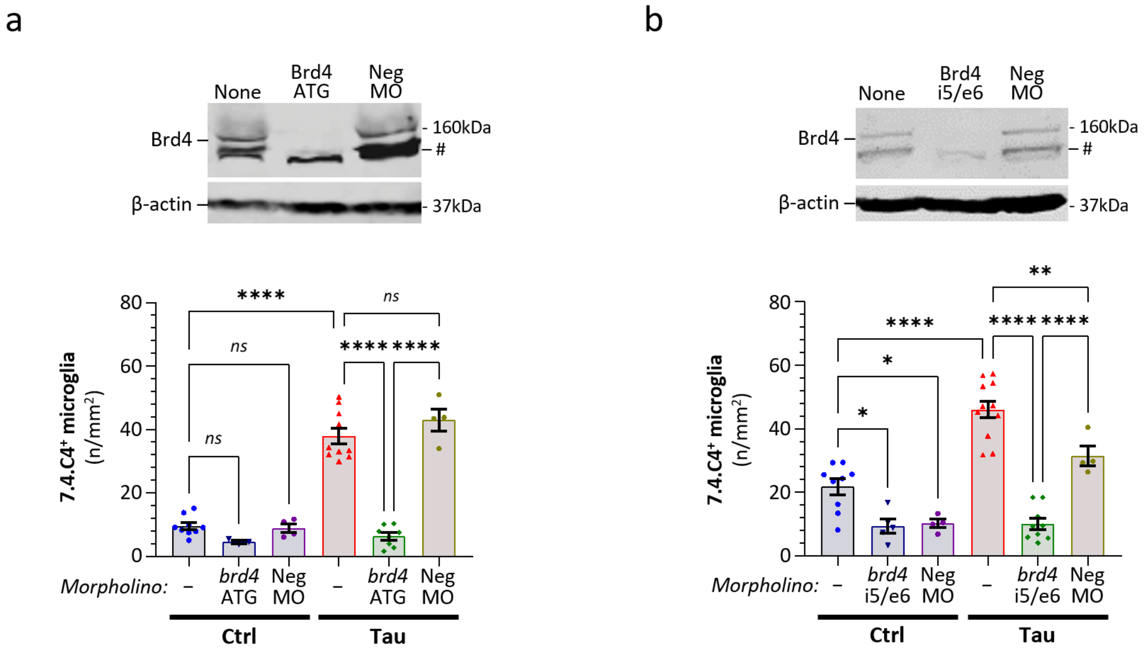


Morpholino oligonucleotides (MO) targeting brd4 expression were used to evaluate the role of Brd4 in microgliosis in Tau zebrafish. Two different MO were used to increase confidence that any observed effects were attributable to loss of Brd4 expression:

**a:** MO targeting the translational start site and adjacent 5'UTR of the zebrafish *brd4* mRNA. This is predicted to decrease translation of Brd4.

**b:** MO targeting the splice acceptor sequence at the boundary between intron 5 and exon 6 of the primary *brd4* transcript. This is predicted to skip exon 6 during splicing and introduce a frame shift mutation and premature stop codon into the mRNA, as the splice boundary phases of i5/e6 and i6/e7 differ with respect to the ORF.

MO were microinjected at the single-cell stage and both MO were compared with a non-targeting negative control MO (neg MO) to exclude effects caused by microinjection, injection buffer, or nonspecific effects attributable to MO exposure.

In the upper panels, western blots were made from lysates of 2 dpf control, *brd4* MO-microinjected and neg MO-microinjected zebrafish, and then probed with an antibody to zebrafish Brd4 (a generous gift from Dr. Igor Dawid, NIH). At this early developmental point, the antibody recognizes a protein of 160kDa corresponding to the expected size of Brd4, in addition to other smaller proteins whose identity is unclear (#). In each case, the 160kDa band disappeared after *brd4* MO microinjection but not neg MO injection, confirming that Brd4 was targeted by both MO.

In the lower panels, 7.4.C4-immunoreactive microglia were quantified in sections by immunohistochemistry at 4dpf, identically to Figure 2H – J, in Ctrl or Tau zebrafish that were untreated, or microinjected with either Brd4 or non-targeting MO. In each graph, data points show individual zebrafish, bars show mean ± SE. ****$p$<0.0001, **$p$<0.01, *$p$<0.05, 1-way ANOVA with Šidák multiple comparison test.

The large increase in microglial abundance in Tau zebrafish was prevented by Brd4 knockdown, suggesting Brd4 is the target through which (+)JQ1 prevents microgliosis in this model.

**Supplementary Figure S35 – Generation of a stable *brd4* null allele using Cas9/CRISPR**

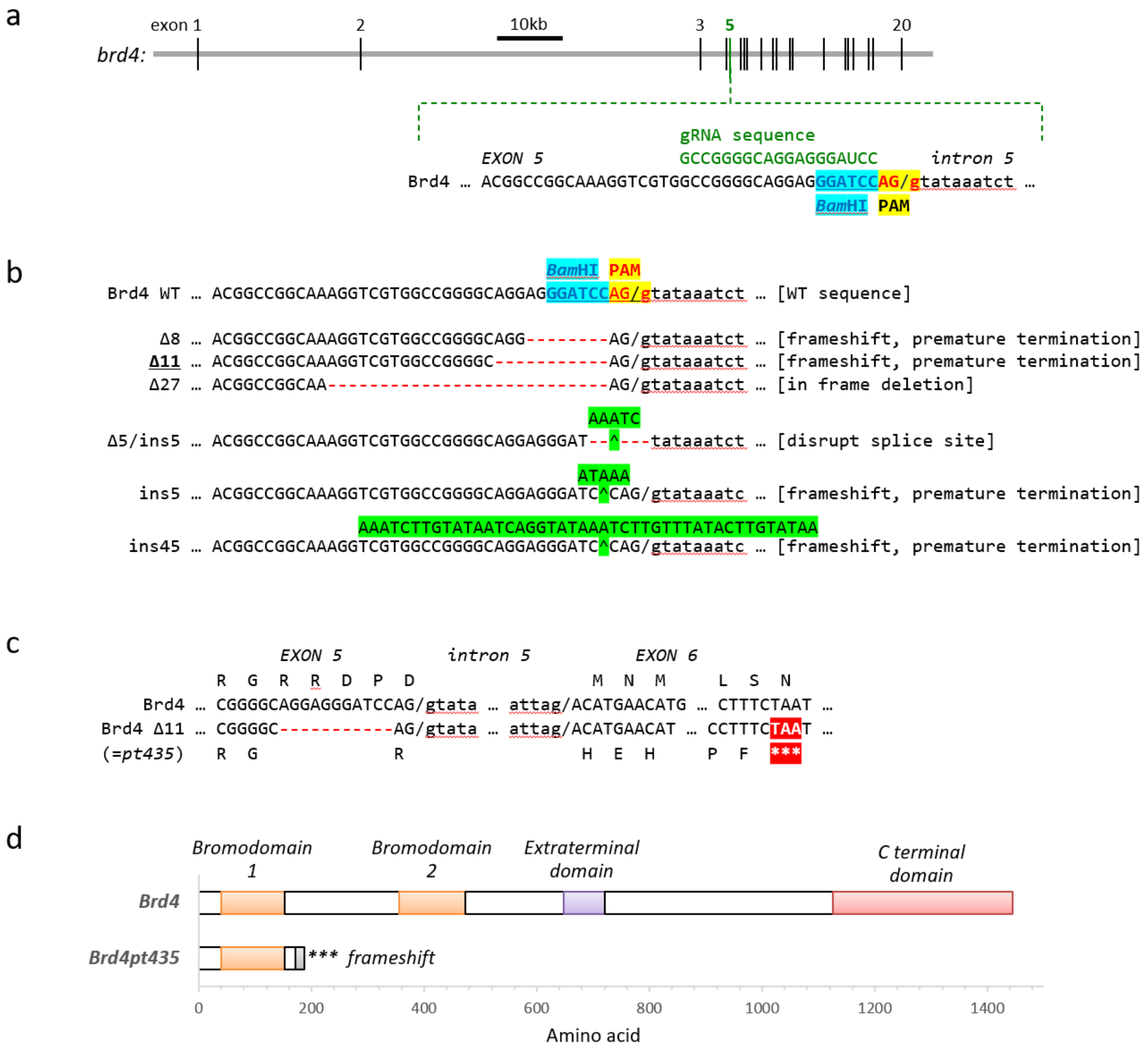


a: The *brd4*^Pt435 allele described in the paper was made using CRISPR/Cas9 genome editing. A gRNA sequence was identified to target a PAM site overlapping a *Bam*HI restriction enzyme site in exon 6 of the *brd4* gene. This was chosen to allow easy genotyping by showing loss of the *Bam*HI site in a PCR amplicon spanning the mutation, without necessitating DNA sequencing.

b: Multiple mutant alleles were recovered at the F1 stage, including small deletions and insertions as shown. The 11bp deletion was chosen for further studies, as it abolished Brd4 expression (Figure 10b).

c: The Pt435 11bp deletion allele causes a frameshift mutation leading to a premature stop codon within exon 6.

d: The predicted translation of the Pt435 allele leads to a severely truncated protein that ends after the first bromodomain of Brd4. Genetic studies suggest this is a null allele (see below) and not a dominant negative, as there is no phenotype in heterozygotes. The protein fragment may be unstable.

**Supplementary Figure S36 – Developmental phenotypes in *brd4*$^{-/-}$ zebrafish**

a

***Clutch #1***

| | brd4 genotype | | | |
|---|---|---|---|---|
| | +/+ | +/- | -/- | |
| **Observed** | **10** | **26** | **20** | χ2 = 1.800 |
| Mendelian | 14 | 28 | 14 | df = 2; *p* = 0.4067 |

***Clutch #2***

| | brd4 genotype | | | |
|---|---|---|---|---|
| | +/+ | +/- | -/- | |
| **Observed** | **14** | **25** | **15** | χ2 = 0.1484 |
| Mendelian | 13 | 27 | 14 | df = 2; *p* = 0.9285 |

***Clutch #3***

| | brd4 genotype | | | |
|---|---|---|---|---|
| | +/+ | +/- | -/- | |
| **Observed** | **11** | **24** | **13** | χ2 = 0.08348 |
| Mendelian | 12 | 24 | 12 | df = 2; *p* = 0.9591 |

b

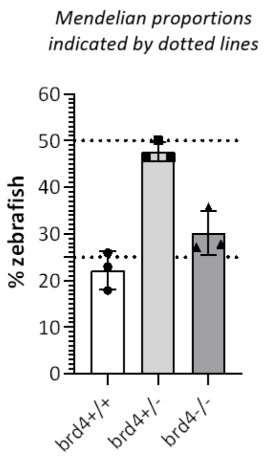


c

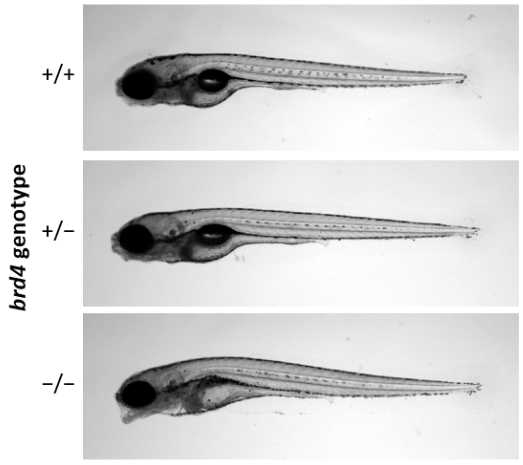


d

***Clutch #1***

| | brd4 genotype | | | |
|---|---|---|---|---|
| | +/+ | +/- | -/- | χ2 = 42.18 |
| Normal | 10 | 22 | 0 | df = 2 |
| **No swim bladder** | **0** | **4** | **20** | $p < 10^{-8}$ |

***Clutch #2***

| | brd4 genotype | | | |
|---|---|---|---|---|
| | +/+ | +/- | -/- | χ2 = 54.00 |
| Normal | 14 | 25 | 0 | df = 2 |
| **No swim bladder** | **0** | **0** | **15** | $p < 10^{-11}$ |

***Clutch #3***

| | brd4 genotype | | | |
|---|---|---|---|---|
| | +/+ | +/- | -/- | χ2 = 48.00 |
| Normal | 11 | 24 | 0 | df = 2 |
| **No swim bladder** | **0** | **0** | **13** | $p < 10^{-10}$ |

e

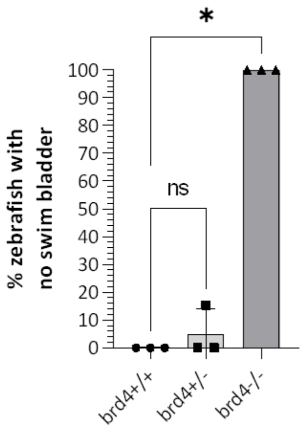


a: Genotypes from three independent clutches (same allele, different parents) of *brd4*$^{-/-}$ zebrafish at 5dpf. There was no significant difference between the observed genotypes and the expected Mendelian ratios.

b: Genotype proportions for the three clutches overall did not differ from the expected Mendelian ratios at 5dpf. However, no homozygous *brd4*$^{-/-}$ zebrafish adults were identified from >100 offspring of *brd4*$^{+/-}$ in-crosses that were genotyped, strongly suggesting that *brd4*$^{-/-}$ zebrafish are not viable to adulthood.

c: Morphological changes in *brd4*$^{-/-}$ zebrafish, including jaw region dysgenesis, pericardial edema, failure of swim bladder insufflation, and kyphotic spinal curvature.

d: Relationship between genotype and presence of a swim bladder. There was a strong statistically-significant association between *brd4*$^{-/-}$ genotype and failure of swim bladder insufflation in each of the three clutches analyzed in panel (a).

e: Genotype proportions for zebrafish from each of the three clutches without an observable swim bladder at 5dpf. The *brd4*$^{-/-}$ genotype was strongly associated with absent swim bladder. *$p$<0.05, 1-way ANOVA with Dunnett multiple comparisons test.

**Supplementary Figure S37 – Motor activity abnormalities in *brd4*$^{-/-}$ zebrafish**

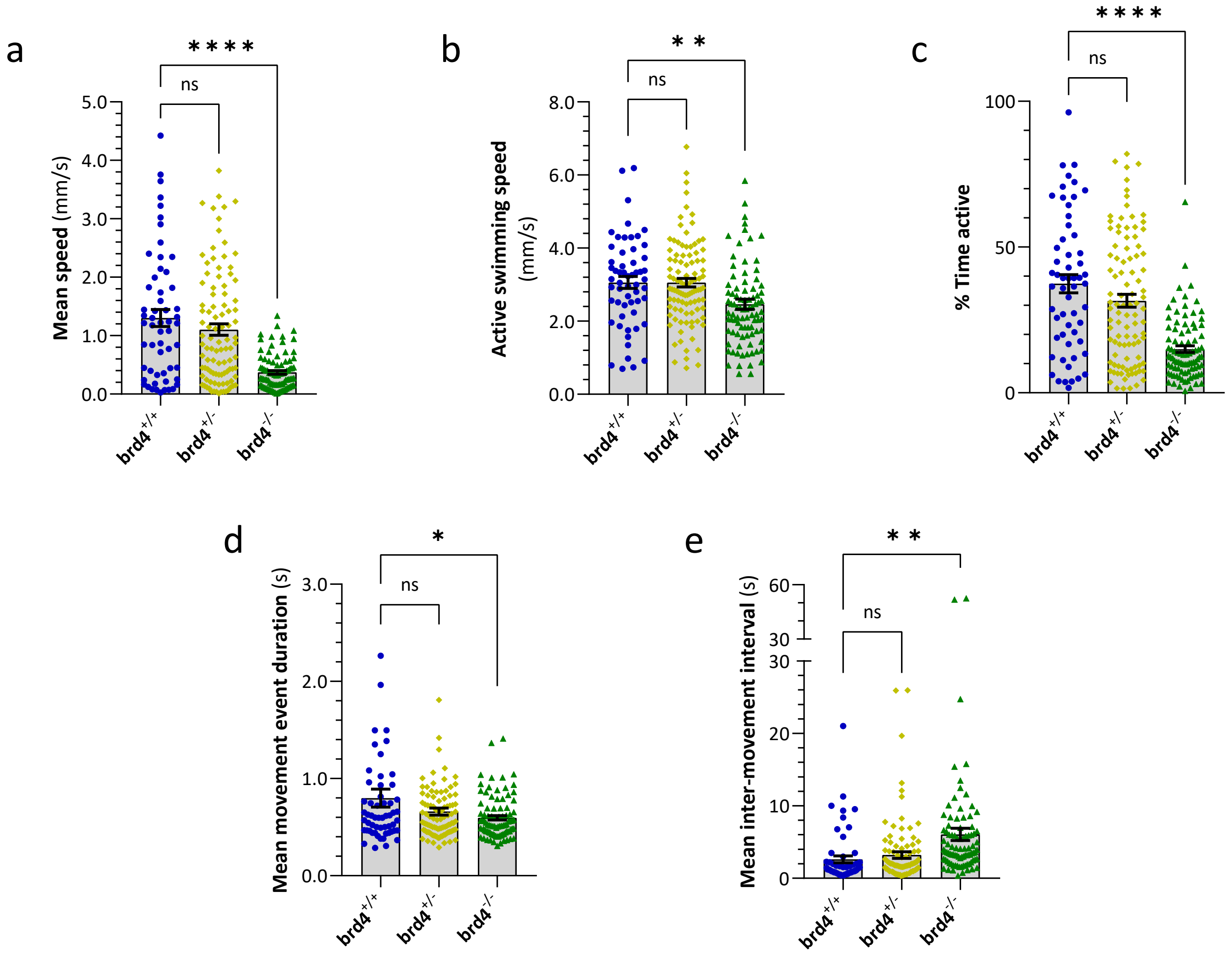


5dpf larvae resulting from *brd4*$^{+/-}$ in-crosses were analyzed in a 96-well plate motor activity assay similar to Figure 5a – 5h, and then each larva was genotyped by PCR amplification and *Bam*HI restriction digest for the *brd4*$^{Pt435}$ allele. In each graph, data points show individual zebrafish, bars show mean ± SE. ****$p$<0.0001, **$p$<0.01, *$p$<0.05, 1-way ANOVA with Dunnett multiple comparison test.

a: Mean speed (scalar distance traveled by zebrafish centroid/time of assay)
b: Active swimming speed (mean scalar speed of zebrafish centroid during movement events)
c: Time active % (proportion of video frame transitions at which centroid displacement occurred)
d: Mean movement event duration (mean duration of movement episodes)
e: Mean inter-movement interval (mean duration of stationary periods between movements)

Together with Supplementary Figure S36, these data suggest that analysis of *brd4* as a genetic modifier of the Tau phenotype would be difficult to interpret using homozygous *brd4*$^{-/-}$ mutants that have morphological and motor phenotypes of their own. However, heterozygous *brd4*$^{+/-}$ mutants that have no detectable abnormalities should provide a valid means to determine if decreased Brd4 expression modulates the phenotype in Tau zebrafish.

**Supplementary Figure S38 – Heterozygous *brd4*$^{+/-}$ mutations partially rescue motor phenotypes in Tau zebrafish**

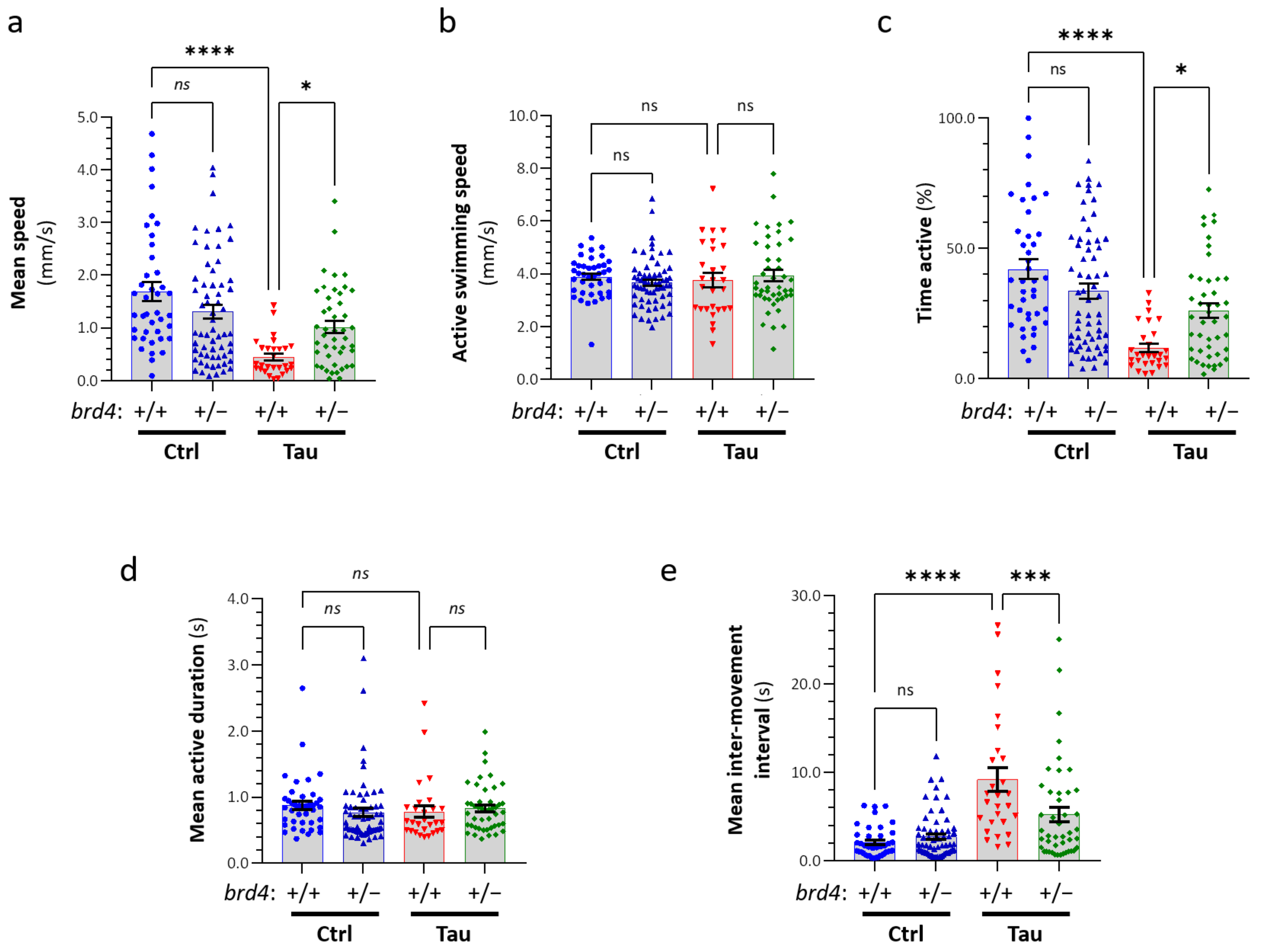


5dpf Tau and Ctrl larvae were analyzed in a 96-well plate motor activity assay similar to Figure 5a – 5h, and then each larva was genotyped by PCR amplification and *Bam*HI restriction digest of genomic DNA to detect the *brd4* deletion allele. In each graph, data points show individual zebrafish, bars show mean ± SE. ****$p$<0.0001, ***$p$<0.001, *$p$<0.05, 1-way ANOVA with Šidák multiple comparison test.

a: Mean speed (scalar distance traveled by zebrafish centroid/time of assay)
b: Active swimming speed (mean scalar speed of zebrafish centroid during movement events)
c: Time active % (proportion of video frame transitions at which centroid displacement occurred)
d: Mean movement event duration (mean duration of movement episodes)
e: Mean inter-movement interval (mean duration of stationary periods between movements)

These data show that heterozygous *brd4*$^{+/-}$ mutations partially rescue mean speed, % time active and inter-movement interval in Tau zebrafish. The modest abnormalities in active swimming speed and mean movement duration found in Tau zebrafish in Figure 5 were not detected in these larval clutches, which seemed to have a slightly weaker phenotype.

**Supplementary Table 22 – Exact *p*-values for Figure 10d – 10i**

| Panel | Statistical test | Comparison group 1 | Comparison group 2 | Adjusted *p* value |
|---|---|---|---|---|
| | | | | |
| 10d | One-way ANOVA with Šidák multiple comparisons test | Ctrl; *brd4*$^{+/+}$ | Ctrl; *brd4*$^{+/-}$ | 0.113707882660896 |
| | | Ctrl; *brd4*$^{+/+}$ | Tau; *brd4*$^{+/+}$ | 0.000000292123687 |
| | | Tau; *brd4*$^{+/+}$ | Tau; *brd4*$^{+/-}$ | 0.029082887963640 |
| | | | | |
| 10e | One-way ANOVA with Šidák multiple comparisons test | Ctrl; *brd4*$^{+/+}$ | Ctrl; *brd4*$^{+/-}$ | 0.999802006238610 |
| | | Ctrl; *brd4*$^{+/+}$ | Tau; *brd4*$^{+/+}$ | 0.000000000031921 |
| | | Tau; *brd4*$^{+/+}$ | Tau; *brd4*$^{+/-}$ | 0.362341368466568 |
| | | | | |
| 10f | One-way ANOVA with Šidák multiple comparisons test | Ctrl; *brd4*$^{+/+}$ | Ctrl; *brd4*$^{+/-}$ | 0.118543083544085 |
| | | Ctrl; *brd4*$^{+/-}$ | Ctrl; *brd4*$^{-/-}$ | 0.969533891959084 |
| | | Ctrl; *brd4*$^{+/+}$ | Ctrl; *brd4*$^{-/-}$ | 0.000009306670211 |
| | | Ctrl; *brd4*$^{+/+}$ | Tau; *brd4*$^{+/+}$ | 0.000000000102355 |
| | | Tau; *brd4*$^{+/+}$ | Tau; *brd4*$^{+/-}$ | 0.000000000019628 |
| | | Tau; *brd4*$^{+/-}$ | Tau; *brd4*$^{-/-}$ | 0.006322344844991 |
| | | Tau; *brd4*$^{+/+}$ | Tau; *brd4*$^{-/-}$ | <0.000000000000001 |
| | | | | |
| 10g | One-way ANOVA with Tukey multiple comparisons test | Ctrl; *brd4*$^{+/+}$ | Tau; *brd4*$^{+/+}$ | 0.000000000101563 |
| | | Ctrl; *brd4*$^{+/+}$ | Tau; *brd4*$^{+/-}$ | 0.000058615382397 |
| | | Tau; *brd4*$^{+/+}$ | Tau; *brd4*$^{+/-}$ | 0.002120097691076 |
| | | | | |
| 10h | One-way ANOVA with Tukey multiple comparisons test | Ctrl; *brd4*$^{+/+}$ | Tau; *brd4*$^{+/+}$ | 0.000000000019925 |
| | | Ctrl; *brd4*$^{+/+}$ | Tau; *brd4*$^{+/-}$ | 0.000005329924599 |
| | | Tau; *brd4*$^{+/+}$ | Tau; *brd4*$^{+/-}$ | 0.000009862682856 |
| | | | | |
| 10i | Two-way ANOVA with Šidák multiple comparisons test | Globus Pallidus Control | Globus Pallidus PSP | 0.317711657542679 |
| | | Substantia nigra control | Substantia nigra PSP | 0.998941139892130 |

**Supplementary Figure S39 – Heterozygous *brd4*$^{+/-}$ mutation does not rescue OKR deficits in Tau zebrafish**

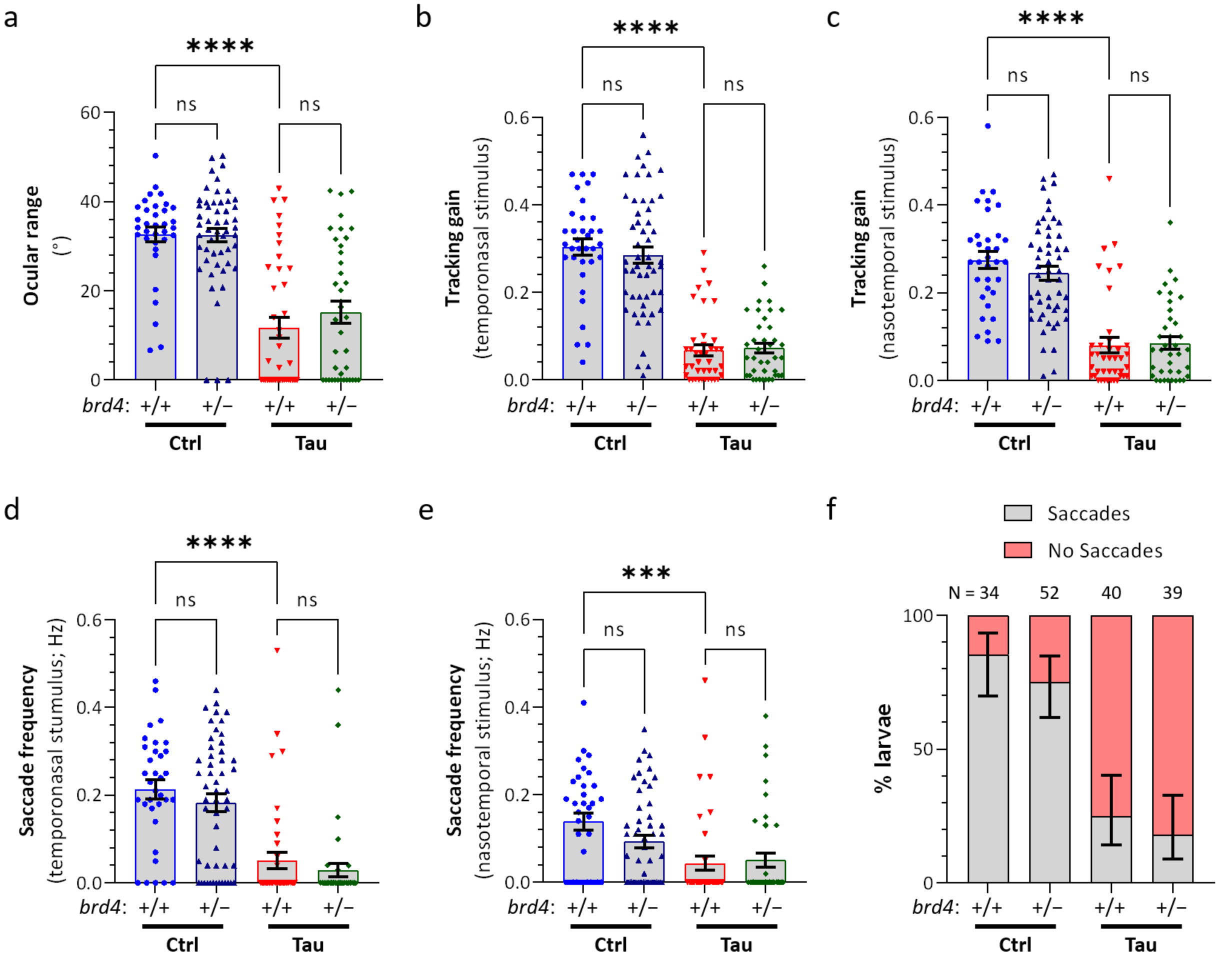


5dpf Tau and Ctrl larvae resulting from [Tg(*elavl3:gal4-vp16*); *brd4*$^{+/Pt435}$] x [Tg(*UAS:hsa.MAPT-p2a-nls-mCherry*) or Tg(*UAS:p2a-nls-mCherry*)] crosses were analyzed in OKR assays similar to Figure 6 and Supplementary Figures S15 and S27, and then each larva was genotyped by PCR amplification and *Bam*HI restriction digest for the *brd4*$^{Pt435}$ allele. In panels (a) – (e), data points show individual zebrafish, bars show mean ± SE. ****$p$<0.0001, ***$p$<0.001, 1-way ANOVA with Šidák multiple comparison test. In panel (f), bars show proportion ± 95% CI of zebrafish with and without saccadic movements during exposure to OKR stimuli.

Similar to findings with (+)JQ1 exposure, decreased Brd4 expression did not rescue OKR deficits in the Tau model at the time points evaluated.

**Supplementary Figure S40 – Rescue of synaptic puncta in Tau zebrafish with heterozygous *brd4*$^{+/-}$ mutations**

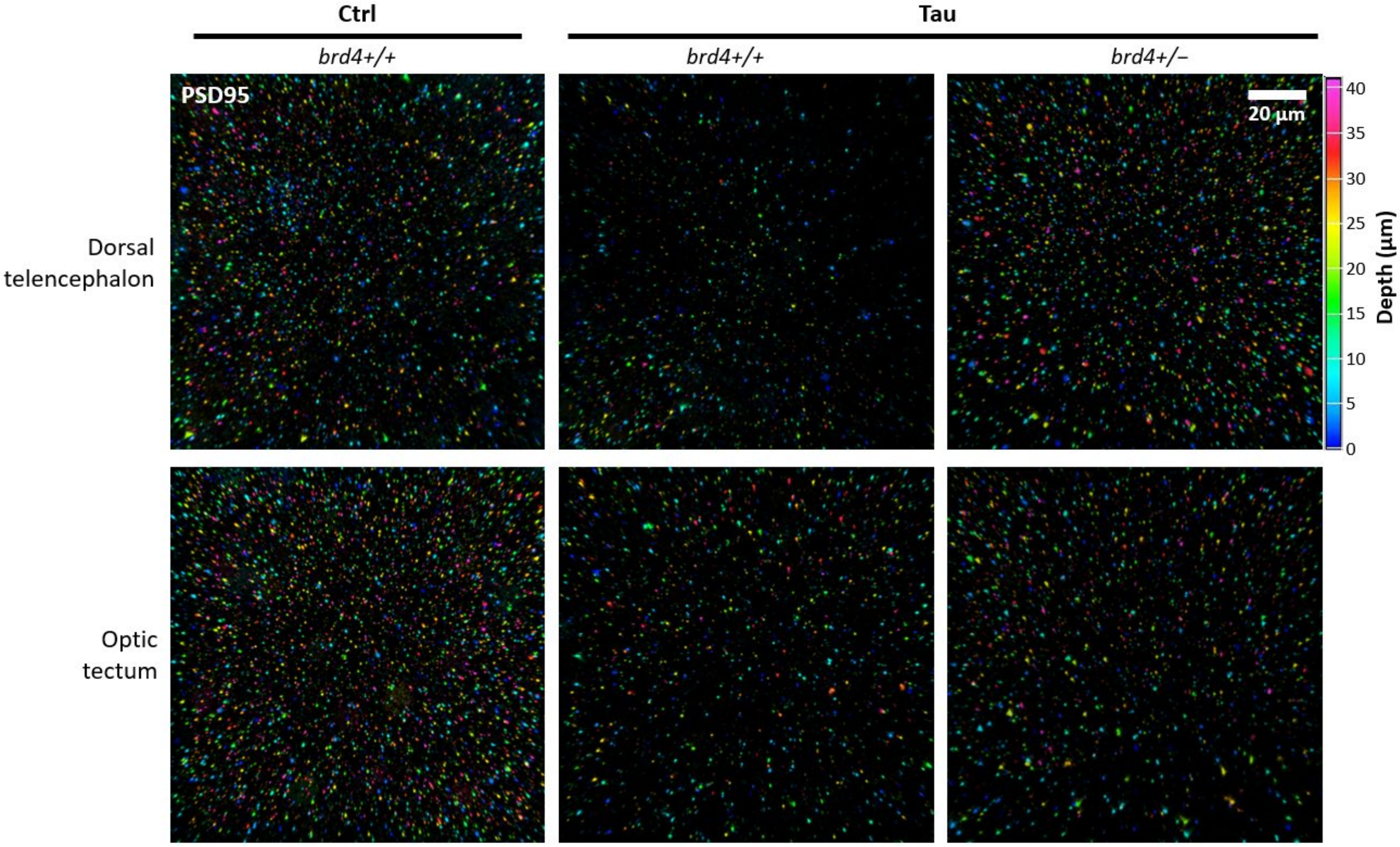


5dpf Tau and Ctrl larvae resulting from [Tg(*elavl3:gal4-vp16*); *brd4*$^{+/Pt435}$] x [Tg(*UAS:hsa.MAPT-p2a-nls-mCherry*) ***or*** Tg(*UAS:p2a-nls-mCherry*)] crosses were genotyped by PCR amplification and *Bam*HI restriction digest for the *brd4*$^{Pt435}$ allele, and then fixed for PSD95 immunofluorescence. These example images are alpha-blended and pseudocolored to show depth within the image stack, and accompany the quantitative analysis shown in Figure 10g – 10h.

**Supplementary Figure S41 – Brd4 expression in the nuclei of human substantia nigra microglia**

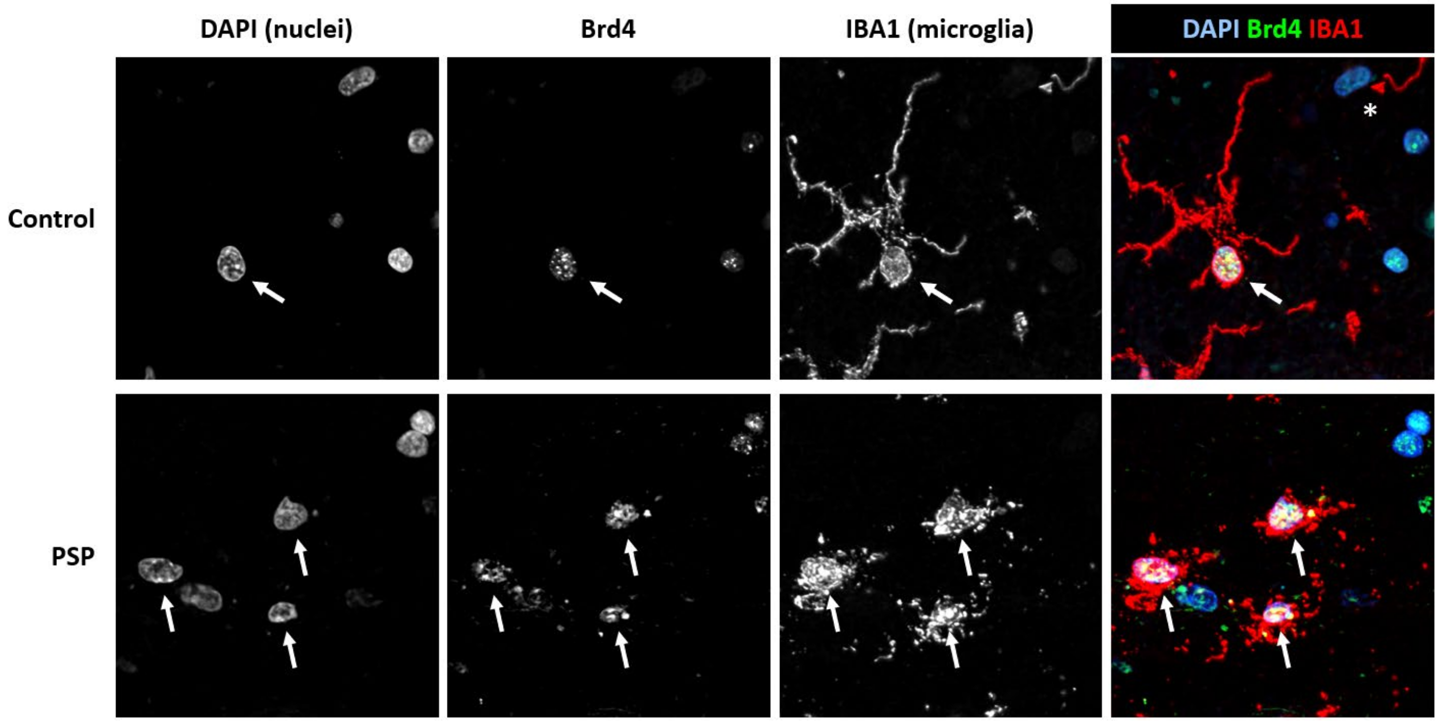


Individual fluorescence channels for the images shown in Figure 10j are separated and shown in monochrome for clarity. Image fields are shown from a control brain (top row) and a PSP brain (bottom row). Individual DAPI labeled nuclei (column 1) that were immunoreactive for Brd4 (column 2) and were located within Iba1-immunoreactive microglia (column 3) are indicated with white arrows in each image of a set. The overlaid pseudocolored images are shown in column 4.

**Supplementary Figure S42 – Brd4 expression in microglia from multiple control and PSP cases**

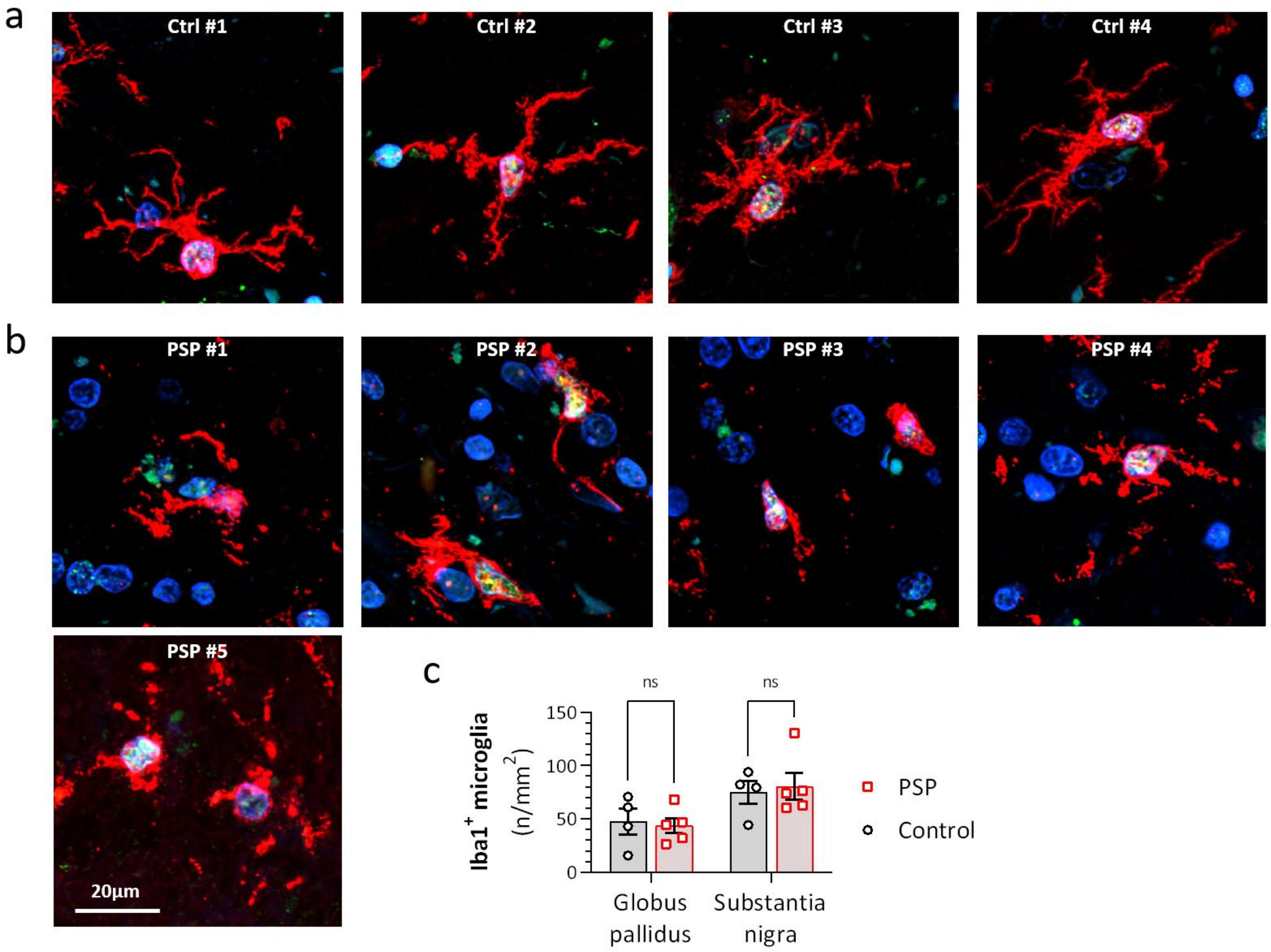


Human control and PSP brain sections from two brain regions characteristically affected by PSP pathology – the substantia nigra and globus pallidus – were immunolabeled for Brd4 (green), Iba1 (microglial marker; red) and counter-labeled with DAPI (nuclei; blue). Images were acquired by confocal microcopy.

a: Examples of Brd4$^+$, Iba1$^+$ microglia are shown from the substantia nigra of each of 4 control brains

b: Examples of Brd4$^+$, Iba1$^+$ microglia are shown from the substantia nigra of each of 5 PSP brains

c: There was no significant overall increase in the density of Iba1-labeled microglia in either the substantia nigra or the globus pallidus of PSP brains in this small sample, despite the characteristic morphological differences (retracted processes, prominent perinuclear cytoplasm) suggestive of activation seen in the images from PSP brains.

## References


1. Van Laar, V.S. et al. α-Synuclein amplifies cytoplasmic peroxide flux and oxidative stress provoked by mitochondrial inhibitors in CNS dopaminergic neurons in vivo. *Redox Biol* **37**, 101695 (2020).
2. Zhou, Y., Cattley, R.T., Cario, C.L., Bai, Q. & Burton, E.A. Quantification of larval zebrafish motor function in multiwell plates using open-source MATLAB applications. *Nat Protoc* **9**, 1533-48 (2014).
3. Cario, C.L., Farrell, T.C., Milanese, C. & Burton, E.A. Automated measurement of zebrafish larval movement. *J Physiol* **589**, 3703-8 (2011).
4. Scheetz, S.D. et al. An open-source method to analyze optokinetic reflex responses in larval zebrafish. *J Neurosci Methods* **293**, 329-337 (2018).
5. Hartnett, K.A. et al. NMDA receptor-mediated neurotoxicity: a paradoxical requirement for extracellular Mg2+ in Na+/Ca2+-free solutions in rat cortical neurons in vitro. *J Neurochem* **68**, 1836-45 (1997).
6. Goshi, N., Morgan, R.K., Lein, P.J. & Seker, E. A primary neural cell culture model to study neuron, astrocyte, and microglia interactions in neuroinflammation. *Journal of Neuroinflammation* **17**, 155 (2020).
