# Supplementary material for: A human Tau expressing zebrafish model of progressive supranuclear palsy identifies Brd4 as a regulator of microglial synaptic elimination
Source: Nat Commun. 2024 Sep 18;15:8195. doi: 10.1038/s41467-024-52173-0 (PMC11410960; doi:10.1038/s41467-024-52173-0)
Supplement: Supplementary file 3 — Description of Additional Supplementary Files [file 41467_2024_52173_MOESM3_ESM.pdf]

Description of Additional Supplementary Files

File Name: Supplementary Movie 1
Description: Example infrared videomicrography recordings of optokinetic responses in Ctrl and Tau zebrafish at 5 days post-fertilization, shown at 3 x actual speed for clarity. The video is a collage of two separate recordings for Ctrl and Tau zebrafish, made in response to identical stimuli; a linear rendering of the stimulus is shown at the top of the image for comparison. Quantitative analysis in a statistically robust sample of zebrafish is shown in Figure 6.
