# Supplementary material for: A human Tau expressing zebrafish model of progressive supranuclear palsy identifies Brd4 as a regulator of microglial synaptic elimination
Source: Nat Commun. 2024 Sep 18;15:8195. doi: 10.1038/s41467-024-52173-0 (PMC11410960; doi:10.1038/s41467-024-52173-0)
Supplement: Supplementary file 5 — Reporting Summary [file 41467_2024_52173_MOESM5_ESM.pdf]

nature portfolio

Corresponding author(s): Edward A. Burton

Last updated by author(s): Jul 31, 2024

Reporting Summary

Nature Portfolio wishes to improve the reproducibility of the work that we publish. This form provides structure for consistency and transparency in reporting. For further information on Nature Portfolio policies, see our Editorial Policies and the Editorial Policy Checklist.

Statistics

For all statistical analyses, confirm that the following items are present in the figure legend, table legend, main text, or Methods section.

n/a	Confirmed	
☐	☒	The exact sample size (*n*) for each experimental group/condition, given as a discrete number and unit of measurement
☐	☒	A statement on whether measurements were taken from distinct samples or whether the same sample was measured repeatedly
☐	☒	The statistical test(s) used AND whether they are one- or two-sided<br>*Only common tests should be described solely by name; describe more complex techniques in the Methods section.*
☐	☒	A description of all covariates tested
☐	☒	A description of any assumptions or corrections, such as tests of normality and adjustment for multiple comparisons
☐	☒	A full description of the statistical parameters including central tendency (e.g. means) or other basic estimates (e.g. regression coefficient) AND variation (e.g. standard deviation) or associated estimates of uncertainty (e.g. confidence intervals)
☐	☒	For null hypothesis testing, the test statistic (e.g. *F*, *t*, *r*) with confidence intervals, effect sizes, degrees of freedom and *P* value noted<br>*Give P values as exact values whenever suitable.*
☒	☐	For Bayesian analysis, information on the choice of priors and Markov chain Monte Carlo settings
☒	☐	For hierarchical and complex designs, identification of the appropriate level for tests and full reporting of outcomes
☒	☐	Estimates of effect sizes (e.g. Cohen's *d*, Pearson's *r*), indicating how they were calculated

Our web collection on statistics for biologists contains articles on many of the points above.

Software and code

Policy information about availability of computer code

Data collection	Fluoview 4.2; NIS Elements 5.20; LiCOR Odyssey 3.0; FlyCapture 2.10.3;
Data analysis	GraphPad Prism 10; MATLAB R2020b; Microsoft Office 365; Fluoview 4.2; NIS Elements 5.20; Image J 1.54; LSRTrack (Cario 2011); LSRAnalyze (Cario 2011); OKRtrack (Scheetz 2018); HiSpeedTracking (Hossainian 2022); LiCOR Odyssey 3.0

For manuscripts utilizing custom algorithms or software that are central to the research but not yet described in published literature, software must be made available to editors and reviewers. We strongly encourage code deposition in a community repository (e.g. GitHub). See the Nature Portfolio guidelines for submitting code & software for further information.

Data

Policy information about availability of data

All manuscripts must include a data availability statement. This statement should provide the following information, where applicable:

- Accession codes, unique identifiers, or web links for publicly available datasets
- A description of any restrictions on data availability
- For clinical datasets or third party data, please ensure that the statement adheres to our policy

Source data files are provided for every figure.

Research involving human participants, their data, or biological material

Policy information about studies with human participants or human data. See also policy information about sex, gender (identity/presentation), and sexual orientation and race, ethnicity and racism.

Reporting on sex and gender	Post mortem tissue is used in figure 6J, K. The biological sex of the participants is stated in the manuscript.
Reporting on race, ethnicity, or other socially relevant groupings	These data were not available to this study, tissue was from anonymized cases per CORID approval.
Population characteristics	The age at death and neuropathological diagnosis are stated in the manuscript.
Recruitment	The subjects were all patients at UPMC who had autopsies after death and were found to have PSP or no evidence of neurological disease.
Ethics oversight	All human tissue studies were carried out with approval from the University of Pittsburgh Committee for Oversight of Research and Clinical Training Involving Decedents (CORID)

Note that full information on the approval of the study protocol must also be provided in the manuscript.

Field-specific reporting

Please select the one below that is the best fit for your research. If you are not sure, read the appropriate sections before making your selection.

☒ Life sciences ☐ Behavioural & social sciences ☐ Ecological, evolutionary & environmental sciences

For a reference copy of the document with all sections, see nature.com/documents/nr-reporting-summary-flat.pdf

Life sciences study design

All studies must disclose on these points even when the disclosure is negative.

Sample size	For comparisons between groups, sample sizes were determined by power analysis using effect size and variation estimates from pilot studies. For the screen, the sample size was based on custom analyses that are described fully in the manuscript and shown in figure 7a, 7b to optimize assay throughput against the utility of the assay outputs to detect meaningful phenotypic rescue.
Data exclusions	Experiments or samples that failed technically and did not yield data were not included. For the screen, data points failing QC are excluded from rescue calculations based on the performance of controls in same time bin. The methodology and algorithm are described in detail in the supplemental data.
Replication	Three independent biological replicates were completed for each experiment (replicates are sometime shown combined for clarity, for example figure 5c, if controls showed identical outcomes between replicate, as shown in the individual replicates in supplemental figure S12). The screen involved 12 replicate zebrafish for each chemical, but chemicals were not re-tested in replicate assays unless there was a technical failure.
Randomization	For the screen and subsequent (+)JQ1 exposure experiments, after sorting by Tau genotype, zebrafish larvae were randomly assigned to chemical treatment or control groups. Other analyses compared Tau and controls or Brd4 knockouts with controls, so larvae were assigned to experimental groups retrospectively according to their genotype.
Blinding	The screen was completed blinded to chemical identity. All methods using manual counting, for example figure 2e, were completed blinded to experimental group. Blinding was not used when automated, unbiased analyses were employed as in figure 5c.

Reporting for specific materials, systems and methods

We require information from authors about some types of materials, experimental systems and methods used in many studies. Here, indicate whether each material, system or method listed is relevant to your study. If you are not sure if a list item applies to your research, read the appropriate section before selecting a response.

Materials & experimental systems

n/a	Involved in the study
	☒ Antibodies
☒	☐ Eukaryotic cell lines
☒	☐ Palaeontology and archaeology
	☒ Animals and other organisms
☒	☐ Clinical data
☒	☐ Dual use research of concern
☒	☐ Plants

Methods

n/a	Involved in the study
☒	☐ ChIP-seq
☒	☐ Flow cytometry
☒	☐ MRI-based neuroimaging

Antibodies

Antibodies used	These are listed in the methods section of the manuscript along with supplier and catalog number, or lab of origin.
Validation	> Human Tau, phospho-Tau, truncated Tau and misfolded Tau antibodies have been extensively validated and epitope mapped in the literature and have been used in multiple previous studies on human and mouse tissue. By comparing Tau zebrafish and controls, we show these antibodies do not cross react with any zebrafish protein on IHC or western blot, allowing us to conclude that we detected the relevant forms of the human protein in the transgenic animals.<br>> Antibodies recognizing transgene products such as mCherry did not detect any signal in non-transgenic zebrafish.<br>> The zebrafish Brd4 antibody did not detect any signal in genetic Brd4 knockout zebrafish in this study<br>> Other antibodies used to detect endogenous zebrafish proteins (TH, GAD, PSD95, SYP, 7.4.C4) have been validated and characterized in previous studies. All antibodies recognized bands of expected size on Western blot and labeled the expected structures on sections.

Animals and other research organisms

Policy information about studies involving animals; ARRIVE guidelines recommended for reporting animal research, and Sex and Gender in Research

Laboratory animals	Danio rerio (zebrafish) strain AB and transgenic lines as detailed in the manuscript.
Wild animals	N/A
Reporting on sex	Larval zebrafish < 15 post-fertilization were analyzed in the experiments shown; zebrafish sex is not determined or established at this developmental point, and so the study included subjects with potential to differentiate into either male or female zebrafish.
Field-collected samples	N/A
Ethics oversight	All zebrafish studies were carried out with approval from the University of Pittsburgh Institutional Animal Care and Use Committee (IACUC)

Note that full information on the approval of the study protocol must also be provided in the manuscript.
